# The shrimp superfamily Sergestoidea: a global phylogeny with definition of new families and an assessment of the pathways into principal biotopes

**DOI:** 10.1098/rsos.170221

**Published:** 2017-09-06

**Authors:** A. L. Vereshchaka

**Affiliations:** P. P. Shirshov Institute of Oceanology, Russian Academy of Sciences, Moskva 117997, Russia

**Keywords:** shrimps, Crustacea, phylogeny, pelagic, evolution

## Abstract

The phylogenetic analysis of Sergestoidea based on 253 morphological characters and encompassing all 99 valid species confirmed all previously recognized genus-level clades. Analysis retrieved five major robust clades that correspond to families Luciferidae, Sergestidae, Acetidae fam.n., Sicyonellidae fam.n. and Petalidiumidae fam.n. Synonymy, emended diagnoses and composition of revealed family-level clades are provided. Three types of morphological characters were important in the phylogeny of the Sergestoidea: general external characters, copulatory organs, and photophores. Novel metrics to quantify the contribution of these character types were tested. General external characters were significant in supporting the major clades (80% of the families and nearly half of the genera). Copulatory organ characters and photophores greatly supported the medium-level clades: *Lucifer, Belzebub*, *Petalidium, Neosergestes, Challengerosergia* (copulatory organ characters) and *Lucensosergia*, *Challengerosergia, Gardinerosergia*, *Phorcosergia* (photophores). An evolutionary model of the Sergestoidea showing their pathways into their principal biotopes is proposed: the major clades evolved in a vertical direction (from epi- to bathypelagic); further divergence at the genus level occurred within vertical zones in a horizontal direction, with the invasion of the benthopelagic and neritic (shelf and estuarine) habitats and speciation within these domains.

## 1. Introduction

The order Decapoda includes two suborders, Pleocyemata and Dendrobranchiata, the latter encompassing two superfamilies, Sergestoidea and Penaeoidea. The Sergestoidea hereby represents a significant part of the decapod phylogenetic tree, while phylogeny and even taxonomy (e.g. the proper position of *Lucifer*) within this superfamily remains unclear [1]. This is partly because of the poorly resolved systematics of two speciose groups (genera) in the family Sergestidae (see details in [2–4]), previously known as *Sergestes* and *Sergia* (over 70 species). Species of the Sergestidae are among the most common in many marine ecosystems and important targets of fisheries in some areas [5–7]. Despite their importance, the Sergestoidea are still poorly understood with regard to higher level classification and phylogenetic relationships.

During the last two decades, intensive studies of the *Sergestes* and *Sergia* groups have resulted in two morphological revisions [2,3] and further phylogenetic revisions, which have yielded eight new genera [4,8]. The revision of the other genera of Sergestidae, i.e. *Petalidium*, *Acetes*, *Peisos* and *Sicyonella*, was made later and showed the monophyly of these genera and the synonymy of *Acetes* and *Peisos* [9,10]. Finally, scanning electron microscopy of the Luciferidae has allowed an estimation of homologies between copulatory structures of this family and the rest of the Sergestoidea that has, in turn, enabled a phylogenetic revision of the genus *Lucifer* and splitting this genus into two monophyletic genera: *Lucifer* and *Belzebub* [11]. The phylogenetic study of the whole Sergestoidea at species level can now be completed by assembling the rich dataset of morphological characters (approx. 250) that have been coded during previous studies, and this is the first objective of this paper.

Most previous phylogenetic analyses in various groups of Sergestoidea have led to changes in the classification and taxonomy at the genus level. The robustness of the revealed genus clades may now be tested on a richer material and new clades of the genus or family levels may be found. Therefore, the second objective of this paper is to establish a natural phylogenetic classification of the superfamily and to provide emended diagnoses/keys for the major taxa.

Previous studies have shown that the Sergestoidea is a group exhibiting independent phylogenetic traits in three types of morphological characters [4,10,11]: (i) general external characters typical for most crustaceans, (ii) copulatory characters related to spermatophore transfer and grasping of the female (male clasping organ and petasma) and (iii) photophores. The third objective of the paper is to assess the contribution of these character types to the robustness of the clades.

Finally, the Sergestoidea is a group widely distributed in all temperate and tropical waters and at different depth strata, from epi- to bathypelagic. The last objective of this paper is to assess evolutionary scenarios including the colonization of principal oceanic biotopes.

## 2. Material and methods

### 2.1. Morphological analysis

The material used for this study is from the extensive collections of pelagic crustaceans taken during the Danish ‘Dana I’ (1920–1922) and ‘Dana II’ (1928–1930) Expeditions stored at the Natural History Museum of Denmark; some type specimens were requested from other museums (electronic supplementary material, appendix S1). All valid species of Sergestoidea have been included as terminal taxa, with updated synonymies (electronic supplementary material, appendix S2). As outgroups we tested two species of the sister superfamily Penaeoidea, pelagic *Gennadas parvus* Spence Bate, 1881 (family Benthesicymidae) and benthic *Penaeus monodon* Fabricius, 1798 (Penaeidae). Character state scoring for each species was derived from examination of the type material (electronic supplementary material, appendix S1).

We used the characters described and figured in a series of preceding papers [4,9–11], a total of 253 edited characters (electronic supplementary material, appendix S3) for 101 terminal taxa. The data matrix is presented in the electronic supplementary material, appendix S4.

**Figure 1.**
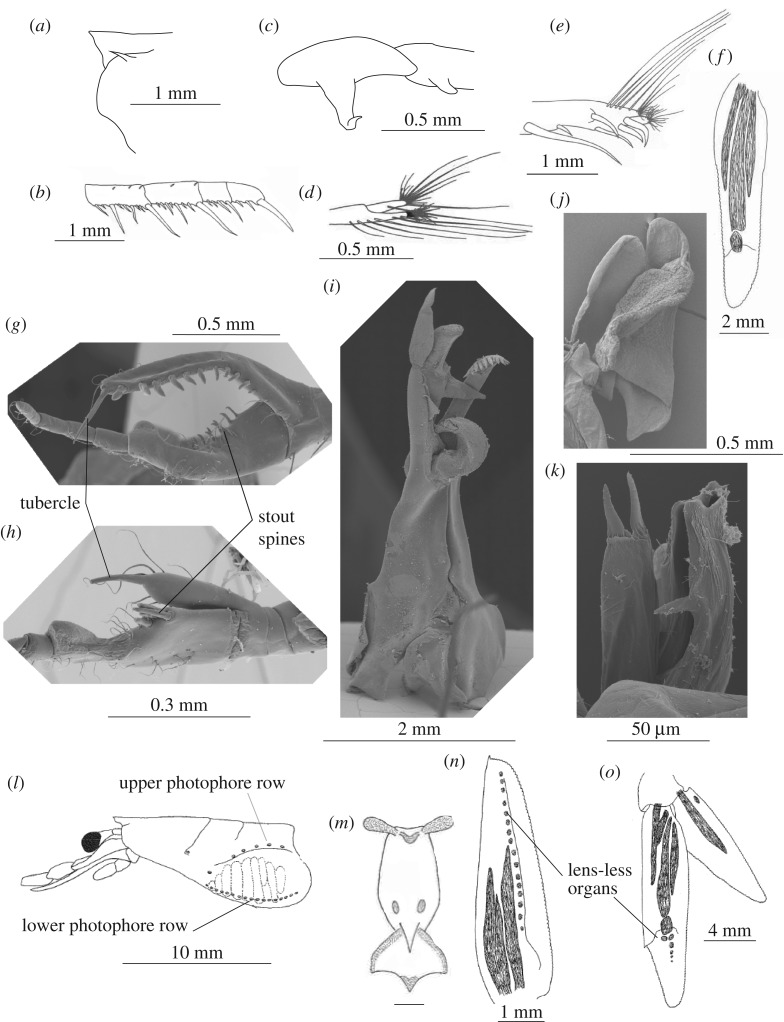
Character types in Sergestoidea. General external characters: frontal margin of carapace, *Sergestes atlanticus* (*a*); dactyl of third maxilliped, *Parasergestes vigilax* (*b*); basal part of second pereopod, *Neosergestes edwardsi* (*c*); chela of second pereopod, *Deosergestes corniculum* (*d*); apical part of third pereopod, *Deosergestes corniculum* (*e*); uropodal exopod, *Allosergestes sargassi* (*f*). Copulatory characters: male clasping organs in *Petalidium obesum* (*g*) and *Lucensosergia lucens* (*h*); petasmata in *Deosergestes corniculum* (*i*), *Acetes marinus* (*j*) and *Lucifer typus* (*k*). Photophores: lateral lens-bearing organs on carapace, *Prehensilosergia prehensilis* (*l*); the organ of Pesta, *Deosergestes corniculum* (*m*); lens-less organs on scaphocerite of *Gardinerosergia kensleyi* (*n*) and uropod of *Phorcosergia potens* (*o*).

Characters were divided into three groups (figure 1): (i) general external characters (0–115; here and below, numbers in parenthesis after characters indicate the position of characters in electronic supplementary material, appendix S3), (ii) copulatory organs (116–189) including the petasma and the clasping organ (modified part of Antenna I) and (iii) photophores (190–248). All characters were unordered (non-additive) and equally weighted, missing data were scored unknown.

Data were handled and analysed using a combination of programs and maximum-parsimony settings: Winclada/Nona, NDE (Nexus Data Editor) and TNT [12–14]. Trees were generated in TNT with 30 000 trees in memory, under the ‘traditional search’ mode with the following settings: 150 replicates, 200 trees to save per replication and 10 000 replicates, 200 trees to save per replication. Relative stability of clades was assessed by standard bootstrapping (with replacement) with 10 000 pseudoreplicates and by Bremer support (algorithm TBR, saving up to 10 000 trees up to three steps longer). I considered the clades statistically significant if they were simultaneously supported by the bootstrap values greater than 80% and by the Bremer values (decay index) greater than 3.

### 2.2. Novel metrics to quantify contribution of a character type to the clade support and divergence within a clade

**Figure 2.**
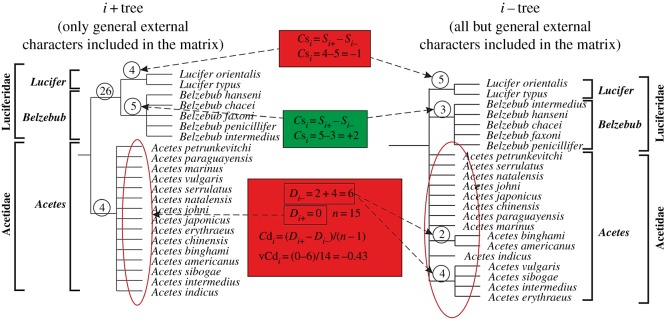
Calculation of new metrics to assess the role of various character sets in phylogeny, exemplified by the clades *Lucifer*, *Belzebub* and *Acetes*. Bremer support values are in circles. Figure shows calculations of Cs*_i_* and Cd*_i_*, negative values marked in red, positive values in green. Cs*_i_* values for the clades Luciferidae and Acetidae are not shown; they are 26 and 4, respectively (*S_i_*_−_ = 0 in both cases, as the clades are not supported).

In order to quantify the contribution (*C*), of a character type *i* to a support (*S*) of a selected clade, a new metric Cs*_i_* = *S_i+_* − *S_i−_* (figure 2) is offered, where *S_i_*_+_ is a support of the clade, if only the type *i* characters are included in the matrix, *S_i_*_−_ is the support of the clade, if all but the type *i* characters are included in the matrix. If Cs*_i_* is positive, the type *i* characters positively contribute to the clade support; greater Cs*_i_* values indicate greater contribution. I use the Cs*_i_* metric to assess the contribution of different character types to support of the clades at the genus and family levels: Cs_G_ for general (G) external characters, Cs_C_ for copulatory (*C*) organs and Cs_Ph_ for photophores (Ph).

For example, we test contribution of external characters (G). Step 1: we include only this character type in the matrix and calculate a clade support *S*_G+_. Step 2: we use another matrix with all BUT external characters included and calculate the clade support *S*_G−_. Step 3: we calculate Cs_G_ = *S*_G+_ − *S*_G−_ and thus see how the clade support has changed. If the clade support decreases (Cs_G_ negative), external characters contribute positively. If the clade support increases, external characters contribute negatively and erode the phylogenetic tree.

In order to quantify the contribution (*C*), of a character type *i* to a divergence (*D*) within a clade, a new metric Cd*_i_* = *D_i+_*/(*n* − 1) − *D_i−_*/(*n* − 1) is offered (figure 2), where *D_i+_* is a sum of support in resolved branches within the clade, if only the type *i* characters are included in the matrix, *D_i−_* is a sum of support in resolved branches within the clade, if all but the type *i* characters are included in the matrix, *n* is a number of terminal taxa within the clade. If Cd*_i_* is positive, the type *i* characters positively contribute to divergence within the clade; greater Cd values indicate greater contribution. I use the Cd*_i_* metric to assess the contribution of different character types to the divergence of the clades at the genus levels, e.g. to speciation: Cd_G_ for general external characters, Cd_C_ for copulatory organs and Cd_Ph_ for photophores. Both Cs*_i_* and Cd*_i_* metrics are calculated on the basis of the Bremer and bootstrap support. If any of the calculations gives a positive result, a contribution of a type *i* characters is considered to be positive.

Clade support was calculated using the same combination of programs and the same settings as in morphological analysis.

## 3. Results

### 3.1. The consensus tree and supported clades

**Figure 3.**
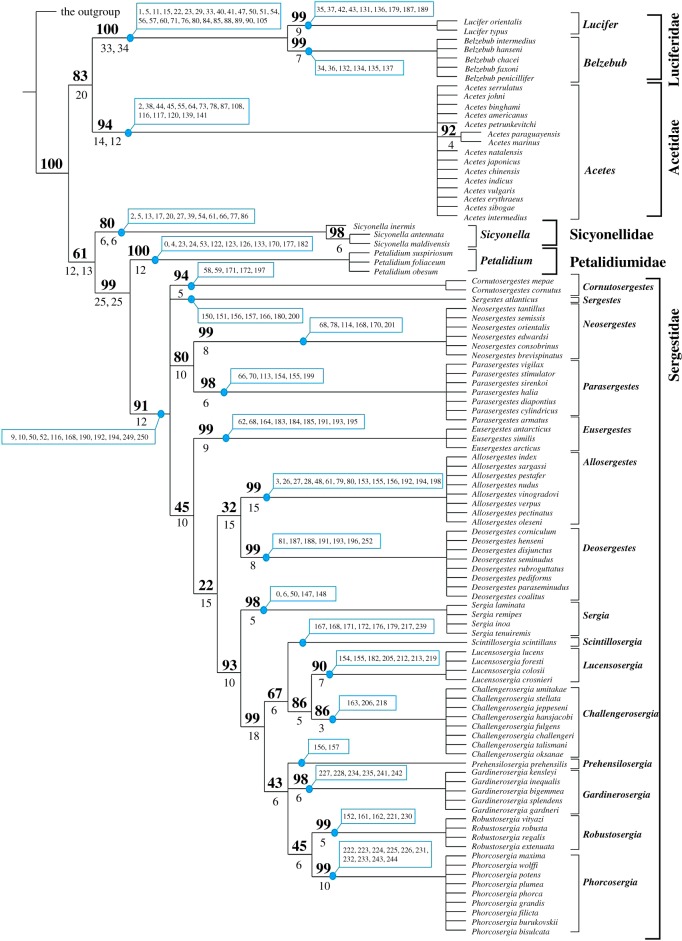
The strict consensus tree and supported clades retrieved after analyses with *Gennadas parvus* and *Penaeus monodon* as outgroups. The bootstrap support (bold numbers above the clade) and the Bremer support (numbers below the clade) are mostly identical with both outgroups; if not, they are separated by comma and refer to *G. parvus* and *P. monodon* as outgroups, respectively. Synapomorphies are identical and in blue, numbers refer to electronic supplementary material, appendix S3.

Both analyses with *Gennadas parvus* and *Penaeus monodon* as outgroups retrieved 240 minimal length trees, with 470 and 467 steps, respectively. The topologies of both strict consensus trees were identical and support of the clades was also identical or very similar (figure 3). Two major sister clades were revealed: Acetidae + Luciferidae and Sicyonellidae + Petalidiumidae + Sergestidae. The first clade received high Bremer (20) and bootstrap (83%) support, while the second clade received only Bremer support (12/13).

The clade Luciferidae was sister to Acetidae, and both were robust: 33/34 Bremer and 100% bootstrap support and 12/14 Bremer and 94% bootstrap support, respectively. The clade Luciferidae was divided into *Lucifer* (9 Bremer and 99% bootstrap support) and *Belzebub* (7 Bremer and 99% bootstrap support). Within Acetidae, a single-clade *Acetes marinus* + *Acetes paraguayensis* received high Bremer (4) and bootstrap (92%) support.

Within the clade Sicyonellidae + Petalidiumidae + Sergestidae, Sicyonellidae was sister to Petalidiumidae + Sergestidae, and both were robust: 6 Bremer and 80% bootstrap support versus 25 Bremer and 99% bootstrap support, respectively. Within Sicyonellidae, the clade *Sicyonella antennata* + *Sicyonella maldivensis* received high Bremer (6) and bootstrap (98%) support.

Petalidiumidae was sister to Sergestidae, and both were robust: 12 Bremer and 100% bootstrap support versus 12 Bremer and 91% bootstrap support, respectively. Petalidiumidae was not resolved, while the Sergestidae received significant resolution.

The Sergestidae included several robust major clades (figure 3): (i) *Neosergestes* +* Parasergestes*, (ii) eight genera of the former *Sergia* [4], seven with the root ‘*sergia*’, (iii) seven genera of the former *Sergia* [4]) with the exception of *Sergia* and (iv) *Lucensosergia+ Challengerosergia*. All generic clades were robust (15 genera with minimal Bremer and bootstrap support 3 and 86%, respectively) but not further resolved at species levels. Inter-generic phylogenetic relationships were resolved only partly.

### 3.2. The contribution of different character types to clade support and speciation

**Figure 4.**
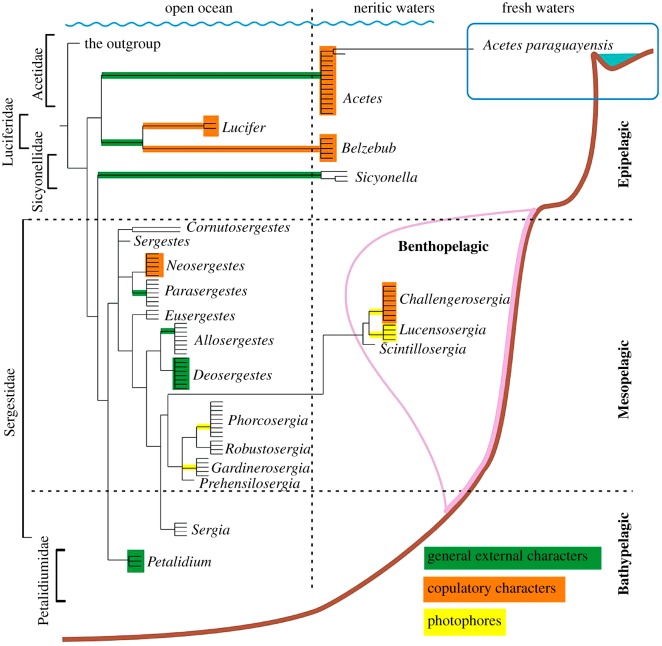
The distribution of positive Cs*_i_* and Cd*_i_* values for the family- and genus-level clades and occurrence of the supported clades in principal oceanic habitats according to [2–4,6,9–11]. The habitats are illustrated on the basis of the vertical distribution during the daytime. Cs_G_ and Cd_G_ (for general external characters) are in green, Cs_C_ and Cd_C_ (for copulatory characters) are in orange, Cs_Ph_ and Cd_Ph_ (for photophores) are in yellow.

If only general external characters were included in the matrix, the clades corresponding to Acetidae, Luciferidae, Sicyonellidae and Petalidiumidae were robust; all genus-level clades were supported except *Belzebub*. The Sergestidae and approximately half of the included genus-level clades were not resolved (electronic supplementary material, appendix S5). If all but general external characters were included, Petalidiumidae and Sergestidae with most included genus-level clades were robust (electronic supplementary material, appendix S5). Acetidae, Luciferidae and Sicyonellidae were not supported; both genera of Luciferidae were robust. The Cs_G_ values, indicating contribution of general external characters in the clade support, were positive for Acetidae, Luciferidae, Sicyonellidae and for two genera of Sergestidae: *Parasergestes* and *Allosergestes* (figure 4). The Cd_G_ metrics, showing the contribution of general external characters to speciation within the genera, were positive for *Deosergestes* and *Petalidium* (figure 4).

If only copulatory organ characters were included in the analysis, Petalidiumidae and both genera of Luciferidae were supported (electronic supplementary material, appendix S6). If all but copulatory characters were included, all the clades were robust except *Belzebub*, *Challengerosergia* and *Robustosergia* (electronic supplementary material, appendix S6). The Cs_C_ values were positive only for both genera of Luciferidae, while the Cd_C_ metrics were positive for *Acetes, Lucifer, Belzebub*, *Neosergestes* and *Challengerosergia* (figure 4).

If only photophores were included in the analysis, only four genera of Sergestidae were robust: *Challengerosergia, Lucensosergia*, *Gardinerosergia* and *Phorcosergia* (electronic supplementary material, appendix S7). Conversely, these clades were the only unsupported clades, if all characters but photophores are included (electronic supplementary material, appendix S7). The Cs_Ph_ values were positive for the same four genera of Sergestidae, while the Cd_Ph_ metric was positive for *Lucensosergia* only (figure 4).

### 3.3. The distribution of the clades in the main oceanic habitats

The retrieved clades Acetidae, Luciferidae, Sicyonellidae and part of Sergestidae (*Cornutosergestes*) are epipelagic [3,4,10,11], three generic clades within these clades (*Acetes*, *Belzebub* and *Sicyonella*) are neritic and one species of *Acetes* is even freshwater [10,11] (figure 4).

Most sergestids are mesopelagic oceanic interzonal species, which migrate diurnally (figure 4). Three genera, *Challengerosergia*, *Lucensosergia*, and *Scintillosergia* are benthopelagic and associated with continental slopes and seamounts [2–4] (figure 4).

Petalidiumidae and the genus *Sergia* of Sergestidae are bathypelagic [2,4,9] (figure 4).

## 4. Discussion

### 4.1. The major clades and a new classification of Sergestoidea

Many previously tested genus-level clades receive even better statistical support than before [4,9–11]. The present data show that the family Luciferidae is a natural group, whilst the remainder of the Sergestoidea, previously called the family Sergestidae, is not monophyletic and includes four basal monophyletic clades with good support: Acetidae, Sicyonellidae, Petalidiumidae and Sergestidae (figure 3).

Three new families of Sergestoidea are designated here (in addition to the previously established Luciferidae and Sergestidae): Acetidae, Sicyonellidae and Petalidiumidae. Their emended diagnoses, composition, and keys to genera and families are given in the electronic supplementary material, appendix S8. From the carcinological viewpoint, these families most remarkably differ in the morphology of the copulatory structures. Luciferidae have no clasping organ at all, Acetidae have two clasping tubercles (one may be reduced) and opposed claw-like setae, Sergestidae have a single clasping tubercle and opposed serrated bristles, Sicyonellidae have a row of serrated bristles and no tubercle, while Petalidiumidae have two opposed rows of serrated bristles (one of each replaces the tubercle). The general topology of the petasma is very characteristic for each family. The Luciferidae have a petasma without *pars astringens*, the *pars externa* is transformed into a sheath around *processus ventralis*. In the Acetidae, the petasma is often lacking a *pars astringens*, and the capitulum has no developed lobes and processes. The Sergestidae have all parts of the petasma developed and usually undivided. The Sicyonellidae (with the exception of *S. inermis*) and the Petalidiumidae have the most complex petasma with all parts present and a secondarily divided *processus ventralis*.

Other significant distinguishing characters include widely separated labrum and antennae and the absence of true chela in the Luciferidae; rudimentary chelae of the first to third pereopods and very reduced/absent natatory pereopods in the Acetidae; well-developed chelae on the first to third pereopods and a complete set of segments on the natatory pereopods in the Sicyonellidae; luminous organs (in 14 of 15 genera) in the Sergestidae.

### 4.2. The distribution of characters

External body characters are mostly homoplasious and have evolved independently: the armature of the carapace and grasping appendages, reduction of the natatory pereopods, etc. The membranous integument also independently evolved in such distant deep-sea clades as *Petalidium* and *Sergia*, probably as a convergent adaptation to the deep-sea life of these genera (figure 3).

The clasping copulatory organ also represents homoplasy: this organ independently evolved in Acetidae, Sicyonellidae, Petalidiumidae and Sergestidae and morphologically differs between these clades. On the other hand, the elaborated petasma is a synapomorphy of Sergestoidea; further evolution of this organ, including elaboration or loss of numerous lobes and processes, occurs in all clades and is linked to the synapomorphies supporting families and genera.

Such photophores as the organs of Pesta are homoplasious: they evolved independently in several genera and have different histological structures [3,4]. Conversely, dermal photophores evolved once in a single clade and were subsequently either lost (reversal in *Sergia*) or elaborated and possess lenses (homoplasies) in *Prehensilosergia, Scintillosergia, Lucensosergia* and *Challengerosergia*, probably as an adaptation to the benthopelagic habitat of these genera.

### 4.3. Contribution of different character types to the phylogeny of Sergestoidea

All character types have significantly contributed to the support of the revealed clades, as exclusion of any character type significantly impacts upon the resolution of the tree.

*General external characters* are the most important for support of the major (family-level) clades: four families remain supported if only this type is used in the analysis (electronic supplementary material, appendix S5). General external characters, however, are insufficient to support Sergestidae. Acetidae, Luciferidae and Sicyonellidae are especially sensitive to the absence of general external characters and collapse if this type is not included in the analysis.

General external characters significantly contribute to support of nearly half of the genera (electronic supplementary material, appendix S5). They are most important (positive Cs_G_) for *Parasergestes* and *Allosergestes,* in which synapomorphies are associated with the catching appendages (third maxilliped and second to third pereopods) and illustrate the offensive strategy of Sergestidae [4]. Further speciation within these genera (negative Cd_G_) is not dependent on this type of characters.

General external characters are significant in the speciation of *Petalidium* and *Deosergestes* and associated with development of branchiae (*Petalidium* occurs in waters with significantly different oxygen concentrations [4]) or catching appendages (*Deosergestes* illustrating offensive feeding strategy [4]).

*Copulatory characters* do not provide significant support for any of the five major clades. Only three genera, *Lucifer, Belzebub* and *Petalidium,* receive statistical support if only this type is included in the analysis (electronic supplementary material, appendix S6). The absence of copulatory characters in the analysis does not change the robustness of the tree significantly (electronic supplementary material, appendix S6). Positive Cs_C_ values confirm the contribution of copulatory characters in the support of *Lucifer* and *Belzebub*. Copulatory organ characters (positive Cd_C_) significantly contribute to speciation within *Lucifer, Belzebub, Neosergestes* and *Challengerosergia.* The copulatory characters appear not to be critical in the colonization of the major pelagic habitats; instead, they may promote sexual isolation during invasion of different parts of the epipelagic (oceanic *Lucifer* and neritic *Belzebub*) and mesopelagic (pelagic *Neosergestes* and benthopelagic *Challengerosergia*) biotopes.

*Photophores*, like copulatory characters, do not provide significant support of any family-level clades. Four genera, *Lucensosergia*, *Challengerosergia, Gardinerosergia* and *Phorcosergia,* receive statistical support if only this type is included in the analysis (electronic supplementary material, appendix S7). The absence of photophores in the dataset does not change the robustness of the consensus tree significantly (electronic supplementary material, appendix S7). Positive Cs_Ph_ and Cd_Ph_ values confirm the contribution of photophores to the support of the four genera above and to speciation within *Lucensosergia*.

### 4.4. The pathways into principal oceanic biotopes

The classical schema of vertical zonation of the pelagic ocean includes several vertical zones [15]. First is the epipelagic (0–200 m), in which there is enough sunlight during daytime to support primary production. Second is the mesopelagic zone, that receives enough solar illumination for the fauna to differentiate diurnal and nocturnal cycles, but not enough to support photosynthesis; in the plankton research, this zone is usually associated with the main pycnocline with the lower boundary normally ranging from 500 to 900 m [7,16]. Third is the bathypelagic zone below the main pycnocline, with relative invariance of light, temperature and salinity [15] and a lower boundary at a depth of 3000 m. These zones may be further divided horizontally. The epipelagic is traditionally divided into the oceanic zone and the neritic zone including shallow waters from the littoral to the edge of the continental shelf. The meso- and bathypelagic zones around continental slopes and seamounts are replaced adjacent to the sea-floor by the benthopelagic zone, where specific bottom-related fauna makes up over 50% of the total plankton biomass [17].

In the Sergestoidea, each major (family-level) clade occurs only within one of these main vertical zones (figure 4). Acetidae, Luciferidae and Sicyonellidae are epipelagic and the main synapomorphies supporting these clades are linked to spination/shape of body and to natatory appendages. Spination of the body is assumed to have a defensive function, while body shape and natatory appendages favour an escape function [4]; both are critical in the epipelagic where shelters are absent and carnivores are abundant [4].

The Sergestidae is mesopelagic and encompasses genera, which are supported by synapomorphies linked to luminous organs (the organ of Pesta or dermal photophores). The photophores tilt to maintain their downward orientation no matter which way the animal is swimming [18] and may thus provide ventral camouflage, the bioluminescent countershading that hides the silhouette from predators hunting from below [19]. The photophores in mesopelagic Sergestidae may also be a schooling aid: adults have well-developed photophores and form shoals [4] and their visual systems are tuned to detect bioluminescence [20,21], while earlier stages lack photophores, are scattered, and have different characteristics of their visual systems [20]. The evolution of the photophores may thus be critical for the invasion of the mesopelagic zone.

Petalidiumidae and *Sergia* (Sergestidae) are bathypelagic (figure 4). They have a membranous integument and fatty tissues and they lack photophores (not evolved in Petalidiumidae and lost in *Sergia*) that may illustrate possible adaptations to minimization of energy loss in areas depleted in food in the deep sea.

Some major (family-level) clades further diverge and colonize different habitats within the same vertical zone. The epipelagic Luciferidae is split into the oceanic *Lucifer* and the neritic *Belzebub.* The mesopelagic Sergestidae encompasses 11 oceanic and three benthopelagic genera (figure 4), which form a robust monophyletic clade *Challengerosergia* + *Lucensosergia* + *Scintillosergia.* The clade is supported by such a synapomorphy as thick lenses on dermal photophores, which may favour communication in the dim nepheloid layer associated with their benthopelagic habitat [17].

Exact geological timings for colonization of different biotopes are difficult to assess due to the absence of molecular clock data and poor fossilization of planktonic shrimps. Colonization of the meso- and bathypelagic most probably occurred after the end of the Mesozoic, when deep waters became well ventilated. High primary productivity followed this deep-ocean ventilation after the Eocene–Oligocene boundary [22,23] and favoured colonization of the deep sea. Recent studies have shown that deep-sea mesoplankton and shrimps respond to variations in surface productivity and their biomass in the meso- and even bathypelagic is correlated with the averaged surface chlorophyll [24,25]. Thus, colonization of the deep sea could be promoted by both oxygenization and an increased flux of organic matter from the euphotic zone. This is supported by the findings that the end of the Mesozoic was important for colonization of the pelagic by other groups such as shallow-living cetaceans [26,27] and deep-sea copepods [28].

Colonization of the epipelagic could be influenced by climate change more directly. For example, climate change after the middle of the Eocene in the American side of the Atlantic could cause immigration of tropical and subtropical species of *Acetes* to equatorial latitudes and force the equatorial species to take refuge in fresh waters [29].

**Figure 5.**
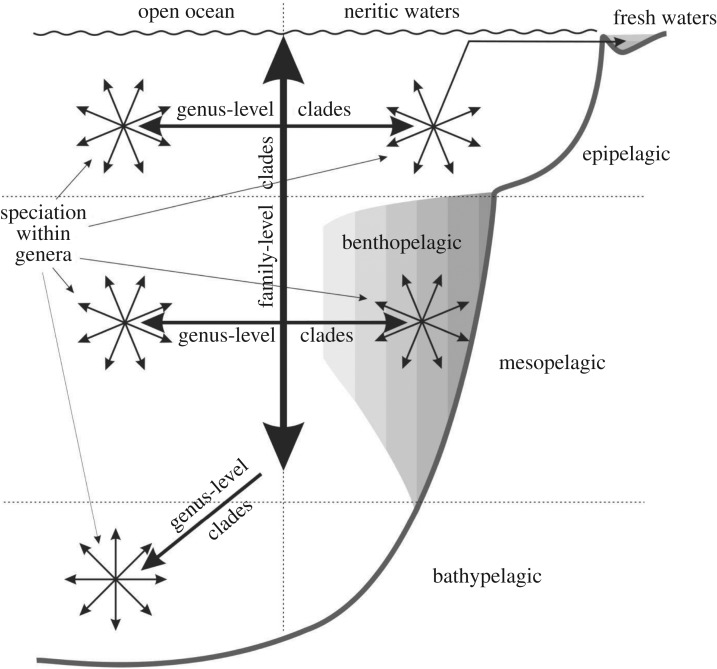
Colonization of the principal oceanic habitats by family- and genus-level clades of Sergestoidea with further speciation within colonized biotopes.

In conclusion, I propose an evolutionary model of Sergestoidea showing their pathways into their principal biotopes (figure 5). The basal (family-level) clades appear to have diverged vertically, colonizing the water column from the surface to a depth of nearly 3000 m. Furthermore, each of these clades evolved within the same vertical range; the divergence at the genus level, if any, occurred only within a vertical zone in horizontal directions, with the invasion of the benthopelagic and neritic (shelf and estuarine) habitats. The speciation within the genera occurred mainly within vertical and horizontal habitats (figure 5), mostly sympatrically [4]. Greatly elaborated copulatory morphology (clasping organ and petasma) could favour sexual isolation between sympatric species.

## Supplementary Material

Appendix 1

## Supplementary Material

Appendix 2

## Supplementary Material

Appendix 3

## Supplementary Material

Appendix 4

## Supplementary Material

Appendix 5.

## Supplementary Material

Appendix 6

## Supplementary Material

Appendix 7

## Supplementary Material

Appendix 8

## Data accessibility

Our data are deposited at http://dx.doi.org/10.5061/dryad.c8f7r [30].
