# Supplementary material for: The shrimp superfamily Sergestoidea: a global phylogeny with definition of new families and an assessment of the pathways into principal biotopes
Source: R Soc Open Sci. 2017 Sep 6;4(9):170221. doi: 10.1098/rsos.170221 (PMC5627073; doi:10.1098/rsos.170221)
Supplement: Appendix 1 [file rsos170221supp1.doc]

Appendix 1. List of examined specimens.

BM - Bergen Museum, Norway; DTR - Department of Tropical Research, New York Zoological Society, USA; MBC - Marine Biological Center, Tokai University, Japan; NHM - British Museum (Natural History); BMNH - Museum National d’Histoire Naturelle, France; SMNH - Naturhistoriska Riksmuseet, Sweden; SMNH - United States National Museum; YPM - Yale Peabody Museum, USA; ZMUC - Zoological Museum, University of Copenhagen, Denmark.

|  | **Species** | **Catalogue number** |
| --- | --- | --- |
| 1 | *Acetes carolina* Hansen, 1933 | ZMUC CRU-03465б 03106, 04452 (cotype) |
| 2 | *Acetes chinensis* Hansen, 1919 | ZMUC CRU-04453, 03724, 03725 |
| 3 | *Acetes erythraeus* Nobili, 1905 | ZMUC CRU-04432, 04433 |
| 4 | *Acetes indicus* H. Milne Edwards, 1830 | ZMUC CRU-04441, 04442 |
| 5 | *Acetes intermedius* Omori, 1975 | ZMUC CRU-04423, 04424 |
| 6 | *Acetes japonicus* [Kishinouye, 1905](http://marinespecies.org/aphia.php?p=taxdetails&id=210646) | ZMUC CRU-04438, 04439 |
| 7 | *Acetes marinus* [Omori, 1975](http://marinespecies.org/aphia.php?p=taxdetails&id=241255) | ZMUC CRU-04420, 04421 |
| 8 | *Acetes paraguayensis* [Hansen, 1919](http://marinespecies.org/aphia.php?p=taxdetails&id=241256) | ZMUC CRU-04426, 04427 |
| 9 | *Acetes petrunkevitchi* (Burkenroad, 1945) | SMNH Type 2338 (holotype) |
| 10 | *Acetes serrulatus* [(Krøyer, 1859)](http://marinespecies.org/aphia.php?p=taxdetails&id=377411) | ZMUC CRU-04435, 04436 |
| 11 | *Acetes sibogae* [Hansen, 1919](http://marinespecies.org/aphia.php?p=taxdetails&id=210650) | ZMUC CRU-04429, 04430 |
| 12 | *Acetes vulgaris* [Hansen, 1919](http://marinespecies.org/aphia.php?p=taxdetails&id=377412) | ZMUC CRU-03962, 04400 |
| 13 | *Allosergestes index* (Burkenroad, 1940) | ZMUC CRU–1621 (holotype) |
| 14 | *Allosergestes nudus* (Illig, 1914) | MNB, No 17581(holotype); ZMUC CRU–4514. |
| 15 | *Allosergestes oleseni* (Vereshchaka, 2009) | ZMUC CRU–4840 (holotype) |
| 16 | *Allosergestes pectinatus* (Sund, 1920) | BM No 13684, 13686, 13683, 13685, 13683. ZMUC CRU–4519. |
| 17 | *Allosergestes pestafer* (Burkenroad, 1937) | DTR 361.031 (holotype); ZMUC CRU–4539. |
| 18 | *Allosergestes sargassi* (Ortmann, 1893) | ZMUC CRU–4548, 04548. |
| 19 | *Allosergestes verpus* (Burkenroad, 1940) | ZMUC CRU–1620 (holotype); 4517 |
| 20 | *Allosergestes vinogradovi* (Vereshchaka, 2009) | ZMUC CRU–4838 (holotype), 4839. |
| 21 | *Belzebub chacei* (Bowman, 1967) | ZMUC CRU-4440 |
| 22 | *Belzebub faxoni* (Borradaile, 1915) | ZMUC CRU 4448, ZMUC CRU-4450 |
| 23 | *Belzebub hanseni* (Nobili, 1905) | ZMUC CRU-4428 |
| 24 | *Belzebub intermedius* (Hansen, 1919) | ZMUC CRU-4422 |
| 25 | *Belzebub репicillifer* (Hansen, 1919) | ZMUC CRU-4431 |
| 26 | *Challengerosergia challengeri* (Hansen, 1903) | BMNH 1903.6.6.14 (holotype) |
| 27 | *Challengerosergia hansjacobi* (Vereshchaka, 1994) | ZMUC CRU-3613 (holotype) |
| 28 | *Challengerosergia jeppeseni* (Vereshchaka, 2000) | ZMUC CRU-361 (holotype) |
| 29 | *Challengerosergia oksanae* (Vereshchaka, 2000) | ZMUC CRU-3615 (holotype) |
| 30 | *Challengerosergia stellata* (Burkenroad, 1940) | ZMUC CRU-1607 (holotype) |
| 31 | *Challengerosergia talismani* (Barnard, 1947) | MNHN Na 351 |
| 32 | *Cornutosergestes cornutus* (Krøyer, 1855) | ZMUC CRU–4533 (syntypes) |
| 33 | *Cornutosergestes mepae* (Vereshchaka, 2009) | ZMUC CRU–4836, 4837 (holotype) |
| 34 | *Deosergestes coalitus* (Burkenroad, 1940) | ZMUC CRU–1619, 4508, 4532, 4546, 4537, 4516, 4531, 4531, 4537, 4516 |
| 35 | *Deosergestes corniculum* (Krøyer, 1855) | ZMUC CRU-6077 (syntypes), 4520; NHM, 88.22; MNHN MP NA12793. |
| 36 | *Deosergestes disjunctus* (Burkenroad, 1940) | ZMUC CRU-1618 (holotype), 4535. |
| 37 | *Deosergestes henseni* (Ortmann, 1893) | ZMUC CRU–4505 |
| 38 | *Deosergestes paraseminudus* (Crosnier & Forest, 1973) | MNHN NA 10173 (holotype), MNHN NA 10176 (paratype); ZMUC CRU-4521. |
| 39 | *Deosergestes pediformis* (Crosnier & Forest, 1973) | MNHN NA 10099 (holotype); MNHN NA 10110; ZMUC CRU–4540. |
| 40 | *Deosergestes rubroguttatus* (Wood-Mason in Wood-Mason & Alcock, 1891) | ZMUC CRU–4518 |
| 41 | *Deosergestes seminudus* (Hansen, 1919) | ZMUC CRU–8051, 4506. |
| 42 | *Eusergestes antarcticus* (Vereshchaka, 2009) | ZMUC CRU–4834 (holotype); 4835 |
| 43 | *Eusergestes arcticus* (Krøyer, 1855) | ZMUC CRU–05590 (holotype), ZMUC CRU-04528, 7960, 4843 |
| 44 | *Eusergestes similis* (Hansen, 1903) | NHM, 1903.6.6.15 (holotype). ZMUC CRU–4833. |
| 45 | *Gardinerosergia bigemmea* (Burkenroad, 1940) | ZMUC CRU-1600 |
| 46 | *Gardinerosergia gardneri* (Kemp, 1913) | ZMUC CRU-03726 |
| 47 | *Gardinerosergia kensleyi* (Vereshchaka, 2000) | ZMUC CRU-3605 (holotype), 3606, 3619 |
| 48 | *Gennadas parvus* Bate, 1881 | ZMUC CRU-04419 |
| 49 | *Lucensosergia crosnieri* (Vereshchaka, 2000) | ZMUC CRU-3617 (holotype) |
| 50 | *Lucensosergia lucens* (Hansen, 1922) | ZMUC CRU-04425 |
| 51 | *Lucifer orientalis* Hansen, 1919 | ZMUC CRU-4437 |
| 52 | *Lucifer typus* H. Milne Edwards, 1837 | ZMUC CRU-4447 |
| 53 | *Neosergestes brevispinatus* (Judkins, 1978) | USNM 155056 (holotype), 4538; USNM 155057 |
| 54 | *Neosergestes consobrinus* (Milne, 1968) | USNM 112949 (holotype), 112948; ZMUC CRU–4550 |
| 55 | *Neosergestes edwardsi* (Krøyer, 1855) | ZMUC CRU–5879, 7619, 4526 04526. |
| 56 | *Neosergestes orientalis* (Hansen, 1919) | USNM 155060, 155062, 155066; ZMUC CRU–4511. |
| 57 | *Neosergestes semissis* (Burkenroad, 1940) | ZMUC CRU–1624 (holotype); 4509 |
| 58 | *Neosergestes tantillus* (Burkenroad, 1940) | ZMUC CRU–1623 (holotype) |
| 59 | *Parasergestes armatus* (Krøyer, 1855) | ZMUC CRU–5626 (holotype), 6770, 4507. MBC No IORDIN 79–79, 79–80. |
| 60 | *Parasergestes cylindricus* (Vereshchaka, 2009) | ZMUC CRU–4527 (holotype) |
| 61 | *Parasergestes diapontius* (Bate, 1881) | NHM, No 88.22 (holotype); ZMUC CRU–4530 |
| 62 | *Parasergestes halia* (Faxon, 1893) | ZMUC CRU–4534 |
| 63 | *Parasergestes sirenkoi* (Vereshchaka, 2009) | ZMUC CRU–4841 (holotype), 4842 |
| 64 | *Parasergestes stimulator* (Burkenroad, 1940) | ZMUC CRU–1622 (holotype) |
| 65 | *Parasergestes vigilax* (Stimpson, 1860*)* | ZMUC CRU–4510 |
| 66 | *Penaeus monodon* Fabricius, 1798 | ZMUC CRU-004445 |
| 67 | *Petalidium foliaceum* Bate, 1881 | NHM 1888.22, 1888.22, 1903.6.6.16 (syntypes, damaged), ZMUC CRU-20546 |
| 68 | *Petalidium obesum* (Krøyer, 1859) | ZMUC CRU-007582 (holotype) |
| 69 | *Petalidium suspiriosum* Burkenroad, 1937 | YPM IZ 039178, 068756 |
| 70 | *Phorcosergia burukovskii* (Vereshchaka, 2000) | ZMUC CRU-3607 (holotype), 3608, 3609 |
| 71 | *Phorcosergia filicta* (Burkenroad, 1940) | ZMUC CRU-1603 (holotype) |
| 72 | *Phorcosergia phorca* (Faxon, 1893) | ZMUC CRU-04434 |
| 73 | *Phorcosergia wolffi* (Vereshchaka, 1994) | ZMUC CRU-1612 (holotype), 1613 (paratype) |
| 74 | *Prehensilosergia prehensilis* (Bate, 1881) | BMNH1888.2 (holotype) |
| 75 | *Robustosergia regalis* (Gordon, 1939) | ZMUC CRU-1601 |
| 76 | *Robustosergia robusta* (Smith, 1882) | USNM 7316 |
| 77 | *Robustosergia vityazi* (Vereshchaka, 2000) | ZMUC CRU3610 (holotype), 3611, 3612 |
| 78 | *Scintillosergia scintillans* (Burkenroad, 1940) | ZMUC CRU-03613 |
| 79 | *Sergestes atlanticus* Milne-Edwards, 1830 | MNHN, NA 331 (syntypes) 13574, ZMUC CRU-04542, 6470 |
| 80 | *Sergia inoa* (Faxon, 1893) | MCZ 4666 (holotype) |
| 81 | *Sergia laminata* (Burkenroad, 1940) | ZMUC CRU-1605, MNHN-Na 10150, MNHN-Na 4369 |
| 82 | *Sergia remipes* Stimpson, 1860 | USNM 7106, BMNH1888.22 |
| 83 | *Sergia tenuiremis* (Krøyer, 1855) | ZMUC CRU-08362 |
| 84 | *Sicyonella antennata* [Hansen, 1919](http://marinespecies.org/aphia.php?p=taxdetails&id=377412) | ZMUC ALV-1 |
| 85 | *Sicyonella inermis* (Paulson, 1875) | USNM 1026370 |
| 86 | *Sicyonella maldivensis* Borradaile, 1910 | ZMUC CRU-04443 |

Appendix 2. Changes in the synonymy of Sergestoidea not indicated in [30] or made later on.

| No | Old name | New name | Source |
| --- | --- | --- | --- |
| 1 | *Deosergestes curvatus* (Crosnier & Forest, 1973) | *Deosergestes corniculum* (Krøyer, 1855) | [3]^1^ |
| 2 | *Deosergestes erectus* (Burkenroad, 1940) | *Deosergestes coalitus* (Burkenroad, 1940) | [3]^1^ |
| 3 | *Deosergestes nipponensis* (Yokoya, 1933) | *Deosergestes seminudus* (Hansen, 1919) | [3]^1^ |
| 4 | *Lucifer* Thompson, 1829 [part] | *Belzebub* Vereshchaka, Olesen & Lunina, 2016 | [11] |
| 5 | *Lucifer chacei* Bowman, 1967 | *Belzebub* *chacei* Vereshchaka, Olesen & Lunina, 2016 | [11] |
| 6 | *Lucifer faxoni* Borradaile, 1915 | *Belzebub* *faxoni* Vereshchaka, Olesen & Lunina, 2016 | [11] |
| 7 | *Lucifer hanseni* Nobili, 1905 | *Belzebub* *hanseni* Vereshchaka, Olesen & Lunina, 2016 | [11] |
| 8 | *Lucifer intermedius* Hansen, 1919 | *Belzebub* *intermedius* Vereshchaka, Olesen & Lunina, 2016 | [11] |
| 9 | *Lucifer penicillifer* Hansen, 1919 | *Belzebub* *penicillifer* Vereshchaka, Olesen & Lunina, 2016 | [11] |
| 10 | *Neosergestes geminus* (Judkins, 1978) | *Neosergestes orientalis* (Hansen, 1919) | [3]^1^ |
| 11 | *Neosergestes gibbilobatus* (Judkins, 1978) | *Neosergestes orientalis* (Hansen, 1919) | [3]^1^ |
| 12 | *Parasergestes extensus* (Hanamura, 1983) | *Parasergestes armatus* (Krøyer, 1855) | [3]^1^ |
| 13 | *Peisos* Burkenroad, 1945 | *Acetes* H. Milne Edwards, 1830 | [10] |
| 14 | *Peisos petrunkevitchi* Burkenroad, 1945 | *Acetes petrunkevitchi* (Burkenroad, 1945) | [10] |
| 16 | *Sergestes cornutus* Krøyer, 1855 | *Cornutosergestes cornutus* (Krøyer, 1855) | [4] |
| 17 | *Sergestes hamifer* Alcock & Anderson, 1894 | *Parasergestes armatus* (Krøyer, 1855) | **New synonym**, based upon original description and fugures |
| 19 | *Sergia erythraeensis* Iwasaki & Couwelaar, 2001 | *Lucensosergia colosii* (Cecchini, 1933) | **New synonym**, based upon original description and fugures |
| 20 | *Sergia manningorum* Froglia & Gramitto | *Robustosergia robusta* (Smith, 1882) | **New synonym**, based upon original description and fugures |
| 21 | *Sergia japonica* (Bate, 1881) | *Sergia remipes* Stimpson, 1860 | **New synonym**, based upon original description |

^1^ - first synonymy included this species in the genus *Sergestes* H. Milne Edwards, 1830.

Appendix 3. List of characters used.

| Charac-ter No | Character state | State No | Reference to figure and source |
| --- | --- | --- | --- |
| **CARAPACE** | | | |
| 0 | Integument firm | 0 |  |
|  | Integument membranous^1^ | 1 |  |
| 1 | Labrum not much separated from antennae and eyes | 0 |  |
|  | Labrum widely separated from antennae and eyes | 1 |  |
| 2 | Rostrum bears 2or more dorsal teeth behind the orbital margin | 0 |  |
|  | Rostrum bears 0-1 dorsal teeth behind the orbital margin^2^ | 1 |  |
| 3 | Frontal margin of rostrum oblique | 0 | 2B,D – Vereshchaka *et al*. (2014) |
|  | Frontal margin of rostrum vertical | 1 | 2A – Vereshchaka *et al*. (2014) |
| 4 | Supraorbital tooth absent | 0 | 2A-C – Vereshchaka *et al*. (2014) |
|  | Supraorbital tooth present | 1 | 2D – Vereshchaka *et al*. (2014) |
| 5 | Pterygostomial tooth absent | 0 |  |
|  | Pterygostomial tooth present | 1 |  |
| 6 | Hepatic protrusion prominent | 0 |  |
|  | Hepatic protrusion inconspicuous | 1 |  |
| 7 | Hepatic spine absent | 0 | 2A-C – Vereshchaka *et al*. (2014) |
|  | Hepatic present | 1 | 2D – Vereshchaka *et al*. (2014) |
| 8 | Hepatic barb absent | 0 | 2A-C – Vereshchaka *et al*. (2014) |
|  | Hepatic barb present | 1 | 2D – Vereshchaka *et al*. (2014) |
| **BRANCHS** | | | |
| 9 | Somite VIII, arthrobranch developed | 0 |  |
|  | Somite VIII, arthrobranch rudimentary or absent^2^ | 1 |  |
| 10 | Somite VIII, arthrobranch absent | 0 |  |
|  | Somite VIII, arthrobranch present | 1 |  |
| 11 | Somite IX, anterior arthrobranch present | 0 |  |
|  | Somite IX, anterior arthrobranch absent | 1 |  |
| 12 | Somite IX, posterior arthrobranch present | 0 |  |
|  | Somite IX, posterior arthrobranch absent | 1 |  |
| 13 | Somite IX, posterior arthrobranch developed | 0 |  |
|  | Somite IX, posterior arthrobranch reduced | 1 |  |
| 14 | Somite IX, posterior arthrobranch dendritic | 0 |  |
|  | Somite IX, posterior arthrobranch lamellar | 1 |  |
| 15 | Somite X, anterior arthrobranch present | 0 |  |
|  | Somite X, anterior arthrobranch absent | 1 |  |
| 16 | Somite X, posterior arthrobranch present | 0 |  |
|  | Somite X, posterior arthrobranch absent | 1 |  |
| 17 | Somite X, posterior arthrobranch developed | 0 |  |
|  | Somite X, posterior arthrobranch reduced | 1 |  |
| 18 | Somite X, posterior arthrobranch dendritic | 0 |  |
|  | Somite X, posterior arthrobranch lamellar | 1 |  |
| 19 | Somite XI, anterior arthrobranch present | 0 |  |
|  | Somite XI, anterior arthrobranch absent | 1 |  |
| 20 | Somite XI, posterior arthrobranch present | 0 |  |
|  | Somite XI, posterior arthrobranch absent | 1 |  |
| 21 | Somite XI, posterior arthrobranch developed | 0 |  |
|  | Somite XI, posterior arthrobranch reduced | 1 |  |
| 22 | Somite XI, posterior arthrobranch dendritic | 0 |  |
|  | Somite XI, posterior arthrobranch lamellar | 1 |  |
| 23 | Somite XII, anterior arthrobranch present | 0 |  |
|  | Somite XII, anterior arthrobranch absent | 1 |  |
| 24 | Somite XII, developed anterior arthrobranch present | 0 |  |
|  | Somite XII, developed anterior arthrobranch absent | 1 |  |
| 25 | Somite XII, rudimentary anterior arthrobranch present | 0 |  |
|  | Somite XII, rudimentary anterior arthrobranch absent | 1 |  |
| 26 | Somite XII, posterior arthrobranch present | 0 |  |
|  | Somite XII, posterior arthrobranch absent | 1 |  |
| 27 | Somite XII, reduced posterior arthrobranch present | 0 |  |
|  | Somite XII, reduced posterior arthrobranch absent | 1 |  |
| 28 | Somite XII, rudimentary dendritic posterior arthrobranch present | 0 |  |
|  | Somite XII, rudimentary dendritic posterior arthrobranch absent | 1 |  |
| 29 | Somite XII, rudimentary lamellar posterior arthrobranch present | 0 |  |
|  | Somite XII, rudimentary lamellar posterior arthrobranch absent | 1 |  |
| 30 | Somite XIII, anterior arthrobranch present | 0 |  |
|  | Somite XIII, anterior arthrobranch absent | 1 |  |
| 31 | Somite XIII, posterior arthrobranch present | 0 |  |
|  | Somite XIII, posterior arthrobranch absent | 1 |  |
| 32 | Somite XIII, posterior arthrobranch developed | 0 |  |
|  | Somite XIII, posterior arthrobranch reduced | 1 |  |
| 33 | Somite XIII, posterior arthrobranch dendritic | 0 |  |
|  | Somite XIII, posterior arthrobranch lamellar | 1 |  |
| **ABDOMEN AND TELSON** | | | |
| 34 | Somite VI in male without ventral processes | 0 | 1 – Vereshchaka *et al*. (2016) |
|  | Somite VI in male with two ventral processes | 1 | 1 – Vereshchaka *et al*. (2016) |
| 35 | Somite VI in male without not tapering, obtuse posterior ventral process | 0 | 1 – Vereshchaka *et al*. (2016) |
|  | Somite VI in male with not tapering, obtuse posterior ventral process | 1 | 1 – Vereshchaka *et al*. (2016) |
| 36 | Somite VI in male without tapering, acute posterior ventral process^3^ | 0 | 1 – Vereshchaka *et al*. (2016) |
|  | Somite VI in male with tapering, acute posterior ventral process | 1 | 1 – Vereshchaka *et al*. (2016) |
| 37 | Somite VI in male without straight posterior ventral process^3^ | 0 | 1 – Vereshchaka *et al*. (2016) |
|  | Somite VI in male with straight posterior ventral process | 1 | 1 – Vereshchaka *et al*. (2016) |
| 38 | Somite VI in male without curved posterior ventral process^3^ | 0 | 1 – Vereshchaka *et al*. (2016) |
|  | Somite VI in male with curved posterior ventral process | 1 | 1 – Vereshchaka *et al*. (2016) |
| 39 | Telson, lateral spines present | 0 |  |
|  | Telson, lateral spines absent | 1 |  |
| 40 | Telson without four movable lateral spines | 0 |  |
|  | Telson with four movable lateral spines | 1 |  |
| 41 | Telson without two movable lateral spines | 0 |  |
|  | Telson with two movable lateral spines | 1 |  |
| 42 | Telson in male straight distoventrally | 0 | 1 – Vereshchaka *et al*. (2016) |
|  | Telson in male with conspicuous distoventral protuberance | 1 | 1 – Vereshchaka *et al*. (2016) |
| **EYE** | | | |
| 43 | Eyes not sexually dimorphic | 0 |  |
|  | Eyes sexually dimorphic, larger in males | 1 |  |
| 44 | Eyestalks respectively short, not reaching end of scaphocerite | 0 |  |
|  | Eyestalks elongated, nearly reaching end of scaphocerite | 1 |  |
| **ANTENNULE** | | | |
| 45 | First segment respectively short (less than by half or more longer than 3rd segment) | 0 |  |
|  | First segment elongated (by half or more longer than 3rd segment) | 1 | 2E – Vereshchaka *et al*. (2014) |
| 46 | Third segment respectively short (less than by half or more longer than 3rd segment) | 0 |  |
|  | Third segment elongated (by half or more longer than 3rd segment) | 1 | 2F – Vereshchaka *et al*. (2014) |
| 47 | Third segment distoventrally unarmed in male | 0 |  |
|  | Third segment with distoventral processus in male | 1 | 2G – Vereshchaka *et al*. (2014) |
| 48 | Antennules in male with ventral flagellum | 0 |  |
|  | Antennules in male without ventral flagellum | 1 |  |
| 49 | Mobile stylocerite absent | 0 |  |
|  | Mobile stylocerite present | 1 |  |
| 50 | Fixed stylocerite absent | 0 |  |
|  | Fixed stylocerite present | 1 |  |
| **ANTENNA** | | | |
| 51 | Distal tooth of scaphocerite not reaching distal end of blade | 0 |  |
|  | Distal tooth of scaphocerite reaching distal end of blade | 1 | 7G – Vereshchaka *et al*. (2014) |
|  | Distal tooth of scaphocerite overreaching distal end of blade | 2 | 7E – Vereshchaka *et al*. (2014) |
| **MANDIBLE** | | | |
| 52 | Mandibular palp present | 0 |  |
|  | Mandibular palp absent | 1 |  |
| **MAXILLULE** | | | |
| 53 | Four endites absent in adults | 0 |  |
|  | Four endites present in adults | 1 |  |
| 54 | Three endites absent in adults | 0 |  |
|  | Three endites present in adults | 1 |  |
| 55 | Two endites absent in adults | 0 |  |
|  | Two endites present in adults | 1 |  |
| 56 | A single endite absent in adults | 0 |  |
|  | A single endite present in adults | 1 |  |
| **FIRST MAXILLIPED** | | | |
| 57 | Epipod present | 0 |  |
|  | Epipod absent | 1 |  |
| 58 | Exopod present | 0 |  |
|  | Exopod absent | 1 |  |
| 59 | Endopod well-developed | 0 |  |
|  | Endopod rudimentary/absent | 1 |  |
| 60 | Reduced 2-segmented endopod absent | 0 |  |
|  | Reduced 2-segmented endopod present | 1 |  |
| **SECOND MAXILLIPED** | | | |
| 61 | Epipod present | 0 |  |
|  | Epipod absent | 1 |  |
| **THIRD MAXILLIPED** | | | |
| 62 | Moderately developed, < 2.0 times as long as first pereopod | 0 | 2E – Vereshchaka *et al*. (2014) |
|  | Enlarged, > 2.0 times as long as first pereopod | 1 | 2F – Vereshchaka *et al*. (2014) |
| 63 | Not sexually dimorphic, dactyl not modified | 0 | 3D – Vereshchaka *et al*. (2014) |
|  | Sexually dimorphic, dactyl with oval subsegments in males | 1 | 3E – Vereshchaka *et al*. (2014) |
| 64 | Dactyl entire | 0 |  |
|  | Dactyl subdivided | 1 |  |
| 65 | Dactyl subdivided into ordinary subsegments absent | 0 |  |
|  | Dactyl subdivided into ordinary subsegments present | 1 | 2E – Vereshchaka *et al*. (2014) |
| 66 | Dactyl subdivided into specialized subsegments absent | 0 |  |
|  | Dactyl subdivided into specialized subsegments present | 1 | 2F – Vereshchaka *et al*. (2014) |
| 67 | Dactyl with four specialized subsegments absent | 0 |  |
|  | Dactyl with four specialized subsegments present | 1 | 6 – Vereshchaka (2009) |
| 68 | Dactyl with five specialized subsegments absent | 0 |  |
|  | Dactyl with five specialized subsegments present | 1 | 6 – Vereshchaka (2009) |
| 69 | Dactyl with six specialized subsegments absent | 0 |  |
|  | Dactyl with six specialized subsegments present | 1 | 6 – Vereshchaka (2009) |
| 70 | Dactyl with seven specialized subsegments absent | 0 |  |
|  | Dactyl with seven specialized subsegments present | 1 | 6 – Vereshchaka (2009) |
| **FIRST PEREOPOD** | | | |
| 71 | Ischium nearly smooth | 0 |  |
|  | Ischium with strong movable spines | 1 | 3F – Vereshchaka *et al*. (2014) |
| 72 | Chela absent | 0 |  |
|  | Chela present | 1 |  |
| 73 | Normal chela (palm nearly as long as fingers) absent | 0 |  |
|  | Normal chela (palm nearly as long as fingers) present | 1 |  |
| 74 | Much reduced chela ( palm >10 times as long as fingers) absent | 0 |  |
|  | Much reduced chela ( palm >10 times as long as fingers) present | 1 |  |
| **SECOND PEREOPOD** | | | |
| 75 | Ischium nearly smooth | 0 |  |
|  | Ischium with strong distally curved tooth | 1 | 3G – Vereshchaka *et al*. (2014) |
| 76 | Merus distally unarmed | 0 |  |
|  | Merus with distal protrusion | 1 | 3G – Vereshchaka *et al*. (2014) |
| 77 | Chela present | 0 |  |
|  | Chela absent | 1 |  |
| 78 | Slightly reduced chela (palm twice as long as fingers) absent | 0 |  |
|  | Slightly reduced chela (palm twice as long as fingers) present | 1 |  |
| 79 | Much reduced chela ( palm >10 times as long as fingers) absent | 0 |  |
|  | Much reduced chela ( palm >10 times as long as fingers) present | 1 |  |
| 80 | Chela without rudimentary fixed finger, which is shorter then dactyl | 0 |  |
|  | Chela with rudimentary fixed finger, which is shorter then dactyl | 1 | 3H – Vereshchaka *et al*. (2014) |
| 81 | Chela without well-developed fixed finger, which is as long as then dactyl | 0 |  |
|  | Chela with well-developed fixed finger, which is as long as then dactyl | 1 | 3I – Vereshchaka *et al*. (2014) |
| 82 | Chela with short setae not overreaching setae in tufts | 0 |  |
|  | Chela with very long setae overreaching setae in tufts | 1 | 3I – Vereshchaka *et al*. (2014) |
| **THIRD PEREOPOD** | | | |
| 83 | Coxa, mesial tooth absent | 0 |  |
|  | Coxa, mesial tooth present | 1 |  |
| 84 | Basis in female rounded | 0 |  |
|  | Basis in female with small projection or tooth | 1 |  |
| 85 | Propodus without specialized strong curved spines proximal to tufts of setae | 0 |  |
|  | Propodus with specialized strong curved spines proximal to tufts of setae | 1 | 3J – Vereshchaka *et al*. (2014) |
| 86 | True chela | 0 |  |
|  | Subchela | 1 | 3I – Vereshchaka *et al*. (2014) |
| 87 | Slightly reduced chela (palm 3 times as long as fingers) absent | 0 |  |
|  | Slightly reduced chela (palm 3 times as long as fingers) present | 1 |  |
| 88 | Much reduced chela ( palm >10 times as long as fingers) absent | 0 |  |
|  | Much reduced chela ( palm >10 times as long as fingers) present | 1 |  |
| 89 | Chela without rudimentary fixed finger, which is shorter then dactyl | 0 |  |
|  | Chela with rudimentary fixed finger, which is shorter then dactyl | 1 | 3J – Vereshchaka *et al*. (2014) |
| 90 | Chela without well-developed fixed finger, which is as long as then dactyl | 0 |  |
|  | Chela with well-developed fixed finger, which is as long as then dactyl | 1 | 3K– Vereshchaka *et al*. (2014) |
| 91 | Chela without very long setae overreaching setae in tufts | 0 |  |
|  | Chela with very long setae overreaching setae in tufts | 1 | 3J – Vereshchaka *et al*. (2014) |
| **FOURTH PEREOPOD** | | | |
| 92 | Present in female | 0 |  |
|  | Absent in female | 1 |  |
| 93 | Female without 7-segmented fourth pereopod | 0 |  |
|  | Female with 7-segmented fourth pereopod | 1 |  |
| 94 | Female without 6-segmented fourth pereopod | 0 |  |
|  | Female with 6-segmented fourth pereopod | 1 |  |
| 95 | Female without 5-segmented fourth pereopod | 0 |  |
|  | Female with 5-segmented fourth pereopod | 1 |  |
| 96 | Present in male | 0 |  |
|  | Absent in male | 1 |  |
| 97 | Male without 7-segmented fourth pereopod | 0 |  |
|  | Male with 7-segmented fourth pereopod | 1 |  |
| 98 | Male without 6-segmented fourth pereopod | 0 |  |
|  | Male with 6-segmented fourth pereopod | 1 |  |
| 99 | Male without 3-segmented fourth pereopod | 0 |  |
|  | Male with 3-segmented fourth pereopod | 1 |  |
| 100 | Carpus and propodus setose along one margin absent | 0 |  |
|  | Carpus and propodus setose along one margin present | 1 |  |
| 101 | Carpus and propodus setose along both margins absent | 0 |  |
|  | Carpus and propodus setose along both margins present | 1 |  |
| **FIFTH PEREOPOD** | | | |
| 102 | Present in female | 0 |  |
|  | Absent in female | 1 |  |
| 103 | Female without 7-segmented fourth pereopod | 0 |  |
|  | Female with 7-segmented fourth pereopod | 1 |  |
| 104 | Female without 6-segmented fourth pereopod | 0 |  |
|  | Female with 6-segmented fourth pereopod | 1 |  |
| 105 | Female without 3-segmented fourth pereopod | 0 |  |
|  | Female with 3-segmented fourth pereopod | 1 |  |
| 106 | Present in male | 0 |  |
|  | Absent in male | 1 |  |
| 107 | Male without 7-segmented fourth pereopod | 0 |  |
|  | Male with 7-segmented fourth pereopod | 1 |  |
| 108 | Male without 6-segmented fourth pereopod | 0 |  |
|  | Male with 6-segmented fourth pereopod | 1 |  |
| 109 | Male without 1-segmented fourth pereopod | 0 |  |
|  | Male with 1-segmented fourth pereopod | 1 |  |
| 110 | Carpus and propodus setose along one margin absent | 0 |  |
|  | Carpus and propodus setose along one margin present | 1 |  |
| 111 | Carpus and propodus setose along both margins absent | 0 |  |
|  | Carpus and propodus setose along both margins present | 1 |  |
| **UROPODAL EXOPOD** | | | |
| 112 | Outer spine absent | 0 | 10 – Vereshchaka (2009) |
|  | Outer spine present | 1 | 10 – Vereshchaka (2009) |
| 113 | Outer margin of proximal segment naked | 0 | 10 – Vereshchaka (2009) |
|  | Outer margin of proximal segment setose | 1 | 10 – Vereshchaka (2009) |
| 114 | Outer margin of proximal segment not setose or setose entirely | 0 |  |
|  | Outer margin of proximal segment setose partly | 1 | 10 – Vereshchaka (2009) |
| 115 | Outer margin of proximal segment not setose or setose partly | 0 |  |
|  | Outer margin of proximal segment setose entirely | 1 | 10 – Vereshchaka (2009) |
| **MALE CLASPING ORGAN** | | | |
| 116 | Absent | 0 | 4B |
|  | Present | 1 | 4C |
| 117 | A single clasping tubercle absent | 0 |  |
|  | A single clasping tubercle present | 1 |  |
| 118 | Two clasping tubercles absent | 0 |  |
|  | Two clasping tubercles present | 1 |  |
| 119 | One rudimentary and one well-developed clasping tubercles absent | 0 |  |
|  | One rudimentary and one well-developed clasping tubercles present | 1 |  |
| 120 | Two well-developed clasping tubercles absent | 0 |  |
|  | Two well-developed clasping tubercles present | 1 |  |
| 121 | Claw-like setae positioned in scattered pairs opposite to the tubercle absent | 0 |  |
|  | Claw-like setae positioned in scattered pairs opposite to the tubercle present | 1 |  |
| 122 | No claw-like setae positioned in two continuous row with gap | 0 |  |
|  | Claw-like setae positioned in two continuous row with gap present | 1 |  |
| 123 | Serrated bristles opposite to the tubercle absent | 0 |  |
|  | Serrated bristles opposite to the tubercle present | 1 |  |
| 124 | Serrated bristles with reticulate distal part absent | 0 |  |
|  | Serrated bristles with reticulate distal part present | 1 |  |
| 125 | Serrated bristles with longitudinal ribs in distal part absent | 0 |  |
|  | Serrated bristles with longitudinal ribs in distal part present | 1 |  |
| 126 | Scales opposite to the tubercle absent | 0 |  |
|  | Scales opposite to the tubercle present | 1 |  |
| 127 | A row of serrated bristles adjacent to the tubercle absent | 0 |  |
|  | A row of serrated bristles adjacent to the tubercle present | 1 |  |
| 128 | Strong distal tooth on the fourth segment absent | 0 |  |
|  | Strong distal tooth on the fourth segment present | 1 |  |
| **PETASMA** | | | |
| 129 | Pars astrigens present | 0 |  |
|  | Pars astrigens absent | 1 |  |
| 130 | Well-developed pars astrigens present | 0 |  |
|  | Well-developed pars astrigens absent | 1 |  |
| 131 | Vestigial pars astrigens absent present | 0 |  |
|  | Vestigial pars astrigens present | 1 |  |
| 132 | Pars externa not transformed into a wide unsupported sheath | 0 |  |
|  | Pars externa transformed into a wide unsupported sheath | 1 |  |
| 133 | Pars externa not transformed into a narrow sheath supported by chitinous rib | 0 |  |
|  | Pars externa transformed into a narrow sheath supported by chitinous rib | 1 |  |
| 134 | Pars externa without hook | 0 | 5C, 6D – Vereshchaka *et al*. (2014) |
|  | Pars externa with a hook | 1 | 5A,B,D – Vereshchaka *et al*. (2014) |
| 135 | Pars externa without terminal setae | 0 |  |
|  | Pars externa with terminal setae | 1 |  |
| 136 | Pars externa unarmed at the tip | 0 |  |
|  | Pars externa with fine friction structures at the tip | 1 |  |
| 137 | Pars externa not armed with transverse ribs along entire margin | 0 |  |
|  | Pars externa armed with transverse ribs along entire margin | 1 |  |
| 138 | Pars externa without plate-like structures in addition to friction structures | 0 |  |
|  | Pars externa with plate-like structures in addition to friction structures | 1 |  |
| 139 | Capitulum absent or vestigial | 0 |  |
|  | Capitulum present | 1 |  |
| 140 | Capitulum not armed with strong claws | 0 |  |
|  | Capitulum armed with strong claws | 1 |  |
| 141 | Divided capitulum without with strong claws | 0 |  |
|  | Divided capitulum armed with strong claws | 1 |  |
| 142 | Capitulum without squamose hooks in addition to ordinary claws | 0 |  |
|  | Capitulum with squamose hooks in addition to ordinary claws | 1 |  |
| 143 | Capitulum without enlarged claws in addition to ordinary claws | 0 |  |
|  | Capitulum with enlarged claws in addition to ordinary claws | 1 |  |
| 144 | Capitulum not armed with squamose hooks and pincers | 0 |  |
|  | Capitulum armed with squamose hooks and pincers | 1 |  |
| 145 | No entire capitulum armed with squamose hooks and pincers | 0 |  |
|  | Entire capitulum armed with squamose hooks and pincers present | 1 |  |
| 146 | No divided capitulum armed with squamose hooks and pincers | 0 |  |
|  | Divided capitulum armed with squamose hooks and pincers present | 1 |  |
| 147 | Not modified capitulum with pincers (all lobi and processi present and not divided) absent | 0 |  |
|  | Not modified capitulum with pincers (all lobi and processi present and not divided) present | 1 |  |
| 148 | Significantly modified (divided or reduced) capitulum with pincers lobi/processi absent | 0 |  |
|  | Significantly modified (divided or reduced) capitulum with pincers lobi/processi present | 1 |  |
| 149 | Lobus armatus absent | 0 |  |
|  | Lobus armatus present | 1 |  |
| 150 | Rudimentary lobus armatus absent | 0 |  |
|  | Rudimentary lobus armatus present | 1 | 5E – Vereshchaka *et al*. (2014) |
| 151 | Well-developed lobus armatus absent | 0 |  |
|  | Well-developed lobus armatus present | 1 | 5A-D – Vereshchaka *et al*. (2014) |
| 152 | Lobus connectens and lobus terminalis not twisted | 0 | 5A-E – Vereshchaka *et al*. (2014) |
|  | Lobus connectens and lobus terminalis twisted | 1 | 6D – Vereshchaka *et al*. (2014) |
| 153 | Lobus connectens absent | 0 | 5B – Vereshchaka *et al*. (2014) |
|  | Lobus connectens present | 1 | 4A,C-E – Vereshchaka *et al*. (2014) |
| 154 | Rudimentary lobus connectens absent | 0 |  |
|  | Rudimentary lobus connectens present | 1 | 5C – Vereshchaka *et al*. (2014) |
| 155 | Well-developed lobus connectens absent | 0 |  |
|  | Well-developed lobus connectens present | 1 | 5A,D,E – Vereshchaka *et al*. (2014) |
| 156 | Entire lobus connectens absent | 0 |  |
|  | Entire lobus connectens present | 1 | 5A,C,D – Vereshchaka *et al*. (2014) |
| 157 | Terminally divided lobus connectens absent | 0 |  |
|  | Terminally divided lobus connectens present | 1 | 5E – Vereshchaka *et al*. (2014) |
| 158 | Lobus connectens with additional lobe at base directed upward absent | 0 |  |
|  | Lobus connectens with additional lobe at base directed upward present | 1 | 6C – Vereshchaka *et al*. (2014) |
| 159 | Lobus connectens with additional lobe at base directed downward absent | 0 |  |
|  | Lobus connectens with additional lobe at base directed downward present | 1 |  |
| 160 | Lobus connectens not swan-shaped | 0 | 6B,C – Vereshchaka *et al*. (2014) |
|  | Lobus connectens swan-shaped | 1 | 6E – Vereshchaka *et al*. (2014) |
| 161 | Lobus connectens without pillow at base | 0 | 6C,D – Vereshchaka *et al*. (2014) |
|  | Lobus connectens with pillow at base | 1 | 6E – Vereshchaka *et al*. (2014) |
| 162 | Apex of lobus connectens not bearing a single much enlarged sucker with a hook inside | 0 | 5D,E – Vereshchaka *et al*. (2014) |
|  | Apex of lobus connectens bearing a single much enlarged sucker with a hook inside | 1 | 6E – Vereshchaka *et al*. (2014) |
| 163 | Curved lobus inermis absent | 0 |  |
|  | Curved lobus inermis present | 1 | 6A – Vereshchaka *et al*. (2014) |
| 164 | Straight lobus inermis absent | 0 |  |
|  | Straight lobus inermis present | 1 | 5A,B,E – Vereshchaka *et al*. (2014) |
| 165 | Inflated lobus inermis absent | 0 |  |
|  | Inflated lobus inermis present | 1 | 6A – Vereshchaka *et al*. (2014) |
| 166 | Narrow lobus inermis absent | 0 |  |
|  | Narrow lobus inermis present | 1 | 5B – Vereshchaka *et al*. (2014) |
| 167 | Lobus terminalis absent or rudimentary | 0 |  |
|  | Lobus terminalis developed | 1 | 5A-D – Vereshchaka *et al*. (2014) |
| 168 | Entire lobus terminalis absent | 0 |  |
|  | Entire lobus terminalis present | 1 | 5A-D – Vereshchaka *et al*. (2014) |
| 169 | Terminally divided lobus terminalis absent | 0 |  |
|  | Terminally divided lobus terminalis present | 1 | 6A – Vereshchaka *et al*. (2014) |
| 170 | Lobus terminalis with additional lobe at base absent | 0 |  |
|  | Lobus terminalis with additional lobe at base present | 1 | 1C, 3C – Vereshchaka *et al*. (2014) |
| 171 | Processus ventralis absent | 0 | 5C – Vereshchaka *et al*. (2014) |
|  | Processus ventralis present | 1 | 5A,B,D, – Vereshchaka *et al*. (2014) |
| 172 | Rudimentary processus ventralis absent | 0 |  |
|  | Rudimentary processus ventralis present | 1 | 5E – Vereshchaka *et al*. (2014) |
| 173 | Developed rocessus ventralis absent | 0 |  |
|  | Developed rocessus ventralis present | 1 | 5A,B,D – Vereshchaka *et al*. (2014) |
| 174 | Processus ventralis without lateral friction structures | 0 |  |
|  | Processus ventralis with lateral friction structures | 1 |  |
| 175 | Processus ventralis without minute apical setae | 0 |  |
|  | Processus ventralis with minute apical setae | 1 |  |
| 176 | Entire processus ventralis absent | 0 |  |
|  | Entire processus ventralis present | 1 | 5A,B,D, – Vereshchaka *et al*. (2014) |
| 177 | Divided processus ventralis absent | 0 |  |
|  | Divided processus ventralis present | 1 | 2C, 4C – present paper |
| 178 | Twice divided processus ventralis absent | 0 |  |
|  | Twice divided processus ventralis present | 1 |  |
| 179 | Entire elongate processus ventralis absent | 0 |  |
|  | Entire elongate processus ventralis present | 1 | 5A,B,D – Vereshchaka *et al*. (2014) |
| 180 | Triangle processus ventralis absent | 0 |  |
|  | Triangle processus ventralis present | 1 | 5E – Vereshchaka *et al*. (2014) |
| 181 | Needle-like elongate processus ventralis absent | 0 |  |
|  | Needle-like elongate processus ventralis present | 1 | 5E – Vereshchaka *et al*. (2014) |
| 182 | Processus ventralis without hooks and sucks | 0 | 5A-E – Vereshchaka *et al*. (2014) |
|  | Processus ventralis with hooks and sucks | 1 | 6B – Vereshchaka *et al*. (2014) |
| 183 | Processus ventralis without simple spines | 0 | 5A,C,E – Vereshchaka *et al*. (2014) |
|  | Processus ventralis with simple spines | 1 | 5B,D – Vereshchaka *et al*. (2014) |
| 184 | Processus ventralis without simple spines | 0 |  |
|  | Processus ventralis with simple spines | 1 |  |
| 185 | Processus ventralis without >4 simple spines | 0 |  |
|  | Processus ventralis with >4 simple spines | 1 |  |
| 186 | Processus ventralis without stellate spines | 0 | 5A,D,E – Vereshchaka *et al*. (2014) |
|  | Processus ventralis with stellate spines | 1 | 5B – Vereshchaka *et al*. (2014) |
| 187 | Processus ventralis without apical lashes | 0 | 5B-E – Vereshchaka *et al*. (2014) |
|  | Processus ventralis with apical lashes | 1 | 5A– Vereshchaka *et al*. (2014) |
| 188 | Processus ventralis without 5-25 apical lashes in row | 0 |  |
|  | Processus ventralis with 5-25 apical lashes in row | 1 | 5A– Vereshchaka *et al*. (2014) |
| 189 | Processus ventralis without two pincer-like apical lashes | 0 |  |
|  | Processus ventralis with two pincer-like apical lashes | 1 | 5A– Vereshchaka *et al*. (2014) |
| **PHOTOPHORES** | | | |
| 190 | The organ of Pesta absent | 0 | 2F – Vereshchaka *et al*. (2014) |
|  | The organ of Pesta present | 1 | 2E – Vereshchaka *et al*. (2014) |
| 191 | No organ of Pesta with anterolateral spheroid organs | 0 |  |
|  | The organ of Pesta with anterolateral spheroid organs present | 1 | 12 – Vereshchaka (2009) |
| 192 | No organ of Pesta with anterolateral lobed organ | 0 |  |
|  | The organ of Pesta with anterolateral lobed organ present | 1 | 12 – Vereshchaka (2009) |
| 193 | No organ of Pesta with posterolateral spheroid organs | 0 |  |
|  | The organ of Pesta with posterolateral spheroid organs present | 1 | 12 – Vereshchaka (2009) |
| 194 | No organ of Pesta with posterolateral fringed organs | 0 |  |
|  | The organ of Pesta with posterolateral fringed organs present | 1 | 12 – Vereshchaka (2009) |
| 195 | No organ of Pesta with 3 distinct organs (2 lateral midgastric, and 1 continuous posterior fringe) | 0 |  |
|  | The organ of Pesta with 3 distinct organs (2 lateral midgastric, and 1 continuous posterior fringe) present | 1 | 12 – Vereshchaka (2009) |
| 196 | No organ of Pesta with 6-8 distinct organs (1 anteromedian, 2 lateral midgastric, 2 posterolateral fringes) | 0 |  |
|  | The organ of Pesta with 6-8 distinct organs (1 anteromedian, 2 lateral midgastric, 2 posterolateral fringes) present | 1 | 12 – Vereshchaka (2009) |
| 197 | No organ of Pesta with 1 distinct posteromedian organ | 0 |  |
|  | The organ of Pesta with 1 distinct posteromedian organ present | 1 | 12 – Vereshchaka (2009) |
| 198 | No organ of Pesta with : 2-3 distinct organs (2 posterolateral and, in some species, 1 posteromedian) | 0 |  |
|  | The organ of Pesta with : 2-3 distinct organs (2 posterolateral and, in some species, 1 posteromedian) present | 1 | 12 – Vereshchaka (2009) |
| 199 | No organ of Pesta with : 3 distinct organs (1 anteromedian, and 2 posterolateral) | 0 |  |
|  | The organ of Pesta with : 3 distinct organs (1 anteromedian, and 2 posterolateral) present | 1 | 12 – Vereshchaka (2009) |
| 200 | No organ of Pesta with 4 distinct organs (1 anteromedian, 2 posterolateral, and 1 posteromedian) | 0 |  |
|  | The organ of Pesta with 4 distinct organs (1 anteromedian, 2 posterolateral, and 1 posteromedian) present | 1 | 12 – Vereshchaka (2009) |
| 201 | No organ of Pesta with 5 distinct organs (1 anteromedian, 2 lateral midgastric, and 2 posterolateral) | 0 |  |
|  | The organ of Pesta with 5 distinct organs (1 anteromedian, 2 lateral midgastric, and 2 posterolateral) present | 1 | 12 – Vereshchaka (2009) |
| 202 | Dermal photophores absent | 0 |  |
|  | Dermal photophores present | 1 |  |
| 203 | Lens-less dermal photophores absent | 0 |  |
|  | Lens-less dermal photophores present | 1 | 7C-F – Vereshchaka *et al*. (2014) |
| 204 | Lens-bearing dermal photophores absent | 0 |  |
|  | Lens- bearing dermal photophores present | 1 | 7A,B – Vereshchaka *et al*. (2014) |
| 205 | A total of 130-170 photophores absent | 0 |  |
|  | A total of 130-170 photophores present | 1 |  |
| 206 | A total of 190-210 photophores absent | 0 |  |
|  | A total of 190-210 photophores present | 1 |  |
| 207 | A total of 225 or more photophores absent | 0 |  |
|  | A total of 225 or more photophores present | 1 |  |
| 208 | Two lateral rows of photophores on carapace absent | 0 |  |
|  | Two lateral rows of photophores on carapace present | 1 | 7B – Vereshchaka *et al*. (2014) |
| 209 | A single lateral row of photophores on carapace absent | 0 |  |
|  | A single lateral row of photophores on carapace present | 1 | 7A – Vereshchaka *et al*. (2014) |
| 210 | Fixed number of photophores in the upper row on carapace absent | 0 |  |
|  | Fixed number of photophores in the upper row on carapace present | 1 |  |
| 211 | Not fixed number of photophores in the upper row on carapace absent | 0 |  |
|  | Not fixed number of photophores in the upper row on carapace present | 1 |  |
| 212 | Four or more photophores in the upper row on carapace absent | 0 |  |
|  | Four or more photophores in the upper row on carapace present | 1 | 7A,B – Vereshchaka *et al*. (2014) |
| 213 | Two-three photophores in the upper row on carapace absent | 0 |  |
|  | Two-three photophores in the upper row on carapace present | 1 |  |
| 214 | Not fixed number of photophores on scaphocerite absent | 0 |  |
|  | Not fixed number of photophores on scaphocerite present | 1 |  |
| 215 | Fixed number of photophores on scaphocerite absent | 0 |  |
|  | Fixed number of on scaphocerite present | 1 |  |
| 216 | Eight or more photophores on scaphocerite absent | 0 |  |
|  | Eight or more photophores on scaphocerite present | 1 | 7C,E – Vereshchaka *et al*. (2014) |
| 217 | Seven photophores on scaphocerite absent | 0 |  |
|  | Seven photophores on scaphocerite present | 1 |  |
| 218 | Four to six photophores on scaphocerite absent | 0 |  |
|  | Four to six photophores on scaphocerite present | 1 | 7G – Vereshchaka *et al*. (2014) |
| 219 | Two or three photophores on scaphocerite absent | 0 |  |
|  | Two or three photophores on scaphocerite present | 1 |  |
| 220 | Small photophores on scaphocerite absent | 0 |  |
|  | Small photophores on scaphocerite present | 1 | 7E – Vereshchaka *et al*. (2014) |
| 221 | Medium-sized photophores on scaphocerite absent | 0 |  |
|  | Medium-sized photophores on scaphocerite present | 1 |  |
| 222 | Large photophores on scaphocerite absent | 0 |  |
|  | Large photophores on scaphocerite present | 1 | 7C – Vereshchaka *et al*. (2014) |
| 223 | Separated from each other photophores on scaphocerite absent | 0 |  |
|  | Separated from each other photophores on scaphocerite present | 1 | 7E – Vereshchaka *et al*. (2014) |
| 224 | Partly fused photophores on scaphocerite absent | 0 |  |
|  | Partly fused photophores on scaphocerite present | 1 | 7C – Vereshchaka *et al*. (2014) |
| 225 | Two rows of photophores on scaphocerite absent | 0 |  |
|  | Two rows of photophores on scaphocerite present | 1 | 7C – Vereshchaka *et al*. (2014) |
| 226 | A single row of photophores on scaphocerite absent | 0 |  |
|  | A single row of photophores on scaphocerite present | 1 | 7G,E – Vereshchaka *et al*. (2014) |
| 227 | A contiguous row of equally spaced from each other photophores on uropodal exopod absent | 0 |  |
|  | A contiguous row of equally spaced from each other photophores on uropodal exopod present | 1 | 7F – Vereshchaka *et al*. (2014) |
| 228 | Two groups of photophores separated by a gap on uropodal exopod absent | 0 |  |
|  | Two groups of photophores separated by a gap on uropodal exopod present | 1 | 7D,H – Vereshchaka *et al*. (2014) |
| 229 | Small photophores on uropodal exopod absent | 0 |  |
|  | Small photophores on uropodal exopod present | 1 | 7F,H – Vereshchaka *et al*. (2014) |
| 230 | Medium-sized photophores on uropodal exopod absent | 0 |  |
|  | Medium-sized photophores on uropodal exopod present | 1 |  |
| 231 | Large photophores on uropodal exopod absent | 0 |  |
|  | Large photophores on uropodal exopod present | 1 | 7D – Vereshchaka *et al*. (2014) |
| 232 | Partly fused photophores on uropodal exopod absent | 0 |  |
|  | Partly fused photophores on uropodal exopod present | 1 | 7D – Vereshchaka *et al*. (2014) |
| 233 | Separated from each other photophores on uropodal exopod absent | 0 |  |
|  | Separated from each other photophores on uropodal exopod present | 1 | 7F – Vereshchaka *et al*. (2014) |
| 234 | Photophores on basal segment of uropodal exopod positioned closer to central axis absent | 0 |  |
|  | Photophores on basal segment of uropodal exopod positioned closer to central axis present | 1 | 7D,H – Vereshchaka *et al*. (2014) |
| 235 | Photophores on basal segment of uropodal exopod positioned closer margin absent | 0 |  |
|  | Photophores on basal segment of uropodal exopod positioned closer to margin present | 1 | 7F – Vereshchaka *et al*. (2014) |
| 236 | Not fixed number of photophores on basal segment of uropodal exopod absent | 0 |  |
|  | Not fixed number of photophores on basal segment of uropodal exopod present | 1 |  |
| 237 | Fixed number of photophores on basal segment of uropodal exopod absent | 0 |  |
|  | Fixed number of on basal segment of uropodal exopod present | 1 |  |
| 238 | Three or more photophores on basal segment of uropodal exopod absent | 0 | 7F – Vereshchaka *et al*. (2014) |
|  | Three or more photophores on basal segment of uropodal exopod present | 1 |  |
| 239 | Two photophores on basal segment of uropodal exopod absent | 0 |  |
|  | Two photophores on basal segment of uropodal exopod present | 1 |  |
| 240 | A single photophore on basal segment of uropodal exopod absent | 0 |  |
|  | A single photophore on basal segment of uropodal exopod present | 1 | 7H – Vereshchaka *et al*. (2014) |
| 241 | Photophores on distal segment of uropodal exopod positioned closer to central axis absent | 0 |  |
|  | Photophores on distal segment of uropodal exopod positioned closer to central axis present | 1 | 7D,H – Vereshchaka *et al*. (2014) |
| 242 | Photophores on distal segment of uropodal exopod positioned closer margin absent | 0 |  |
|  | Photophores on distal segment of uropodal exopod positioned closer to margin present | 1 | 7F – Vereshchaka *et al*. (2014) |
| 243 | Two rows or triangle of photophores on distal segment of uropodal exopod absent | 0 |  |
|  | Two rows or triangle of photophores on distal segment of uropodal exopod present | 1 | 7D – Vereshchaka *et al*. (2014) |
| 244 | A single row of photophores on distal segment of uropodal exopod absent | 0 |  |
|  | A single row of photophores on distal segment of uropodal exopod present | 1 | 7F,H – Vereshchaka *et al*. (2014) |
| 245 | Not fixed number of photophores on distal segment of uropodal exopod absent | 0 |  |
|  | Not fixed number of photophores on distal segment of uropodal exopod present | 1 |  |
| 246 | Fixed number of photophores on distal segment of uropodal exopod absent | 0 |  |
|  | Fixed number of on distal segment of uropodal exopod present | 1 |  |
| 247 | Three or more photophores on distal segment of uropodal exopod absent | 0 | 7F – Vereshchaka *et al*. (2014) |
|  | Three or more photophores on distal segment of uropodal exopod present | 1 | 7F – Vereshchaka *et al*. (2014) |
| 248 | A single photophore on distal segment of uropodal exopod absent | 0 |  |
|  | A single photophore on distal segment of uropodal exopod present | 1 | 7H – Vereshchaka *et al*. (2014) |
| **LARVA** | | | |
| 249 | Metamorphosis of usual dendrbranchiate type | 0 |  |
|  | Metamorphosis elaborated, including elaphocaris and acanthosoma stages | 1 |  |
| 250 | Elaphocaris stage of *dohrni* type absent | 0 |  |
|  | Elaphocaris stage of *dohrni* type present | 1 | 2 – Vereshchaka (2009) |
| 251 | Elaphocaris stage of *hispida* type absent | 0 |  |
|  | Elaphocaris stage of *hispida* type present | 1 | 2 – Vereshchaka (2009) |
| 252 | Elaphocaris stage of *ortmanni* type absent | 0 |  |
|  | Elaphocaris stage of *ortmanni* type present | 1 | 2 – Vereshchaka (2009) |

^1^ - very thin and soft, in intact specimens with regular riffles.

^2^ - variations.

Appendix 4. Data matrix. Missing data indicated by question marks (?); inapplicable data by hyphens (-)

Characters 0 - 49

0 5 10 15 20 25 30 35 40 45

| | | | | | | | | |

Gennadas parvus 10100010001000000000000100000000000000000000100000

Penaeus monodon 00000001001000000000000100000010000000100000100100

Lucifer typus 01101101010110011001001001000110010101001111100100

Lucifer orientalis 01101101010110011001001001000110010101001111100100

Belsebub intermedius 01101101010110011001001001000110011010001100100100

Belsebub penicillifer 01101101010110011001001001000110011010001100100100

Belsebub faxoni 01101101010110011001001001000110011010001100100100

Belsebub chacei 01101101010110011001001001000110011010001100100100

Belsebub hanseni 01101101010110011001001001000110011010001100100100

Acetes americanus 00001001010010001001000101000010000000100000010001

Acetes binghami 00101001010010001001000101000010000000100000010001

Acetes chinensis 00001001010010001001000101000010000000100000010001

Acetes erythraeus 00001001010010001001000101000010000000100000010001

Acetes indicus 00001001010010001001000101000010000000100000010001

Acetes intermedius 00001001010010001001000101000010000000100000010001

Acetes japonicus 00001001010010001001000101000010000000100000010001

Acetes johni 00001001010010001001000101000010000000100000010001

Acetes natalensis 00001001010010001001000101000010000000100000010001

Acetes serrulatus 00001001010010001001000101000010000000100000010001

Acetes sibogae 00001001010010001001000101000010000000100000010001

Acetes vulgaris 00001001010010001001000101000010000000100000010001

Acetes marinus 00001001010010001001000101000010000000100000010001

Acetes paraguayensis 00001001010010001001000101000010000000100000010001

Acetes petrunkevitchi 00001001010010001001000101000010000000100000010001

Sicyonella inermis 00001101010001000100100100110001000000010000100001

Sicyonella maldivensis 00001101010001000100100100110001000000010000100001

Sicyonella antennata 00001101010001000100100100110001000000010000100001

Petalidium obesum 10100000110000100010010010101000100000100000000001

Petalidium foliaceum 10100001010000100010010010101010100000100000000001

Petalidium suspiriosum 10100001010000100010010010101001000000100000000001

Sergia tenuiremis 10100010001000100010010100010001000000100000100010

Sergia inoa 10100010001000100010010100010001000000100000100010

Sergia remipes 10100010001000100010010100010001000000100000100010

Sergia laminata 10100010001000100010010100010001000000100000100010

Gardinerosergia bigemmea 00100000101000100010010100010001000000100000100010

Gardinerosergia gardneri 00100000101000100010010100010001000000100000100010

Gardinerosergia inequalis 00100000101000100010010100010001000000100000100010

Gardinerosergia kensleyi 00100000101000100010010100010001000000100000100010

Gardinerosergia splendens 00100000101000100010010100010001000000100000100010

Phorcosergia bisulcata 00100000101000100010010100010001000000100000100010

Phorcosergia burukovskii 00100000101000100010010100010001000000100000100010

Phorcosergia filicta 00100000101000100010010100010001000000100000100010

Phorcosergia grandis 00100000101000100010010100010001000000100000100010

Phorcosergia maxima 00100000101000100010010100010001000000100000100010

Phorcosergia phorca 00100000101000100010010100010001000000100000100010

Phorcosergia plumea 00100000101000100010010100010001000000100000100010

Phorcosergia potens 00100000101000100010010100010001000000100000100010

Phorcosergia wolffi 00100000101000100010010100010001000000100000100010

Robustosergia extenuata 00100000101000100010010100010001000000100000100010

Robustosergia regalis 00100000101000100010010100010001000000100000100010

Robustosergia robusta 00100000101000100010010100010001000000100000100010

Robustosergia vityazi 00100000101000100010010100010001000000100000100010

Prehensilosergia prehensilis 00100000101000100010010100010001000000100000100010

Scintillosergia scintillans 00100000101000100010010100010001000000100000100010

Challengerosergia challengeri 00100001001000100010010100010001000000100000101010

Challengerosergia fulgens 00100001001000100010010100010001000000100000101010

Challengerosergia hansjacobi 00100001001000100010010100010001000000100000100010

Challengerosergia jeppeseni 00100001001000100010010100010001000000100000100010

Challengerosergia oksanae 00100001001000100010010100010001000000100000101010

Challengerosergia stellata 00100001001000100010010100010001000000100000101010

Challengerosergia talismani 00100001001000100010010100010001000000100000100010

Challengerosergia umitakae 00100001001000100010010100010001000000100000100010

Lucensosergia crosnieri 00100001001000100010010100010001000000100000100010

Lucensosergia colosii 00100001001000100010010100010001000000100000100010

Lucensosergia foresti 00100001001000100010010100010001000000100000100010

Lucensosergia lucens 00100001001000100010010100010001000000100000100010

Deosergestes coalitus 00100001001000100010010100010001000000100000000010

Deosergestes corniculum 00100001001000100010010100010001000000100000000010

Deosergestes disjunctus 00101001001000100010010100010001000000100000000010

Deosergestes henseni 00101001001000100010010100010001000000100000000010

Deosergestes paraseminudus 00100001001000100010010100010001000000100000000010

Deosergestes pediforms 00100001001000100010010100010001000000100000000010

Deosergestes rubroguttatus 00100001001000100010010100010001000000100000000010

Deosergestes seminudus 00100001001000100010010100010001000000100000000010

Eusergestes arcticus 00101001001000100010010100010001000000100000100010

Eusergestes similis 00101001001000100010010100010001000000100000100010

Eusergestes antarcticus 00101001001000100010010100010001000000100000100010

Sergestes atlanticus 00101001001000100010010100101001000000100000000001

Cornutosergestes cornutus 00101001001000100010010100101001000000100000000001

Cornutosergestes mepae 00101001001000100010010100101001000000100000000001

Allosergestes index 00110001001000100010010100101001000000100000000000

Allosergestes nudus 00111001001000100010010100101001000000100000000000

Allosergestes oleseni 00110001001000100010010100101001000000100000000000

Allosergestes pectinatus 00111001001000100010010100101001000000100000000000

Allosergestes pestafer 00111001001000100010010100101001000000100000000000

Allosergestes sargassi 00110001001000100010010100101001000000100000000000

Allosergestes verpus 00110001001000100010010100101001000000100000000000

Allosergestes vinogradovi 00110001001000100010010100101001000000100000000000

Parasergestes armatus 00100001001000100010010100101001000000100000000000

Parasergestes cylindricus 00100001001000100010010100101001000000100000000000

Parasergestes diapontius 00100001001000100010010100101001000000100000000000

Parasergestes halia 00100001001000100010010100101001000000100000000000

Parasergestes sirenkoi 00100001001000100010010100101001000000100000000000

Parasergestes stimulator 00100001001000100010010100101001000000100000000000

Parasergestes vigilax 00100001001000100010010100101001000000100000000000

Neosergestes brevispinatus 00100001001000100010010100101001000000100000000010

Neosergestes consobrinus 00100001001000100010010100101001000000100000000010

Neosergestes edwardsi 00100001001000100010010100101001000000100000000010

Neosergestes orientalis 00100001001000100010010100101001000000100000000010

Neosergestes semissis 00100001001000100010010100101001000000100000000010

Neosergestes tantillus 00100001001000100010010100101001000000100000000010

Characters 50 - 99

50 55 60 65 70 75 80 85 90 95

| | | | | | | | | |

Gennadas parvus 10100000000000000000001000000010000000010010001000

Penaeus monodon 00100000000000000000001000000010000000010010001000

Lucifer typus 21001011101000000000010000100000001100101100010000

Lucifer orientalis 21001011101000000000010000100000001100101100010000

Belsebub intermedius 21001011101000000000010000100000001100101100010000

Belsebub penicillifer 21001011101000000000010000100000001100101100010000

Belsebub faxoni 21001011101000000000010000100000001100101100010000

Belsebub chacei 21001011101000000000010000100000001100101100010000

Belsebub hanseni 21001011101000000000010000100000001100101100010000

Acetes americanus 00000100100000100000000100001010100001010100010000

Acetes binghami 00000100100000100000000100001010100001010100010000

Acetes chinensis 00000100100000100000000100001010100001010100010000

Acetes erythraeus 00000100100000100000000100001010100001010100010000

Acetes indicus 00000100100000100000000100001010110001010100010000

Acetes intermedius 00000100100000100000000100001010110001010100010000

Acetes japonicus 00000100100000100000000100001010100001010100010000

Acetes johni 00000100100000100000000100001010100001010100010000

Acetes natalensis 00000100100000100000000100001010000001010100010000

Acetes serrulatus 00000100100000100000000100001010100001010100010000

Acetes sibogae 00000100100000100000000100001010110001010100010000

Acetes vulgaris 00000100100000100000000100001010100001010100010000

Acetes marinus 00000100100000100000000100001010100001010100010000

Acetes paraguayensis 00000100100000100000000100001010100001010100010000

Acetes petrunkevitchi 00000100100000100000000100001010000001010000100010

Sicyonella inermis 00001000000101011000001000010010000010010010001001

Sicyonella maldivensis 00001000000101011000001000010010000010010010001001

Sicyonella antennata 00001000000101011000001000010010000010010010001001

Petalidium obesum 0001000000000?????????????001???00?001???001000101

Petalidium foliaceum 0001000000000?????????????001???00?001???001000101

Petalidium suspiriosum 0001000000000?????????????001???00?001???001000101

Sergia tenuiremis 00100000000001100000010000001010100001010001000100

Sergia inoa 00100000000001100000010000001010100001010001000100

Sergia remipes 00100000000001100000010000001010100001010001000100

Sergia laminata 00100000000001100000010000001010100001010001000100

Gardinerosergia bigemmea 20100000000001100000010000001010100001010001000100

Gardinerosergia gardneri 20100000000001100000010000001010100001010001000100

Gardinerosergia inequalis 20100000000001100000010000001010100001010001000100

Gardinerosergia kensleyi 20100000000001100000010000001010100001010001000100

Gardinerosergia splendens 20100000000001100000010000001010100001010001000100

Phorcosergia bisulcata 00100000000001100000010000001010100001010001000100

Phorcosergia burukovskii 00100000000001100000010000001010100001010001000100

Phorcosergia filicta 00100000000001100000010000001010100001010001000100

Phorcosergia grandis 00100000000001100000010000001010100001010001000100

Phorcosergia maxima 00100000000001100000010000001010100001010001000100

Phorcosergia phorca 00100000000001100000010000001010100001010001000100

Phorcosergia plumea 00100000000001100000010000001010100001010001000100

Phorcosergia potens 00100000000001100000010000001010100001010001000100

Phorcosergia wolffi 00100000000001100000010000001010100001010001000100

Robustosergia extenuata 00100000000001100000010000001010100001010001000100

Robustosergia regalis 00100000000001100000010000001010100001010001000100

Robustosergia robusta 00100000000001100000010000001010100001010001000100

Robustosergia vityazi 00100000000001100000010000001010100001010001000100

Prehensilosergia prehensilis 10100000000001100000010000001010100001010001000100

Scintillosergia scintillans 10100000000001100000010000001010100001010001000100

Challengerosergia challengeri 10100000000001100000010000001010100001010001000100

Challengerosergia fulgens 10100000000001100000010000001010100001010001000100

Challengerosergia hansjacobi 10100000000001100000010000001010100001010001000100

Challengerosergia jeppeseni 10100000000001100000010000001010100001010001000100

Challengerosergia oksanae 10100000000001100000010000001010100001010001000100

Challengerosergia stellata 10100000000001100000010000001010100001010001000100

Challengerosergia talismani 10100000000001100000010000001010100001010001000100

Challengerosergia umitakae 10100000000001100000010000001010100001010001000100

Lucensosergia crosnieri 10100000000001100000010000001010100001010001000100

Lucensosergia colosii 10100000000001100000010000001010100001010001000100

Lucensosergia foresti 10100000000001100000010000001010100001010001000100

Lucensosergia lucens 10100000000001100000010000001010100001010001000100

Deosergestes coalitus 10100000000001010001010000001011101001101001000100

Deosergestes corniculum 10100000000001010010010000001011101001101001000100

Deosergestes disjunctus 10100000000001010011010000001011101001101001000100

Deosergestes henseni 10100000000001010010010000001011101001101001000100

Deosergestes paraseminudus 10100000000001010001010000001011101001101001000100

Deosergestes pediforms 10100000000001010001010000001011101001101001000100

Deosergestes rubroguttatus 10100000000001010001010000001011101001101001000100

Deosergestes seminudus 10100000000001010001010000001011101001101001000100

Eusergestes arcticus 10100000000011010010010000001010100001010001000101

Eusergestes similis 10100000000011010010010000001010100001010001000101

Eusergestes antarcticus 10100000000011010010010000001010100001010001000101

Sergestes atlanticus 20100000000001010001010000001010000001010001000101

Cornutosergestes cornutus 20100000110001010001010000001010000001010001000101

Cornutosergestes mepae 20100000110001010001010000001010000001010001000101

Allosergestes index 10100000000101010100010000001100101001101001000100

Allosergestes nudus 10100000000101010010010000001100101001101001000100

Allosergestes oleseni 10100000000101010100010000001100101001101001000100

Allosergestes pectinatus 10100000000101010100010000001100101001101001000100

Allosergestes pestafer 10100000000101010100010000001100101001101001000100

Allosergestes sargassi 10100000000101010100010000001100101001101001000100

Allosergestes verpus 10100000000101010100010000001100101001101001000100

Allosergestes vinogradovi 10100000000101010100010000001100101001101001000100

Parasergestes armatus 20100000000101011000110010001010100001010001000101

Parasergestes cylindricus 20100000000101011000110010001010100001010001000101

Parasergestes diapontius 20100000000101011000110010001010100001010001000101

Parasergestes halia 20100000000101011000110010001010100001010001000101

Parasergestes sirenkoi 20100000000101011000110010001010100001010001000101

Parasergestes stimulator 20100000000101011000110010001010100001010001000101

Parasergestes vigilax 20100000000101011000110010001010100001010001000101

Neosergestes brevispinatus 20100000000101010010010011001010000001010001000101

Neosergestes consobrinus 20100000000101010010010011001010000001010001000101

Neosergestes edwardsi 20100000000101010010010011001010000001010001000101

Neosergestes orientalis 20100000000101010010010011001010000001010001000101

Neosergestes semissis 20100000000101010010010011001010000001010001000101

Neosergestes tantillus 20100000000101010010010011001010000001010001000101

Characters 100 - 149

100 105 110 115 120 125 130 135 140 145

| | | | | | | | | |

Gennadas parvus 00100010000100000000000000000000000000000000000000

Penaeus monodon 00100010000100000000000000000000000000000000000000

Lucifer typus 01000100000100000000000000001101010010100000000000

Lucifer orientalis 01000100000100000000000000001101000010100000000000

Belsebub intermedius 01000100000100000000000000001100101101100000000000

Belsebub penicillifer 01000100000100000000000000001100101101100000000000

Belsebub faxoni 01000100000100000000000000001100101101100000000000

Belsebub chacei 01000100000100000000000000001100101101100000000000

Belsebub hanseni 01000100000100000000000000001100101101100000000000

Acetes americanus 01000000100100011110001010011100000000111010000000

Acetes binghami 01000000100100011110001010011100000000111010000000

Acetes chinensis 01000000100100011101100000001100000000110100000000

Acetes erythraeus 01000000100100011110100000000000000000110101000000

Acetes indicus 01000000100100011110100000001100000000110100000000

Acetes intermedius 01000000100100011110100000000000000000110101000000

Acetes japonicus 01000000100100011101100000001100000000110100000000

Acetes johni 01000000100100011101100000011100000000111000000000

Acetes natalensis 01000000100100011101100000010010000000110100000000

Acetes serrulatus 01000000100100011101100000011100000000110101000000

Acetes sibogae 01000000100100011110100000000000000000110101000000

Acetes vulgaris 01000000100100011110100000000000000000110101000000

Acetes marinus 01000000100100011110001101010000000000000000000000

Acetes paraguayensis 01000000100100011110001101010000000000000000000000

Acetes petrunkevitchi 00001000100100011110001100010000010000111000000000

Sicyonella inermis 00100010010100010000000001000000000000100000110110

Sicyonella maldivensis 00100010010100010000010000000000000000100000101011

Sicyonella antennata 00100010010100010000010000000000000000100000101011

Petalidium obesum 00010001010100010000001101100000010000100000101011

Petalidium foliaceum 00010001010100010000001101100000010000100000101011

Petalidium suspiriosum 00010001010100010000001101100000010000100000101011

Sergia tenuiremis 10010001001100011000000001000000010000100000101101

Sergia inoa 10010001001100011000000001000000010000100000101101

Sergia remipes 10010001001100011000000001000000010000100000101101

Sergia laminata 10010001001100011000000001000000010000100000101101

Gardinerosergia bigemmea 10010001001100011000001101000000010000100000101011

Gardinerosergia gardneri 10010001001100011000001101000000010000100000101011

Gardinerosergia inequalis 10010001001100011000001101000000010000100000101011

Gardinerosergia kensleyi 10010001001100011000001101000000010000100000101011

Gardinerosergia splendens 10010001001100011000001101000000010000100000101011

Phorcosergia bisulcata 10010001001100011000001101000000010000100000101011

Phorcosergia burukovskii 10010001001100011000001101000000010000100000101011

Phorcosergia filicta 10010001001100011000001101000000010000100000101011

Phorcosergia grandis 10010001001100011000001101000000010000100000101011

Phorcosergia maxima 10010001001100011000001101000000010000100000101011

Phorcosergia phorca 10010001001100011000001101000000010000100000101011

Phorcosergia plumea 10010001001100011000001101000000010000100000101011

Phorcosergia potens 10010001001100011000001101000000010000100000101011

Phorcosergia wolffi 10010001001100011000001101000000010000100000101011

Robustosergia extenuata 10010001001100011000001101000000010000100000101011

Robustosergia regalis 10010001001100011000001101000000010000100000101011

Robustosergia robusta 10010001001100011000001101000000010000100000101011

Robustosergia vityazi 10010001001100011000001101000000010000100000101011

Prehensilosergia prehensilis 10010001001100011000001101000000010000100000101011

Scintillosergia scintillans 10010001001100011000001101000000010000100000101011

Challengerosergia challengeri 10010001001100011000001101000000010000100000101011

Challengerosergia fulgens 10010001001100011000001101000000010000100000101011

Challengerosergia hansjacobi 10010001001100011000001101000000010000100000101011

Challengerosergia jeppeseni 10010001001100011000001101000000010000100000101011

Challengerosergia oksanae 10010001001100010000001101000000010000100000101011

Challengerosergia stellata 10010001001100011000001101000000010000100000101011

Challengerosergia talismani 10010001001100010000001101000000010000100000101011

Challengerosergia umitakae 10010001001100011000001101000000010000100000101011

Lucensosergia crosnieri 10010001001100011000001101000000010000100000101010

Lucensosergia colosii 10010001001100011000001101000000010000100000101011

Lucensosergia foresti 10010001001100011000001101000000010000100000101011

Lucensosergia lucens 10010001001100011000001101000000010000100000101011

Deosergestes coalitus 10010001001011011000000001000000010000100000101011

Deosergestes corniculum 10010001001011011000000001000000010000100000101011

Deosergestes disjunctus 10010001001011011000000001000000010000100000101011

Deosergestes henseni 10010001001011011000000001000000010000100000101011

Deosergestes paraseminudus 10010001001011011000000001000000010000100000101011

Deosergestes pediforms 10010001001011011000000001000000010000100000101011

Deosergestes rubroguttatus 10010001001011011000000001000000010000100000101011

Deosergestes seminudus 10010001001011011000000001000000010000100000101011

Eusergestes arcticus 00010001010100011000000001000000010000100000101011

Eusergestes similis 00010001010100011000000001000000010000100000101011

Eusergestes antarcticus 00010001010100011000000001000000010000100000101011

Sergestes atlanticus 00010001010100011000000001000000000000100000101011

Cornutosergestes cornutus 00010001010100011000000001000000000000100000101011

Cornutosergestes mepae 00010001010100011000000001000000000000100000101011

Allosergestes index 10010001001011011000000001000000010000100000101011

Allosergestes nudus 10010001001011011000000001000000010000100000101011

Allosergestes oleseni 10010001001011010000000001000000010000100000101011

Allosergestes pectinatus 10010001001011011000000001000000010000100000101011

Allosergestes pestafer 10010001001011011000000001000000010000100000101011

Allosergestes sargassi 10010001001011011000000001000000010000100000101011

Allosergestes verpus 10010001001011011000000001000000010000100000101011

Allosergestes vinogradovi 10010001001011011000000001000000010000100000101011

Parasergestes armatus 00010001010011011000000001000000000000100000101011

Parasergestes cylindricus 00010001010011011000000001000000000000100000101011

Parasergestes diapontius 00010001010011011000000001000000000000100000101011

Parasergestes halia 00010001010011011000000001000000000000100000101011

Parasergestes sirenkoi 00010001010011011000000001000000000000100000101011

Parasergestes stimulator 00010001010011011000000001000000000000100000101011

Parasergestes vigilax 00010001010011011000000001000000000000100000101011

Neosergestes brevispinatus 00010001010010111000000001000000000000100000101011

Neosergestes consobrinus 00010001010010111000000001000000000000100000101011

Neosergestes edwardsi 00010001010010111000000001000000000000100000101011

Neosergestes orientalis 00010001010010111000000001000000000000100000101011

Neosergestes semissis 00010001010010111000000001000000000000100000101011

Neosergestes tantillus 00010001010010111000000001000000000000100000101011

Characters 150 - 199

150 155 160 165 170 175 180 185 190 195

| | | | | | | | | |

Gennadas parvus 00000000000000000000000000000000000000000000000000

Penaeus monodon 00000000000000000000000000000000000000000000000000

Lucifer typus 00000000000000000000011000100100000001010000000000

Lucifer orientalis 00000000000000000000011000100100000001010000000000

Belsebub intermedius 00000000000000000000011000100001000000000000000000

Belsebub penicillifer 00000000000000000000011011100001000000000000000000

Belsebub faxoni 00000000000000000000011010100001000000000000000000

Belsebub chacei 00000000000000000000011010100001000000000000000000

Belsebub hanseni 00000000000000000000011010100001000000000000000000

Acetes americanus 00000000000000000000000000000000000000000000000000

Acetes binghami 00000000000000000000000000000000000000000000000000

Acetes chinensis 00000000000000000000011000100001000000000000000000

Acetes erythraeus 00000000000000000000011000100100000000000000000000

Acetes indicus 00000000000000000000011000100001000000000000000000

Acetes intermedius 00000000000000000000011000100100000000000000000000

Acetes japonicus 00000000000000000000011000100001000000000000000000

Acetes johni 00000000000000000000000000000000000000000000000000

Acetes natalensis 00000000000000000000011000100001000000000000000000

Acetes serrulatus 00000000000000000000000000000000000000000000000000

Acetes sibogae 00000000000000000000011000100100000000000000000000

Acetes vulgaris 00000000000000000000011000100100000000000000000000

Acetes marinus 00000000000000000000000000000000000000000000000000

Acetes paraguayensis 00000000000000000000000000000000000000000000000000

Acetes petrunkevitchi 00000000000000000000000000000000000000000000000000

Sicyonella inermis 00000000000000000000000000000000000000000000000000

Sicyonella maldivensis 01010110000000000100111000011000100000000000000000

Sicyonella antennata 01010110000000000100111000011000100000000000000000

Petalidium obesum 01011010000000000100111000010000100000000000000000

Petalidium foliaceum 01011010000000000100111000010000100000000000000000

Petalidium suspiriosum 01011010000000000100111000010000100000000000000000

Sergia tenuiremis 01010110000000101110011000100100000000000000000000

Sergia inoa 01010110000000101110011000100100000000000000000000

Sergia remipes 01010110000000101110011000100100000000000000000000

Sergia laminata 01010110000000101110011000100100000000000000000000

Gardinerosergia bigemmea 01010110000000101110011000100100000000000000000000

Gardinerosergia gardneri 01010100100000000110011000100100000000000000000000

Gardinerosergia inequalis 01010110000000101110011000100100000000000000000000

Gardinerosergia kensleyi 01010110000000101110011000100100000000000000000000

Gardinerosergia splendens 01010110000000000110011000100100000000000000000000

Phorcosergia bisulcata 01010100011000101110011000100100000000000000000000

Phorcosergia burukovskii 01010100011000101110011000100100000000000000000000

Phorcosergia filicta 01010100011000101110011000100100000000000000000000

Phorcosergia grandis 01010100011000101110011000100100000000000000000000

Phorcosergia maxima 01010110001000101110011000100100000000000000000000

Phorcosergia phorca 01010100011000101110011000100100000000000000000000

Phorcosergia plumea 01010100011000101110011000100100000000000000000000

Phorcosergia potens 01010100011000101100111000100100000000000000000000

Phorcosergia wolffi 01010100011000101110011000100100000000000000000000

Robustosergia extenuata 01110110001110101110011000100100000000000000000000

Robustosergia regalis 01110110001110101110011000100100000000000000000000

Robustosergia robusta 01110110001110101110011000100100000000000000000000

Robustosergia vityazi 01110110001110101110011000100100000000000000000000

Prehensilosergia prehensilis 01010101000000101110011000100100000000000000000000

Scintillosergia scintillans 01010110000000110000000000000000000000000000000000

Challengerosergia challengeri 10010110000001010101011000100100000000000000000000

Challengerosergia fulgens 10010110000001010101011000100100000000000000000000

Challengerosergia hansjacobi 10010110000001010101011000100100000000000000000000

Challengerosergia jeppeseni 10010110000001010101011000100100000000000000000000

Challengerosergia oksanae 10010110000001010101011000100100000000000000000000

Challengerosergia stellata 10010110000000000101011000100100000000000000000000

Challengerosergia talismani 10010110000001010101011000100100000000000000000000

Challengerosergia umitakae 10010110000001010110011000100100000000000000000000

Lucensosergia crosnieri 00011010000000000000011000100100100000000000000000

Lucensosergia colosii 10011010000000101110011000100100100000000000000000

Lucensosergia foresti 10011010000000000110011000100100100000000000000000

Lucensosergia lucens 10011010000000000110011000100100100000000000000000

Deosergestes coalitus 01010110000000101110011000100100000001101101001000

Deosergestes corniculum 01010110000000101110011000100100000001101101001000

Deosergestes disjunctus 01010110000000101110011000100100000001101101001000

Deosergestes henseni 01010110000000101110011000100100000001101101001000

Deosergestes paraseminudus 01010110000000101110011000100100000001101101001000

Deosergestes pediforms 01010110000000101110011000100100000001101101001000

Deosergestes rubroguttatus 01010110000000101110011000100100000001101101001000

Deosergestes seminudus 01010110000000101110011000100100000001101101001000

Eusergestes arcticus 01010110000000000110011000100100011100001101010000

Eusergestes similis 01010110000000000110011000100100011100001101010000

Eusergestes antarcticus 01010110000000000110011000100100011100001101010000

Sergestes atlanticus 10010101000000101110011000100010000000001010100000

Cornutosergestes cornutus 01011010000000000110000-00000000000000001010100100

Cornutosergestes mepae 01011010000000001110000-00000000000000001010100100

Allosergestes index 01000000000000101110011000100100000010001010100010

Allosergestes nudus 01000000000000101110011000100100010100001010100010

Allosergestes oleseni 01000000000000101110011000100100011000001010100010

Allosergestes pectinatus 01000000000000000110011000100100011000001010100010

Allosergestes pestafer 01000000000000101110011000100100010110001010100010

Allosergestes sargassi 01000000000000101110011000100100010110001010100010

Allosergestes verpus 01000000000000000110011000100100010110001010100010

Allosergestes vinogradovi 01000000000000000110011000100100010110001010100010

Parasergestes armatus 01011010000000110110010100100100000000001010100001

Parasergestes cylindricus 01011010000000110110010100100100000000001010100001

Parasergestes diapontius 01011010000000110110010100100100000000001010100001

Parasergestes halia 01011010000000110110010100100100000000001010100001

Parasergestes sirenkoi 01011010000000110110010100100100000000001010100001

Parasergestes stimulator 01011010000000110110010100100100000000001010100001

Parasergestes vigilax 01011010000000110110010100100100000000001010100001

Neosergestes brevispinatus 01010101000000110100110100100100000000001010100000

Neosergestes consobrinus 01010101000000110100110100100100000000001010100000

Neosergestes edwardsi 01010101000000110100110100100100000000001010100000

Neosergestes orientalis 01010101000000110100110100100100000000001010100000

Neosergestes semissis 01010110000000110100110100100100000000001010100000

Neosergestes tantillus 01010110000000110100110100100100000000001010100000

Characters 200 - 249

200 205 210 215 220 225 230 235 240 245

| | | | | | | | | |

Gennadas parvus 00000000000000000000000000000000000000000000000000

Penaeus monodon 00000000000000000000000000000000000000000000000000

Lucifer typus 00000000000000000000000000000000000000000000000000

Lucifer orientalis 00000000000000000000000000000000000000000000000000

Belsebub intermedius 00000000000000000000000000000000000000000000000000

Belsebub penicillifer 00000000000000000000000000000000000000000000000000

Belsebub faxoni 00000000000000000000000000000000000000000000000000

Belsebub chacei 00000000000000000000000000000000000000000000000000

Belsebub hanseni 00000000000000000000000000000000000000000000000000

Acetes americanus 00000000000000000000000000000000000000000000000000

Acetes binghami 00000000000000000000000000000000000000000000000000

Acetes chinensis 00000000000000000000000000000000000000000000000000

Acetes erythraeus 00000000000000000000000000000000000000000000000000

Acetes indicus 00000000000000000000000000000000000000000000000000

Acetes intermedius 00000000000000000000000000000000000000000000000000

Acetes japonicus 00000000000000000000000000000000000000000000000000

Acetes johni 00000000000000000000000000000000000000000000000000

Acetes natalensis 00000000000000000000000000000000000000000000000000

Acetes serrulatus 00000000000000000000000000000000000000000000000000

Acetes sibogae 00000000000000000000000000000000000000000000000000

Acetes vulgaris 00000000000000000000000000000000000000000000000000

Acetes marinus 00000000000000000000000000000000000000000000000000

Acetes paraguayensis 00000000000000000000000000000000000000000000000000

Acetes petrunkevitchi 00000000000000000000000000000000000000000000000000

Sicyonella inermis 00000000000000000000000000000000000000000000000000

Sicyonella maldivensis 00000000000000000000000000000000000000000000000000

Sicyonella antennata 00000000000000000000000000000000000000000000000000

Petalidium obesum 00000000000000000000000000000000000000000000000000

Petalidium foliaceum 00000000000000000000000000000000000000000000000000

Petalidium suspiriosum 00000000000000000000000000000000000000000000000000

Sergia tenuiremis 00000000000000000000000000000000000000000000000001

Sergia inoa 00000000000000000000000000000000000000000000000001

Sergia remipes 00000000000000000000000000000000000000000000000001

Sergia laminata 00000000000000000000000000000000000000000000000001

Gardinerosergia bigemmea 00110001101010101000100100110100011010100100101011

Gardinerosergia gardneri 00110001101010101000100100110100011010100100101011

Gardinerosergia inequalis 00110001101010101000100100110100011010100100101011

Gardinerosergia kensleyi 00110001101010101000100100110100011010100100101011

Gardinerosergia splendens 00110001101010101000100100110100011010100100101011

Phorcosergia bisulcata 00110001101010101000001011001001100110100011001011

Phorcosergia burukovskii 00110001101010101000001011001001100110100011001011

Phorcosergia filicta 00110001101010101000001011001001100110100011001011

Phorcosergia grandis 00110001101010101000001011001001100110100011001011

Phorcosergia maxima 00110001101010101000001011001001100110100011001011

Phorcosergia phorca 00110001101010101000001011001001100110100011001011

Phorcosergia plumea 00110001101010101000001011001001100110100011001011

Phorcosergia potens 00110001101010101000001011001001100110100011001011

Phorcosergia wolffi 00110001101010101000001011001001100110100011001011

Robustosergia extenuata 00110001101010101000010100101010010110100010101011

Robustosergia regalis 00110001101010101000010100101010010110100010101011

Robustosergia robusta 00110001101010101000010100101010010110100010101011

Robustosergia vityazi 00110001101010101000010100101010010110100010101011

Prehensilosergia prehensilis 00101001101010101000100100101100010110000010101011

Scintillosergia scintillans 00101001100110010100100100101100010101010010110101

Challengerosergia challengeri 00101010010110010010100100101100010101001010110101

Challengerosergia fulgens 00101010010110010010100100101100010101001010110101

Challengerosergia hansjacobi 00101010010110010010100100101100010101001010110101

Challengerosergia jeppeseni 00101010010110010010100100101100010101001010110101

Challengerosergia oksanae 00101010010110010010100100101100010101001010110101

Challengerosergia stellata 00101010010110010010100100101100010101001010110101

Challengerosergia talismani 00101010010110010010100100101100010101001010110101

Challengerosergia umitakae 00101010010110010010100100101100010101001010110101

Lucensosergia crosnieri 00101100010101010001100100101100010101001010110001

Lucensosergia colosii 00101100010101010001100100101100010101001010110001

Lucensosergia foresti 00101100010101010001100100101100010101001010110001

Lucensosergia lucens 00101100010101010001100100101100010101001010110101

Deosergestes coalitus 00000000000000000000000000000000000000000000000001

Deosergestes corniculum 00000000000000000000000000000000000000000000000001

Deosergestes disjunctus 00000000000000000000000000000000000000000000000001

Deosergestes henseni 00000000000000000000000000000000000000000000000001

Deosergestes paraseminudus 00000000000000000000000000000000000000000000000001

Deosergestes pediforms 00000000000000000000000000000000000000000000000001

Deosergestes rubroguttatus 00000000000000000000000000000000000000000000000001

Deosergestes seminudus 00000000000000000000000000000000000000000000000001

Eusergestes arcticus 00000000000000000000000000000000000000000000000001

Eusergestes similis 00000000000000000000000000000000000000000000000001

Eusergestes antarcticus 00000000000000000000000000000000000000000000000001

Sergestes atlanticus 10000000000000000000000000000000000000000000000001

Cornutosergestes cornutus 00000000000000000000000000000000000000000000000001

Cornutosergestes mepae 00000000000000000000000000000000000000000000000001

Allosergestes index 00000000000000000000000000000000000000000000000001

Allosergestes nudus 00000000000000000000000000000000000000000000000001

Allosergestes oleseni 00000000000000000000000000000000000000000000000001

Allosergestes pectinatus 00000000000000000000000000000000000000000000000001

Allosergestes pestafer 00000000000000000000000000000000000000000000000001

Allosergestes sargassi 00000000000000000000000000000000000000000000000001

Allosergestes verpus 00000000000000000000000000000000000000000000000001

Allosergestes vinogradovi 00000000000000000000000000000000000000000000000001

Parasergestes armatus 00000000000000000000000000000000000000000000000001

Parasergestes cylindricus 00000000000000000000000000000000000000000000000001

Parasergestes diapontius 00000000000000000000000000000000000000000000000001

Parasergestes halia 00000000000000000000000000000000000000000000000001

Parasergestes sirenkoi 00000000000000000000000000000000000000000000000001

Parasergestes stimulator 00000000000000000000000000000000000000000000000001

Parasergestes vigilax 00000000000000000000000000000000000000000000000001

Neosergestes brevispinatus 01000000000000000000000000000000000000000000000001

Neosergestes consobrinus 01000000000000000000000000000000000000000000000001

Neosergestes edwardsi 01000000000000000000000000000000000000000000000001

Neosergestes orientalis 01000000000000000000000000000000000000000000000001

Neosergestes semissis 01000000000000000000000000000000000000000000000001

Neosergestes tantillus 01000000000000000000000000000000000000000000000001


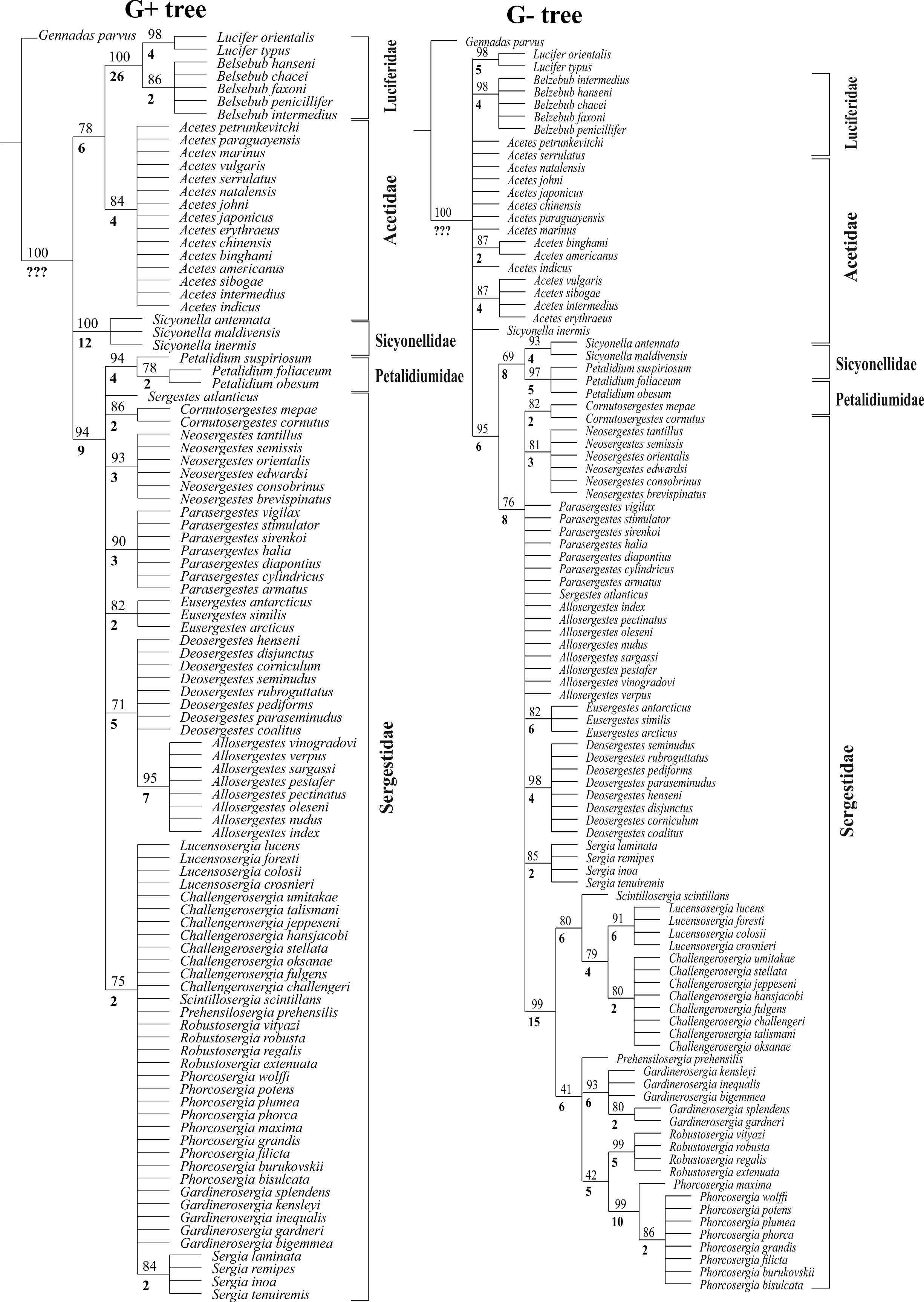


Appendix 5. The strict consensus tree and the clades retrieved after analysis with *Gennadas parvus* as the outgroup, with the bootstrap support (bold numbers above the clade) and the Bremer support (numbers below the clade): the G+ tree with only general external characters included in the matrix (left) and the G- tree with all but general external characters included in the matrix (right).


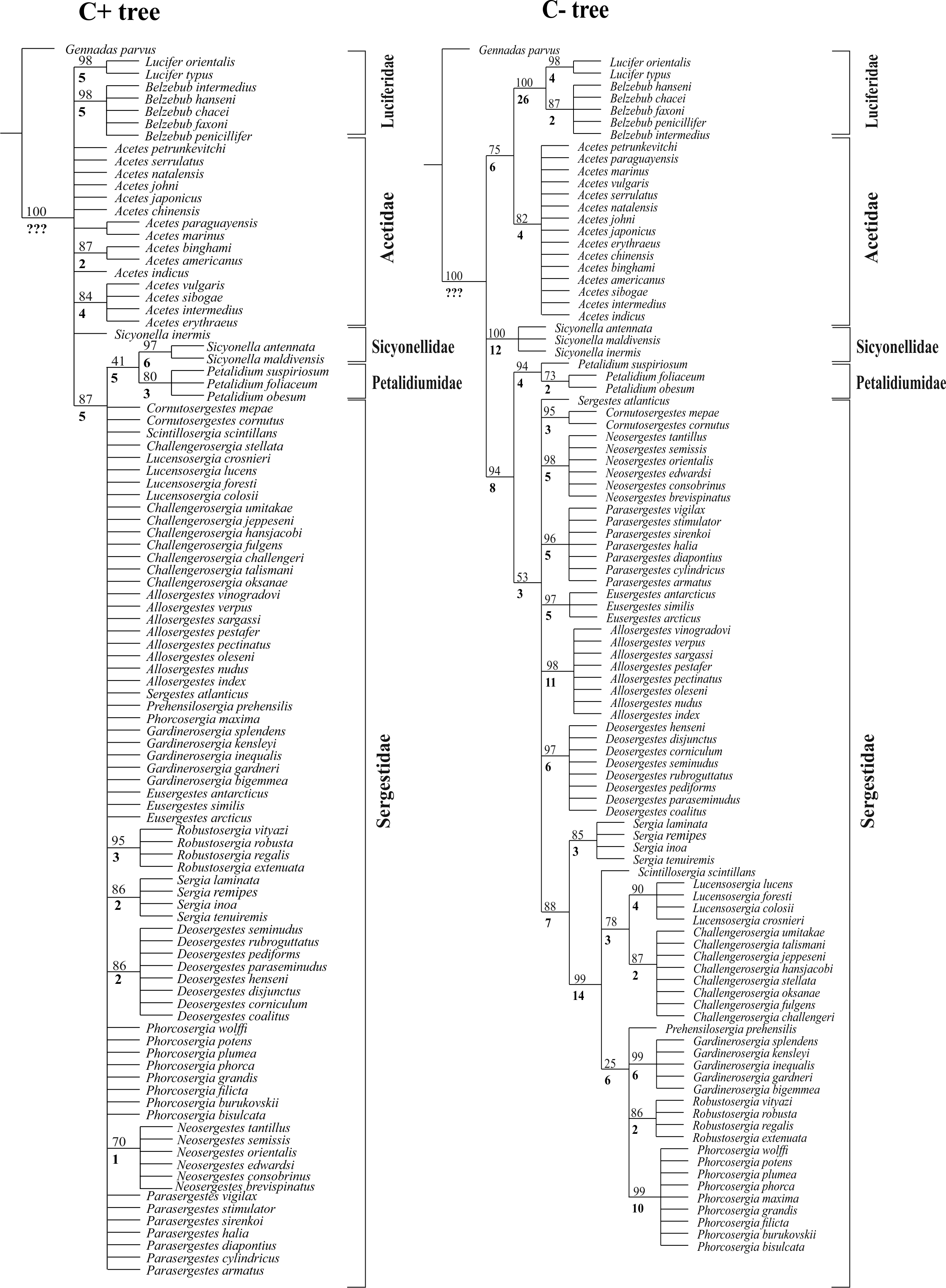


Appendix 6. The strict consensus tree and the supported clades retrieved after analysis with *Gennadas parvus* as the outgroup, with the bootstrap support (bold numbers above the clade) and the Bremer support (numbers below the clade): the C+ tree with only copulatory characters included in the matrix (left) and the C- tree with all but copulatory characters included in the matrix (right).


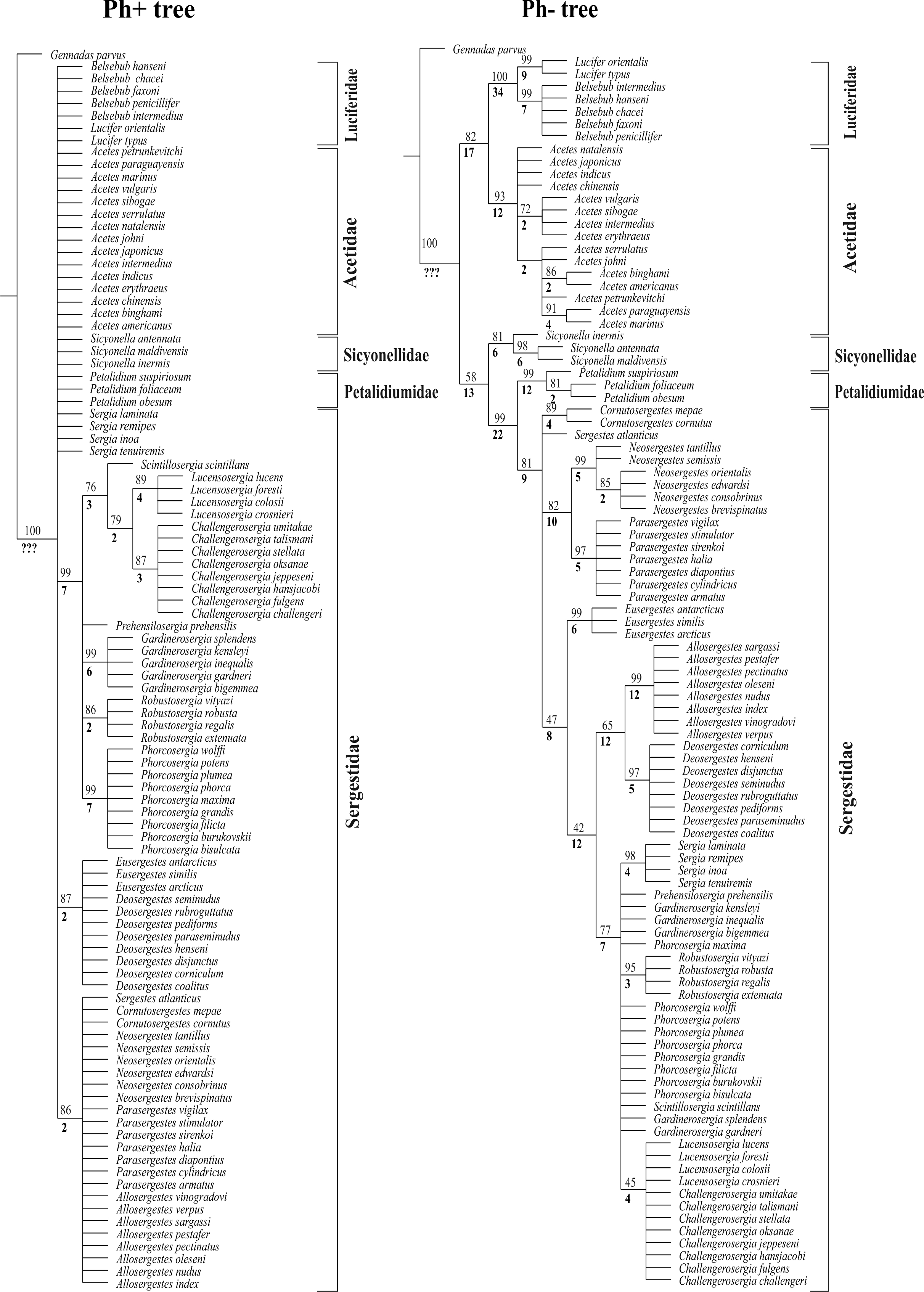


Appendix 7. The strict consensus tree and the supported clades retrieved after analysis with *Gennadas parvus* as the outgroup, with the bootstrap support (bold numbers above the clade) and the Bremer support (numbers below the clade): the Ph+ tree with only photophores included in the matrix (left) and the Ph- tree with all characters but photophores included in the matrix (right).

Appendix 8. Emended diagnoses and keys to major taxa of Sergestoidea Dana, 1852

SUPERFAMILY SERGESTOIDEA DANA, 1852

Diagnosis: Carapace compressed, rostrum shorter than eyestalks. Antennula with ventral flagellum modified to clasping organ in males or absent. Fourth and fifth pereopods either absent or flattened and natatory. Branchiae: pleurobranchs absent, two or less arthrobranchs on each side of somite, eight or less pairs of well-developed dendrite branchs on each side.

Key to families of the superfamily Sergestoidea

1. Labrum widely separated from antennae and eyes, telson with two pairs of lateral spines; second pereopod without chela, third pereopod with subchela. Males with two ventral processes on sixth abdominal somite, with strong ventral protuberance on telson, clasping organ absent . Luciferidae De Haan, 1849

- Labrum not widely separated from antennae and eyes, telson with four pairs of lateral spines or without spines; second and third pereopods with slightly reduced chela (2-3 times as long as fingers) 2

2. Pterygostomial tooth present, telson with four pairs of lateral spines, fourth and fifth pereopods with seven segments Sicyonellidae **fam. nov.**

- Pterygostomial tooth absent, telson without lateral spines, fourth and fifth pereopods with six or less segments 3

3. Third antennular segment elongate (>1.5 as long as first segment), fourth and fifth pereopods, if present, with five or less segments Acetidae **fam. nov.**

- Third antennular segment not elongate (<1.5 as long as first segment), fourth and fifth pereopods with six segments 4

4. Maximum height of rostrum at middle of its length. Male clasping organ bearing two opposite rows of serial bristles on inner side Petalidiumidae **fam. nov.**

- Maximum height of rostrum near tip. Serial bristles, if any, only on one side of male clasping organ Sergestidae Dana, 1852

FAMILY ACETIDAE **FAM. NOV.**

Diagnosis: Rostrum acute, with two or more dorsal teeth behind the orbital margin; carapace with postorbital and hepatic teeth, pterygostomial tooth absent, labrum not greatly separated from antennae and eyes; sixth abdominal somite in male without ventral processes; telson without lateral spines and ventral protuberance. Antennule in male with ventral flagellum and clasping organ bearing two tubercles; mandible and maxillae with palp, maxillula with a single endite; first maxilliped with epipod and exopod; second maxilliped with epipod, third maxilliped not elongated (less than twice as long as first pereopod), with entire dactyl; first to third pereopods with greatly reduced chela (palm >10 times as long as fingers), fourth and fifth pereopods reduced (1-5 segments) or absent. Photophores: absent. Petasma: reduced to some extent. Branchiae: arthrobranchs on somite VIII, developed anterior arthrobranch on somite XII, posterior arthrobranchs on somites IX-XII.

Genus included: *Acetes* H. Milne Edwards, 1830 (15 species).

Diagnosis of the genus and key to species may be found in [10]

FAMILY LUCIFERIDAE DE HAAN, 1849

Diagnosis: Rostrum short, acute, oblique; carapace greatly compressed laterally, with postorbital, pterygostomial, and hepatic teeth, labrum widely separated from antennae and eyes; sixth abdominal somite in male bearing two ventral processes; telson with two pairs of lateral spines, strong ventral protuberance present in male. Antennule without ventral flagellum and clasping organ; mandible and maxillae lacking palp, maxillula with two endites; first maxilliped lacking epipod and exopod, second maxilliped lacking epipod, third maxilliped not elongated (less than twice as long as first pereopod), with entire dactyl; with entire dactyl; first and second pereopods without chelae; third pereopod with subchela, distal end of propodus bearing strong, curved teeth and serrated setae; fourth and fifth pereopods absent in both sexes. Photophores: absent. Petasma: developed, pars astrigens absent, pars externa developed, transformed into a sheath around long, entire processus ventralis. Branchiae: absent.

Genera included: *Lucifer* Thompson, 1829 (2 species) and *Belzebub* Vereshchaka, Olesen and Lunina, 2016 (5 species).

Key to genera of the family Luciferidae

1. Posterior ventral process on sixth male abdominal somite curved, not tapering, apically obtuse. Eyestalks elongated, conical, nearly reaching end of scaphocerite. Petasma: sheath wide, entirely armed with transverse chitinous ribs along inner margin, without additional plate-like structures; processus ventralis lamellar, with apical pincer ……………..….……........ *Lucifer*

– Posterior ventral process on sixth male abdominal somite nearly straight, tapering, apically subacute. Eyestalks of moderate length, subcylindrical, not reaching end of scaphocerite. Petasma: sheath narrow, tapering, supported by strong chitinous ribs, armed with apical scales or of ridges, with additional plate-like structures; processus ventralis spiniform, without apical pincer .......................................................................................................................... *Belzebub*

Diagnoses of the genera and key to species may be found in [11].

FAMILY PETALIDIUMIDAE **FAM. NOV.**

Diagnosis: Rostrum acute, with a single dorsal tooth behind the orbital margin; carapace without postorbital and pterygostomial teeth, hepatic prominence as barb or tooth, labrum not greatly separated from antennae and eyes; sixth abdominal somite in male without ventral processes; telson without lateral spines and ventral protuberance. Antennule in male with ventral flagellum and clasping organ bearing two opposite rows of serial bristles on inner side; mandible and maxillae with palp, maxillula with three endites; first maxilliped with epipod and exopod; second maxilliped with epipod, third maxilliped not elongated (less than twice as long as first pereopod); second and third pereopods with greatly reduced chela (>10 times as long as fingers); fourth and fifth pereopods 6-segmented. Photophores: absent. Petasma: well-developed, capitulum armed with squamous hooks and pincers. Branchiae: arthrobranchs on somite VIII, rudimentary anterior arthrobranch on somite XII, rudimentary lamellar posterior arthrobranchs on somites IX-XI.

Genus included: *Petalidium* Spence Bate, 1881 (3 species).

Diagnosis of the genus and key to species may be found in [9].

FAMILY SERGESTIDAE DANA, 1852

Diagnosis: Rostrum 0-1 dorsal tooth behind the orbital margin; carapace without pterygostomial tooth, hepatic prominence as barb or tooth, labrum not greatly separated from antennae and eyes; sixth abdominal somite in male without ventral processes; telson without lateral spines and ventral protuberance. Antennule in male with ventral flagellum and clasping organ; mandible and maxillae with palp, maxillula with four endites; first maxilliped with epipod and exopod; second maxilliped with epipod, third maxilliped with subdivided dactyl; first pereopod without chela, second and third pereopods with greatly reduced chela (>10 times as long as fingers); fourth and fifth pereopods 6-segmented. Photophores: as dermal organs or organ of Pesta, or absent. Petasma: well-developed, capitulum armed with squamous hooks and pincers. Branchiae: podobranchs on somite VIII, developed anterior arthrobranch on somite XII, rudimentary lamellar posterior arthrobranchs on somites IX-XI.

Genera included: *Allosergestes* Judkins & Kensley, 2008 (8 species), *Challengerosergia* Vereshchaka, Olesen and Lunina, 2016 (8 species), *Cornutosergestes* Vereshchaka, Olesen and Lunina, 2016 (2 species), *Deosergestes* Judkins & Kensley, 2008 (8 species), *Eusergestes* Judkins & Kensley, 2008 (3 species), *Gardinerosergia* Vereshchaka, Olesen and Lunina, 2016 (5 species), *Lucensosergia* Vereshchaka, Olesen and Lunina, 2016 (4 species), *Neosergestes* Judkins & Kensley, 2008 (6 species), *Parasergestes* Judkins & Kensley, 2008 (7 species), *Phorcosergia* Vereshchaka, Olesen and Lunina, 2016 (9 species), *Prehensilosergia* Vereshchaka, Olesen and Lunina, 2016 (1 species), *Robustosergia* Vereshchaka, Olesen and Lunina, 2016 (4 species), *Scintillosergia* Vereshchaka, Olesen and Lunina, 2016 (1 species), *Sergestes* H. Milne-Edwards, 1830 (1 species), *Sergia* Stimpson, 1860 (4 species).

Key to genera of the family Sergestidae

1. Organ of Pesta absent. Body opaque in live specimens, or, if semi-transparent, with dermal photophores 2

- Organ of Pesta present. Body semi-transparent in live specimens, without dermal photophores 9

2. Integument membranous, dermal photophores absent *Sergia*

- Integument firm, dermal photophores present 3
3. Dermal photophores without lens, visible as opaque spots 4

- Dermal photophores with lens 6

4. Photophores as large, partly fused organs, arranged in 2 rows on scaphocerite and a triangular patch on uropodal exopod *Phorcosergia*

- Photophores small, not fused, arranged in 1 row on scaphocerite, and 1 row (randomly reduced to 1 organ) on uropodal exopod 5

5. Ocular papilla developed (>0.3 times as long as wide). LC of petasma without pillow at base, not twisted with LT, LT entire. Photophores on uropodal exopod positioned close to inner margin *Gardinerosergia*

- Ocular papilla rudimentary (<0.3 times as long as wide). LC of petasma with pillow at base, twisted with LT, LT divided. Photophores on uropodal exopod positioned close to median line *Robustosergia*

6. Photophores: in 2 lateral rows on carapace, 7 or more organs on scaphocerite 7

- Photophores: in a single lateral row on carapace, 6 or fewer organs on scaphocerite 8

7. Photophores: 7 organs on scaphocerite, 2 organs on proximal segment and 1 on distal segment of uropodal exopod. Petasma: LC divided, LI inflated, LT rudimentary, PV absent *Scintillosergia*

- Photophores: 10-15 organs on scaphocerite, 4-8 organs on proximal segment and 3-5 on distal segment of uropodal exopod. Petasma: LC entire, LI slender, LT well-developed, PV present *Prehensilosergia*

8. Photophores: 4-6 organs both on lateral carapace row and on scaphocerite. Petasma: PV without hooks and suckers *Challengerosergia*

- Photophores: 2-3 organs both on lateral carapace row and on scaphocerite. Petasma: PV with hooks and suckers *Lucensosergia*

9. Outer margin of uropodal exopod with tooth, not setose along proximal segment (proximal to the tooth) 10

- Outer margin of uropodal exopod without tooth, setose at least along part of proximal segment.. 12

10. First segment of antennule elongate, ≥1.5 times as long as 3rd segment, distal tooth of scaphocerite not overreaching blade, maxilliped III sexually dimorphic. Petasma: PU with hook, PV with simple spines. Arthrobranch: posterior lobe on segment XII (above pereopod III) dendritic *Eusergestes*

- First segment of antennule not elongate, <1.5 times as long as 3rd segment, distal tooth of scaphocerite overreaching blade, maxilliped III sexually not dimorphic. Petasma: PU without hook, PV unarmed. Arthrobranch: posterior lobe on segment XII (above pereopod III) lamellar 11

11. Rostrum triangular, not reaching middle of eyestalk. Endopod of maxilliped I with 3 segments. Petasma: LA rudimentary, LC developed, divided, PV present *Sergestes*

- Rostrum elongate, much overreaching middle of eyestalk. Endopod of maxilliped I with 2 segments. Petasma: LA developed, LC rudimentary, PV absent *Cornutosergestes*

12. Maxilliped III moderately elongated, < 2.0 times as long as carapace; chela of pereopod II with very long setae. Arthrobranch: posterior lobe on segment XII (above pereopod III) dendritic *Deosergestes*

- Maxilliped III much elongated, > 2.0 times as long as carapace; chela of pereopod II without very long setae. Arthrobranch: posterior lobe on segment XII (above pereopod III) lamellar 13

13. Rostrum with vertical frontal margin and beak-like terminal tooth, ocular papilla prominent, distal tooth of scaphocerite not overreaching blade, maxilliped III > 2.8 times as long as carapace, pereopod II without distally curved hooks on ischium, without protrusion on merus; chela with unequal fingers, pereopod III with strong curved spines proximal to tufts of long setae on propodus, pereopod V with distal segment setose along both margins. Petasma: LC absent, LI rudimentary, slender, PU with hook, PV developed *Allosergestes*

- Rostrum with oblique frontal margin, no beak-like terminal tooth, ocular papilla uncertain, distal tooth of scaphocerite much overreaching blade, maxilliped III 2.0-2.8 times as long as carapace, pereopod II with distally curved hooks on ischium and protrusion on merus; chela with subequal fingers, pereopod III without strong curved spines proximal to tufts of long setae on propodus, pereopod V with distal segment setose along one margin. Petasma: LC present, LI developed, inflated, PU without hook, PV rudimentary 14

14. Maxilliped III dactyl subdivided into 4 specialized subsegments, pereopod I with strong movable spines on ischium, outer margin of uropodal exopod setose partly *Parasergestes*

- Maxilliped III dactyl subdivided into 6 specialized subsegments, pereopod I without strong movable spines on ischium, outer margin of uropodal exopod setose entirely *Neosergestes*

Diagnoses of the genera and key to species may be found in [4].

FAMILY SICYONELLIDAE **FAM. NOV.**

Diagnosis: Rostrum acute, with two or more dorsal teeth behind the orbital margin; carapace with postorbital, pterygostomial, and hepatic teeth, labrum not greatly separated from antennae and eyes; sixth abdominal somite in male without ventral processes; telson with four lateral spines, ventral protuberance absent. Antennule in male with ventral flagellum and clasping organ; mandible and maxillae with palp, maxillula with two endites; first maxilliped with epipod and exopod; second maxilliped with epipod, third maxilliped elongated (at least twice as long as first pereopod) with dactyl subdivided into four specialized subsegments; first pereopod with developed chela (palm as long as fingers), second and third pereopods with slightly reduced chela (2-3 times as long as fingers); fourth and fifth pereopods 7-segmented. Photophores: dermal organs and organ of Pesta absent. Petasma: well-developed, capitulum armed with squamous hooks and pincers. Branchiae: arthrobranchs on somite VIII, developed anterior arthrobranch on somite XII, reduced dendritic posterior arthrobranchs on somites IX-XI.

Genus included: *Sicyonella* Borradaile, 1910 (3 species).

Diagnosis of the genus and key to species may be found in [10].
