# Supplementary material for: The shrimp superfamily Sergestoidea: a global phylogeny with definition of new families and an assessment of the pathways into principal biotopes
Source: R Soc Open Sci. 2017 Sep 6;4(9):170221. doi: 10.1098/rsos.170221 (PMC5627073; doi:10.1098/rsos.170221)
Supplement: Appendix 2 [file rsos170221supp2.docx]

Appendix 2. Changes in the synonymy of Sergestoidea not indicated in [30] or made later on.

| No | Old name | New name | Source |
| --- | --- | --- | --- |
| 1 | *Deosergestes curvatus* (Crosnier & Forest, 1973) | *Deosergestes corniculum* (Krøyer, 1855) | [3]^1^ |
| 2 | *Deosergestes erectus* (Burkenroad, 1940) | *Deosergestes coalitus* (Burkenroad, 1940) | [3]^1^ |
| 3 | *Deosergestes nipponensis* (Yokoya, 1933) | *Deosergestes seminudus* (Hansen, 1919) | [3]^1^ |
| 4 | *Lucifer* Thompson, 1829 [part] | *Belzebub* Vereshchaka, Olesen & Lunina, 2016 | [11] |
| 5 | *Lucifer chacei* Bowman, 1967 | *Belzebub* *chacei* (Bowman, 1967) | [11] |
| 6 | *Lucifer faxoni* Borradaile, 1915 | *Belzebub* *faxoni* (Borradaile, 1915) | [11] |
| 7 | *Lucifer hanseni* Nobili, 1905 | *Belzebub* *hanseni* (Nobili, 1905) | [11] |
| 8 | *Lucifer intermedius* Hansen, 1919 | *Belzebub* *intermedius* (Hansen, 1919) | [11] |
| 9 | *Lucifer penicillifer* Hansen, 1919 | *Belzebub* *penicillifer* (Hansen, 1919) | [11] |
| 10 | *Neosergestes geminus* (Judkins, 1978) | *Neosergestes orientalis* (Hansen, 1919) | [3]^1^ |
| 11 | *Neosergestes gibbilobatus* (Judkins, 1978) | *Neosergestes orientalis* (Hansen, 1919) | [3]^1^ |
| 12 | *Parasergestes extensus* (Hanamura, 1983) | *Parasergestes armatus* (Krøyer, 1855) | [3]^1^ |
| 13 | *Peisos* Burkenroad, 1945 | *Acetes* H. Milne Edwards, 1830 | [10] |
| 14 | *Peisos petrunkevitchi* Burkenroad, 1945 | *Acetes petrunkevitchi* (Burkenroad, 1945) | [10] |
| 16 | *Sergestes cornutus* Krøyer, 1855 | *Cornutosergestes cornutus* (Krøyer, 1855) | [4] |
| 17 | *Sergestes hamifer* Alcock & Anderson, 1894 | *Parasergestes armatus* (Krøyer, 1855) | **New synonym**, based upon original description and fugures |
| 19 | *Sergia erythraeensis* Iwasaki & Couwelaar, 2001 | *Lucensosergia colosii* (Cecchini, 1933) | **New synonym**, based upon original description and fugures |
| 20 | *Sergia manningorum* Froglia & Gramitto | *Robustosergia robusta* (Smith, 1882) | **New synonym**, based upon original description and fugures |
| 21 | *Sergia japonica* (Bate, 1881) | *Sergia remipes* Stimpson, 1860 | **New synonym**, based upon original description |

^1^ - first synonymy included this species in the genus *Sergestes* H. Milne Edwards, 1830.
