# Supplementary material for: The shrimp superfamily Sergestoidea: a global phylogeny with definition of new families and an assessment of the pathways into principal biotopes
Source: R Soc Open Sci. 2017 Sep 6;4(9):170221. doi: 10.1098/rsos.170221 (PMC5627073; doi:10.1098/rsos.170221)
Supplement: Appendix 3 [file rsos170221supp3.doc]

Appendix 3. List of characters used.

| Charac-ter No | Character state | State No | Reference to figure and source |
| --- | --- | --- | --- |
| **CARAPACE** | | | |
| 0 | Integument firm | 0 |  |
|  | Integument membranous^1^ | 1 |  |
| 1 | Labrum not much separated from antennae and eyes | 0 |  |
|  | Labrum widely separated from antennae and eyes | 1 |  |
| 2 | Rostrum bears 2or more dorsal teeth behind the orbital margin | 0 |  |
|  | Rostrum bears 0-1 dorsal teeth behind the orbital margin^2^ | 1 |  |
| 3 | Frontal margin of rostrum oblique | 0 | 2B,D – Vereshchaka *et al*. (2014) |
|  | Frontal margin of rostrum vertical | 1 | 2A – Vereshchaka *et al*. (2014) |
| 4 | Supraorbital tooth absent | 0 | 2A-C – Vereshchaka *et al*. (2014) |
|  | Supraorbital tooth present | 1 | 2D – Vereshchaka *et al*. (2014) |
| 5 | Pterygostomial tooth absent | 0 |  |
|  | Pterygostomial tooth present | 1 |  |
| 6 | Hepatic protrusion prominent | 0 |  |
|  | Hepatic protrusion inconspicuous | 1 |  |
| 7 | Hepatic spine absent | 0 | 2A-C – Vereshchaka *et al*. (2014) |
|  | Hepatic present | 1 | 2D – Vereshchaka *et al*. (2014) |
| 8 | Hepatic barb absent | 0 | 2A-C – Vereshchaka *et al*. (2014) |
|  | Hepatic barb present | 1 | 2D – Vereshchaka *et al*. (2014) |
| **BRANCHS** | | | |
| 9 | Somite VIII, arthrobranch developed | 0 |  |
|  | Somite VIII, arthrobranch rudimentary or absent^2^ | 1 |  |
| 10 | Somite VIII, arthrobranch absent | 0 |  |
|  | Somite VIII, arthrobranch present | 1 |  |
| 11 | Somite IX, anterior arthrobranch present | 0 |  |
|  | Somite IX, anterior arthrobranch absent | 1 |  |
| 12 | Somite IX, posterior arthrobranch present | 0 |  |
|  | Somite IX, posterior arthrobranch absent | 1 |  |
| 13 | Somite IX, posterior arthrobranch developed | 0 |  |
|  | Somite IX, posterior arthrobranch reduced | 1 |  |
| 14 | Somite IX, posterior arthrobranch dendritic | 0 |  |
|  | Somite IX, posterior arthrobranch lamellar | 1 |  |
| 15 | Somite X, anterior arthrobranch present | 0 |  |
|  | Somite X, anterior arthrobranch absent | 1 |  |
| 16 | Somite X, posterior arthrobranch present | 0 |  |
|  | Somite X, posterior arthrobranch absent | 1 |  |
| 17 | Somite X, posterior arthrobranch developed | 0 |  |
|  | Somite X, posterior arthrobranch reduced | 1 |  |
| 18 | Somite X, posterior arthrobranch dendritic | 0 |  |
|  | Somite X, posterior arthrobranch lamellar | 1 |  |
| 19 | Somite XI, anterior arthrobranch present | 0 |  |
|  | Somite XI, anterior arthrobranch absent | 1 |  |
| 20 | Somite XI, posterior arthrobranch present | 0 |  |
|  | Somite XI, posterior arthrobranch absent | 1 |  |
| 21 | Somite XI, posterior arthrobranch developed | 0 |  |
|  | Somite XI, posterior arthrobranch reduced | 1 |  |
| 22 | Somite XI, posterior arthrobranch dendritic | 0 |  |
|  | Somite XI, posterior arthrobranch lamellar | 1 |  |
| 23 | Somite XII, anterior arthrobranch present | 0 |  |
|  | Somite XII, anterior arthrobranch absent | 1 |  |
| 24 | Somite XII, developed anterior arthrobranch present | 0 |  |
|  | Somite XII, developed anterior arthrobranch absent | 1 |  |
| 25 | Somite XII, rudimentary anterior arthrobranch present | 0 |  |
|  | Somite XII, rudimentary anterior arthrobranch absent | 1 |  |
| 26 | Somite XII, posterior arthrobranch present | 0 |  |
|  | Somite XII, posterior arthrobranch absent | 1 |  |
| 27 | Somite XII, reduced posterior arthrobranch present | 0 |  |
|  | Somite XII, reduced posterior arthrobranch absent | 1 |  |
| 28 | Somite XII, rudimentary dendritic posterior arthrobranch present | 0 |  |
|  | Somite XII, rudimentary dendritic posterior arthrobranch absent | 1 |  |
| 29 | Somite XII, rudimentary lamellar posterior arthrobranch present | 0 |  |
|  | Somite XII, rudimentary lamellar posterior arthrobranch absent | 1 |  |
| 30 | Somite XIII, anterior arthrobranch present | 0 |  |
|  | Somite XIII, anterior arthrobranch absent | 1 |  |
| 31 | Somite XIII, posterior arthrobranch present | 0 |  |
|  | Somite XIII, posterior arthrobranch absent | 1 |  |
| 32 | Somite XIII, posterior arthrobranch developed | 0 |  |
|  | Somite XIII, posterior arthrobranch reduced | 1 |  |
| 33 | Somite XIII, posterior arthrobranch dendritic | 0 |  |
|  | Somite XIII, posterior arthrobranch lamellar | 1 |  |
| **ABDOMEN AND TELSON** | | | |
| 34 | Somite VI in male without ventral processes | 0 | 1 – Vereshchaka *et al*. (2016) |
|  | Somite VI in male with two ventral processes | 1 | 1 – Vereshchaka *et al*. (2016) |
| 35 | Somite VI in male without not tapering, obtuse posterior ventral process | 0 | 1 – Vereshchaka *et al*. (2016) |
|  | Somite VI in male with not tapering, obtuse posterior ventral process | 1 | 1 – Vereshchaka *et al*. (2016) |
| 36 | Somite VI in male without tapering, acute posterior ventral process^3^ | 0 | 1 – Vereshchaka *et al*. (2016) |
|  | Somite VI in male with tapering, acute posterior ventral process | 1 | 1 – Vereshchaka *et al*. (2016) |
| 37 | Somite VI in male without straight posterior ventral process^3^ | 0 | 1 – Vereshchaka *et al*. (2016) |
|  | Somite VI in male with straight posterior ventral process | 1 | 1 – Vereshchaka *et al*. (2016) |
| 38 | Somite VI in male without curved posterior ventral process^3^ | 0 | 1 – Vereshchaka *et al*. (2016) |
|  | Somite VI in male with curved posterior ventral process | 1 | 1 – Vereshchaka *et al*. (2016) |
| 39 | Telson, lateral spines present | 0 |  |
|  | Telson, lateral spines absent | 1 |  |
| 40 | Telson without four movable lateral spines | 0 |  |
|  | Telson with four movable lateral spines | 1 |  |
| 41 | Telson without two movable lateral spines | 0 |  |
|  | Telson with two movable lateral spines | 1 |  |
| 42 | Telson in male straight distoventrally | 0 | 1 – Vereshchaka *et al*. (2016) |
|  | Telson in male with conspicuous distoventral protuberance | 1 | 1 – Vereshchaka *et al*. (2016) |
| **EYE** | | | |
| 43 | Eyes not sexually dimorphic | 0 |  |
|  | Eyes sexually dimorphic, larger in males | 1 |  |
| 44 | Eyestalks respectively short, not reaching end of scaphocerite | 0 |  |
|  | Eyestalks elongated, nearly reaching end of scaphocerite | 1 |  |
| **ANTENNULE** | | | |
| 45 | First segment respectively short (less than by half or more longer than 3rd segment) | 0 |  |
|  | First segment elongated (by half or more longer than 3rd segment) | 1 | 2E – Vereshchaka *et al*. (2014) |
| 46 | Third segment respectively short (less than by half or more longer than 3rd segment) | 0 |  |
|  | Third segment elongated (by half or more longer than 3rd segment) | 1 | 2F – Vereshchaka *et al*. (2014) |
| 47 | Third segment distoventrally unarmed in male | 0 |  |
|  | Third segment with distoventral processus in male | 1 | 2G – Vereshchaka *et al*. (2014) |
| 48 | Antennules in male with ventral flagellum | 0 |  |
|  | Antennules in male without ventral flagellum | 1 |  |
| 49 | Mobile stylocerite absent | 0 |  |
|  | Mobile stylocerite present | 1 |  |
| 50 | Fixed stylocerite absent | 0 |  |
|  | Fixed stylocerite present | 1 |  |
| **ANTENNA** | | | |
| 51 | Distal tooth of scaphocerite not reaching distal end of blade | 0 |  |
|  | Distal tooth of scaphocerite reaching distal end of blade | 1 | 7G – Vereshchaka *et al*. (2014) |
|  | Distal tooth of scaphocerite overreaching distal end of blade | 2 | 7E – Vereshchaka *et al*. (2014) |
| **MANDIBLE** | | | |
| 52 | Mandibular palp present | 0 |  |
|  | Mandibular palp absent | 1 |  |
| **MAXILLULE** | | | |
| 53 | Four endites absent in adults | 0 |  |
|  | Four endites present in adults | 1 |  |
| 54 | Three endites absent in adults | 0 |  |
|  | Three endites present in adults | 1 |  |
| 55 | Two endites absent in adults | 0 |  |
|  | Two endites present in adults | 1 |  |
| 56 | A single endite absent in adults | 0 |  |
|  | A single endite present in adults | 1 |  |
| **FIRST MAXILLIPED** | | | |
| 57 | Epipod present | 0 |  |
|  | Epipod absent | 1 |  |
| 58 | Exopod present | 0 |  |
|  | Exopod absent | 1 |  |
| 59 | Endopod well-developed | 0 |  |
|  | Endopod rudimentary/absent | 1 |  |
| 60 | Reduced 2-segmented endopod absent | 0 |  |
|  | Reduced 2-segmented endopod present | 1 |  |
| **SECOND MAXILLIPED** | | | |
| 61 | Epipod present | 0 |  |
|  | Epipod absent | 1 |  |
| **THIRD MAXILLIPED** | | | |
| 62 | Moderately developed, < 2.0 times as long as first pereopod | 0 | 2E – Vereshchaka *et al*. (2014) |
|  | Enlarged, > 2.0 times as long as first pereopod | 1 | 2F – Vereshchaka *et al*. (2014) |
| 63 | Not sexually dimorphic, dactyl not modified | 0 | 3D – Vereshchaka *et al*. (2014) |
|  | Sexually dimorphic, dactyl with oval subsegments in males | 1 | 3E – Vereshchaka *et al*. (2014) |
| 64 | Dactyl entire | 0 |  |
|  | Dactyl subdivided | 1 |  |
| 65 | Dactyl subdivided into ordinary subsegments absent | 0 |  |
|  | Dactyl subdivided into ordinary subsegments present | 1 | 2E – Vereshchaka *et al*. (2014) |
| 66 | Dactyl subdivided into specialized subsegments absent | 0 |  |
|  | Dactyl subdivided into specialized subsegments present | 1 | 2F – Vereshchaka *et al*. (2014) |
| 67 | Dactyl with four specialized subsegments absent | 0 |  |
|  | Dactyl with four specialized subsegments present | 1 | 6 – Vereshchaka (2009) |
| 68 | Dactyl with five specialized subsegments absent | 0 |  |
|  | Dactyl with five specialized subsegments present | 1 | 6 – Vereshchaka (2009) |
| 69 | Dactyl with six specialized subsegments absent | 0 |  |
|  | Dactyl with six specialized subsegments present | 1 | 6 – Vereshchaka (2009) |
| 70 | Dactyl with seven specialized subsegments absent | 0 |  |
|  | Dactyl with seven specialized subsegments present | 1 | 6 – Vereshchaka (2009) |
| **FIRST PEREOPOD** | | | |
| 71 | Ischium nearly smooth | 0 |  |
|  | Ischium with strong movable spines | 1 | 3F – Vereshchaka *et al*. (2014) |
| 72 | Chela absent | 0 |  |
|  | Chela present | 1 |  |
| 73 | Normal chela (palm nearly as long as fingers) absent | 0 |  |
|  | Normal chela (palm nearly as long as fingers) present | 1 |  |
| 74 | Much reduced chela ( palm >10 times as long as fingers) absent | 0 |  |
|  | Much reduced chela ( palm >10 times as long as fingers) present | 1 |  |
| **SECOND PEREOPOD** | | | |
| 75 | Ischium nearly smooth | 0 |  |
|  | Ischium with strong distally curved tooth | 1 | 3G – Vereshchaka *et al*. (2014) |
| 76 | Merus distally unarmed | 0 |  |
|  | Merus with distal protrusion | 1 | 3G – Vereshchaka *et al*. (2014) |
| 77 | Chela present | 0 |  |
|  | Chela absent | 1 |  |
| 78 | Slightly reduced chela (palm twice as long as fingers) absent | 0 |  |
|  | Slightly reduced chela (palm twice as long as fingers) present | 1 |  |
| 79 | Much reduced chela ( palm >10 times as long as fingers) absent | 0 |  |
|  | Much reduced chela ( palm >10 times as long as fingers) present | 1 |  |
| 80 | Chela without rudimentary fixed finger, which is shorter then dactyl | 0 |  |
|  | Chela with rudimentary fixed finger, which is shorter then dactyl | 1 | 3H – Vereshchaka *et al*. (2014) |
| 81 | Chela without well-developed fixed finger, which is as long as then dactyl | 0 |  |
|  | Chela with well-developed fixed finger, which is as long as then dactyl | 1 | 3I – Vereshchaka *et al*. (2014) |
| 82 | Chela with short setae not overreaching setae in tufts | 0 |  |
|  | Chela with very long setae overreaching setae in tufts | 1 | 3I – Vereshchaka *et al*. (2014) |
| **THIRD PEREOPOD** | | | |
| 83 | Coxa, mesial tooth absent | 0 |  |
|  | Coxa, mesial tooth present | 1 |  |
| 84 | Basis in female rounded | 0 |  |
|  | Basis in female with small projection or tooth | 1 |  |
| 85 | Propodus without specialized strong curved spines proximal to tufts of setae | 0 |  |
|  | Propodus with specialized strong curved spines proximal to tufts of setae | 1 | 3J – Vereshchaka *et al*. (2014) |
| 86 | True chela | 0 |  |
|  | Subchela | 1 | 3I – Vereshchaka *et al*. (2014) |
| 87 | Slightly reduced chela (palm 3 times as long as fingers) absent | 0 |  |
|  | Slightly reduced chela (palm 3 times as long as fingers) present | 1 |  |
| 88 | Much reduced chela ( palm >10 times as long as fingers) absent | 0 |  |
|  | Much reduced chela ( palm >10 times as long as fingers) present | 1 |  |
| 89 | Chela without rudimentary fixed finger, which is shorter then dactyl | 0 |  |
|  | Chela with rudimentary fixed finger, which is shorter then dactyl | 1 | 3J – Vereshchaka *et al*. (2014) |
| 90 | Chela without well-developed fixed finger, which is as long as then dactyl | 0 |  |
|  | Chela with well-developed fixed finger, which is as long as then dactyl | 1 | 3K– Vereshchaka *et al*. (2014) |
| 91 | Chela without very long setae overreaching setae in tufts | 0 |  |
|  | Chela with very long setae overreaching setae in tufts | 1 | 3J – Vereshchaka *et al*. (2014) |
| **FOURTH PEREOPOD** | | | |
| 92 | Present in female | 0 |  |
|  | Absent in female | 1 |  |
| 93 | Female without 7-segmented fourth pereopod | 0 |  |
|  | Female with 7-segmented fourth pereopod | 1 |  |
| 94 | Female without 6-segmented fourth pereopod | 0 |  |
|  | Female with 6-segmented fourth pereopod | 1 |  |
| 95 | Female without 5-segmented fourth pereopod | 0 |  |
|  | Female with 5-segmented fourth pereopod | 1 |  |
| 96 | Present in male | 0 |  |
|  | Absent in male | 1 |  |
| 97 | Male without 7-segmented fourth pereopod | 0 |  |
|  | Male with 7-segmented fourth pereopod | 1 |  |
| 98 | Male without 6-segmented fourth pereopod | 0 |  |
|  | Male with 6-segmented fourth pereopod | 1 |  |
| 99 | Male without 3-segmented fourth pereopod | 0 |  |
|  | Male with 3-segmented fourth pereopod | 1 |  |
| 100 | Carpus and propodus setose along one margin absent | 0 |  |
|  | Carpus and propodus setose along one margin present | 1 |  |
| 101 | Carpus and propodus setose along both margins absent | 0 |  |
|  | Carpus and propodus setose along both margins present | 1 |  |
| **FIFTH PEREOPOD** | | | |
| 102 | Present in female | 0 |  |
|  | Absent in female | 1 |  |
| 103 | Female without 7-segmented fourth pereopod | 0 |  |
|  | Female with 7-segmented fourth pereopod | 1 |  |
| 104 | Female without 6-segmented fourth pereopod | 0 |  |
|  | Female with 6-segmented fourth pereopod | 1 |  |
| 105 | Female without 3-segmented fourth pereopod | 0 |  |
|  | Female with 3-segmented fourth pereopod | 1 |  |
| 106 | Present in male | 0 |  |
|  | Absent in male | 1 |  |
| 107 | Male without 7-segmented fourth pereopod | 0 |  |
|  | Male with 7-segmented fourth pereopod | 1 |  |
| 108 | Male without 6-segmented fourth pereopod | 0 |  |
|  | Male with 6-segmented fourth pereopod | 1 |  |
| 109 | Male without 1-segmented fourth pereopod | 0 |  |
|  | Male with 1-segmented fourth pereopod | 1 |  |
| 110 | Carpus and propodus setose along one margin absent | 0 |  |
|  | Carpus and propodus setose along one margin present | 1 |  |
| 111 | Carpus and propodus setose along both margins absent | 0 |  |
|  | Carpus and propodus setose along both margins present | 1 |  |
| **UROPODAL EXOPOD** | | | |
| 112 | Outer spine absent | 0 | 10 – Vereshchaka (2009) |
|  | Outer spine present | 1 | 10 – Vereshchaka (2009) |
| 113 | Outer margin of proximal segment naked | 0 | 10 – Vereshchaka (2009) |
|  | Outer margin of proximal segment setose | 1 | 10 – Vereshchaka (2009) |
| 114 | Outer margin of proximal segment not setose or setose entirely | 0 |  |
|  | Outer margin of proximal segment setose partly | 1 | 10 – Vereshchaka (2009) |
| 115 | Outer margin of proximal segment not setose or setose partly | 0 |  |
|  | Outer margin of proximal segment setose entirely | 1 | 10 – Vereshchaka (2009) |
| **MALE CLASPING ORGAN** | | | |
| 116 | Absent | 0 | 4B |
|  | Present | 1 | 4C |
| 117 | A single clasping tubercle absent | 0 |  |
|  | A single clasping tubercle present | 1 |  |
| 118 | Two clasping tubercles absent | 0 |  |
|  | Two clasping tubercles present | 1 |  |
| 119 | One rudimentary and one well-developed clasping tubercles absent | 0 |  |
|  | One rudimentary and one well-developed clasping tubercles present | 1 |  |
| 120 | Two well-developed clasping tubercles absent | 0 |  |
|  | Two well-developed clasping tubercles present | 1 |  |
| 121 | Claw-like setae positioned in scattered pairs opposite to the tubercle absent | 0 |  |
|  | Claw-like setae positioned in scattered pairs opposite to the tubercle present | 1 |  |
| 122 | No claw-like setae positioned in two continuous row with gap | 0 |  |
|  | Claw-like setae positioned in two continuous row with gap present | 1 |  |
| 123 | Serrated bristles opposite to the tubercle absent | 0 |  |
|  | Serrated bristles opposite to the tubercle present | 1 |  |
| 124 | Serrated bristles with reticulate distal part absent | 0 |  |
|  | Serrated bristles with reticulate distal part present | 1 |  |
| 125 | Serrated bristles with longitudinal ribs in distal part absent | 0 |  |
|  | Serrated bristles with longitudinal ribs in distal part present | 1 |  |
| 126 | Scales opposite to the tubercle absent | 0 |  |
|  | Scales opposite to the tubercle present | 1 |  |
| 127 | A row of serrated bristles adjacent to the tubercle absent | 0 |  |
|  | A row of serrated bristles adjacent to the tubercle present | 1 |  |
| 128 | Strong distal tooth on the fourth segment absent | 0 |  |
|  | Strong distal tooth on the fourth segment present | 1 |  |
| **PETASMA** | | | |
| 129 | Pars astrigens present | 0 |  |
|  | Pars astrigens absent | 1 |  |
| 130 | Well-developed pars astrigens present | 0 |  |
|  | Well-developed pars astrigens absent | 1 |  |
| 131 | Vestigial pars astrigens absent present | 0 |  |
|  | Vestigial pars astrigens present | 1 |  |
| 132 | Pars externa not transformed into a wide unsupported sheath | 0 |  |
|  | Pars externa transformed into a wide unsupported sheath | 1 |  |
| 133 | Pars externa not transformed into a narrow sheath supported by chitinous rib | 0 |  |
|  | Pars externa transformed into a narrow sheath supported by chitinous rib | 1 |  |
| 134 | Pars externa without hook | 0 | 5C, 6D – Vereshchaka *et al*. (2014) |
|  | Pars externa with a hook | 1 | 5A,B,D – Vereshchaka *et al*. (2014) |
| 135 | Pars externa without terminal setae | 0 |  |
|  | Pars externa with terminal setae | 1 |  |
| 136 | Pars externa unarmed at the tip | 0 |  |
|  | Pars externa with fine friction structures at the tip | 1 |  |
| 137 | Pars externa not armed with transverse ribs along entire margin | 0 |  |
|  | Pars externa armed with transverse ribs along entire margin | 1 |  |
| 138 | Pars externa without plate-like structures in addition to friction structures | 0 |  |
|  | Pars externa with plate-like structures in addition to friction structures | 1 |  |
| 139 | Capitulum absent or vestigial | 0 |  |
|  | Capitulum present | 1 |  |
| 140 | Capitulum not armed with strong claws | 0 |  |
|  | Capitulum armed with strong claws | 1 |  |
| 141 | Divided capitulum without with strong claws | 0 |  |
|  | Divided capitulum armed with strong claws | 1 |  |
| 142 | Capitulum without squamose hooks in addition to ordinary claws | 0 |  |
|  | Capitulum with squamose hooks in addition to ordinary claws | 1 |  |
| 143 | Capitulum without enlarged claws in addition to ordinary claws | 0 |  |
|  | Capitulum with enlarged claws in addition to ordinary claws | 1 |  |
| 144 | Capitulum not armed with squamose hooks and pincers | 0 |  |
|  | Capitulum armed with squamose hooks and pincers | 1 |  |
| 145 | No entire capitulum armed with squamose hooks and pincers | 0 |  |
|  | Entire capitulum armed with squamose hooks and pincers present | 1 |  |
| 146 | No divided capitulum armed with squamose hooks and pincers | 0 |  |
|  | Divided capitulum armed with squamose hooks and pincers present | 1 |  |
| 147 | Not modified capitulum with pincers (all lobi and processi present and not divided) absent | 0 |  |
|  | Not modified capitulum with pincers (all lobi and processi present and not divided) present | 1 |  |
| 148 | Significantly modified (divided or reduced) capitulum with pincers lobi/processi absent | 0 |  |
|  | Significantly modified (divided or reduced) capitulum with pincers lobi/processi present | 1 |  |
| 149 | Lobus armatus absent | 0 |  |
|  | Lobus armatus present | 1 |  |
| 150 | Rudimentary lobus armatus absent | 0 |  |
|  | Rudimentary lobus armatus present | 1 | 5E – Vereshchaka *et al*. (2014) |
| 151 | Well-developed lobus armatus absent | 0 |  |
|  | Well-developed lobus armatus present | 1 | 5A-D – Vereshchaka *et al*. (2014) |
| 152 | Lobus connectens and lobus terminalis not twisted | 0 | 5A-E – Vereshchaka *et al*. (2014) |
|  | Lobus connectens and lobus terminalis twisted | 1 | 6D – Vereshchaka *et al*. (2014) |
| 153 | Lobus connectens absent | 0 | 5B – Vereshchaka *et al*. (2014) |
|  | Lobus connectens present | 1 | 4A,C-E – Vereshchaka *et al*. (2014) |
| 154 | Rudimentary lobus connectens absent | 0 |  |
|  | Rudimentary lobus connectens present | 1 | 5C – Vereshchaka *et al*. (2014) |
| 155 | Well-developed lobus connectens absent | 0 |  |
|  | Well-developed lobus connectens present | 1 | 5A,D,E – Vereshchaka *et al*. (2014) |
| 156 | Entire lobus connectens absent | 0 |  |
|  | Entire lobus connectens present | 1 | 5A,C,D – Vereshchaka *et al*. (2014) |
| 157 | Terminally divided lobus connectens absent | 0 |  |
|  | Terminally divided lobus connectens present | 1 | 5E – Vereshchaka *et al*. (2014) |
| 158 | Lobus connectens with additional lobe at base directed upward absent | 0 |  |
|  | Lobus connectens with additional lobe at base directed upward present | 1 | 6C – Vereshchaka *et al*. (2014) |
| 159 | Lobus connectens with additional lobe at base directed downward absent | 0 |  |
|  | Lobus connectens with additional lobe at base directed downward present | 1 |  |
| 160 | Lobus connectens not swan-shaped | 0 | 6B,C – Vereshchaka *et al*. (2014) |
|  | Lobus connectens swan-shaped | 1 | 6E – Vereshchaka *et al*. (2014) |
| 161 | Lobus connectens without pillow at base | 0 | 6C,D – Vereshchaka *et al*. (2014) |
|  | Lobus connectens with pillow at base | 1 | 6E – Vereshchaka *et al*. (2014) |
| 162 | Apex of lobus connectens not bearing a single much enlarged sucker with a hook inside | 0 | 5D,E – Vereshchaka *et al*. (2014) |
|  | Apex of lobus connectens bearing a single much enlarged sucker with a hook inside | 1 | 6E – Vereshchaka *et al*. (2014) |
| 163 | Curved lobus inermis absent | 0 |  |
|  | Curved lobus inermis present | 1 | 6A – Vereshchaka *et al*. (2014) |
| 164 | Straight lobus inermis absent | 0 |  |
|  | Straight lobus inermis present | 1 | 5A,B,E – Vereshchaka *et al*. (2014) |
| 165 | Inflated lobus inermis absent | 0 |  |
|  | Inflated lobus inermis present | 1 | 6A – Vereshchaka *et al*. (2014) |
| 166 | Narrow lobus inermis absent | 0 |  |
|  | Narrow lobus inermis present | 1 | 5B – Vereshchaka *et al*. (2014) |
| 167 | Lobus terminalis absent or rudimentary | 0 |  |
|  | Lobus terminalis developed | 1 | 5A-D – Vereshchaka *et al*. (2014) |
| 168 | Entire lobus terminalis absent | 0 |  |
|  | Entire lobus terminalis present | 1 | 5A-D – Vereshchaka *et al*. (2014) |
| 169 | Terminally divided lobus terminalis absent | 0 |  |
|  | Terminally divided lobus terminalis present | 1 | 6A – Vereshchaka *et al*. (2014) |
| 170 | Lobus terminalis with additional lobe at base absent | 0 |  |
|  | Lobus terminalis with additional lobe at base present | 1 | 1C, 3C – Vereshchaka *et al*. (2014) |
| 171 | Processus ventralis absent | 0 | 5C – Vereshchaka *et al*. (2014) |
|  | Processus ventralis present | 1 | 5A,B,D, – Vereshchaka *et al*. (2014) |
| 172 | Rudimentary processus ventralis absent | 0 |  |
|  | Rudimentary processus ventralis present | 1 | 5E – Vereshchaka *et al*. (2014) |
| 173 | Developed rocessus ventralis absent | 0 |  |
|  | Developed rocessus ventralis present | 1 | 5A,B,D – Vereshchaka *et al*. (2014) |
| 174 | Processus ventralis without lateral friction structures | 0 |  |
|  | Processus ventralis with lateral friction structures | 1 |  |
| 175 | Processus ventralis without minute apical setae | 0 |  |
|  | Processus ventralis with minute apical setae | 1 |  |
| 176 | Entire processus ventralis absent | 0 |  |
|  | Entire processus ventralis present | 1 | 5A,B,D, – Vereshchaka *et al*. (2014) |
| 177 | Divided processus ventralis absent | 0 |  |
|  | Divided processus ventralis present | 1 | 2C, 4C – present paper |
| 178 | Twice divided processus ventralis absent | 0 |  |
|  | Twice divided processus ventralis present | 1 |  |
| 179 | Entire elongate processus ventralis absent | 0 |  |
|  | Entire elongate processus ventralis present | 1 | 5A,B,D – Vereshchaka *et al*. (2014) |
| 180 | Triangle processus ventralis absent | 0 |  |
|  | Triangle processus ventralis present | 1 | 5E – Vereshchaka *et al*. (2014) |
| 181 | Needle-like elongate processus ventralis absent | 0 |  |
|  | Needle-like elongate processus ventralis present | 1 | 5E – Vereshchaka *et al*. (2014) |
| 182 | Processus ventralis without hooks and sucks | 0 | 5A-E – Vereshchaka *et al*. (2014) |
|  | Processus ventralis with hooks and sucks | 1 | 6B – Vereshchaka *et al*. (2014) |
| 183 | Processus ventralis without simple spines | 0 | 5A,C,E – Vereshchaka *et al*. (2014) |
|  | Processus ventralis with simple spines | 1 | 5B,D – Vereshchaka *et al*. (2014) |
| 184 | Processus ventralis without simple spines | 0 |  |
|  | Processus ventralis with simple spines | 1 |  |
| 185 | Processus ventralis without >4 simple spines | 0 |  |
|  | Processus ventralis with >4 simple spines | 1 |  |
| 186 | Processus ventralis without stellate spines | 0 | 5A,D,E – Vereshchaka *et al*. (2014) |
|  | Processus ventralis with stellate spines | 1 | 5B – Vereshchaka *et al*. (2014) |
| 187 | Processus ventralis without apical lashes | 0 | 5B-E – Vereshchaka *et al*. (2014) |
|  | Processus ventralis with apical lashes | 1 | 5A– Vereshchaka *et al*. (2014) |
| 188 | Processus ventralis without 5-25 apical lashes in row | 0 |  |
|  | Processus ventralis with 5-25 apical lashes in row | 1 | 5A– Vereshchaka *et al*. (2014) |
| 189 | Processus ventralis without two pincer-like apical lashes | 0 |  |
|  | Processus ventralis with two pincer-like apical lashes | 1 | 5A– Vereshchaka *et al*. (2014) |
| **PHOTOPHORES** | | | |
| 190 | The organ of Pesta absent | 0 | 2F – Vereshchaka *et al*. (2014) |
|  | The organ of Pesta present | 1 | 2E – Vereshchaka *et al*. (2014) |
| 191 | No organ of Pesta with anterolateral spheroid organs | 0 |  |
|  | The organ of Pesta with anterolateral spheroid organs present | 1 | 12 – Vereshchaka (2009) |
| 192 | No organ of Pesta with anterolateral lobed organ | 0 |  |
|  | The organ of Pesta with anterolateral lobed organ present | 1 | 12 – Vereshchaka (2009) |
| 193 | No organ of Pesta with posterolateral spheroid organs | 0 |  |
|  | The organ of Pesta with posterolateral spheroid organs present | 1 | 12 – Vereshchaka (2009) |
| 194 | No organ of Pesta with posterolateral fringed organs | 0 |  |
|  | The organ of Pesta with posterolateral fringed organs present | 1 | 12 – Vereshchaka (2009) |
| 195 | No organ of Pesta with 3 distinct organs (2 lateral midgastric, and 1 continuous posterior fringe) | 0 |  |
|  | The organ of Pesta with 3 distinct organs (2 lateral midgastric, and 1 continuous posterior fringe) present | 1 | 12 – Vereshchaka (2009) |
| 196 | No organ of Pesta with 6-8 distinct organs (1 anteromedian, 2 lateral midgastric, 2 posterolateral fringes) | 0 |  |
|  | The organ of Pesta with 6-8 distinct organs (1 anteromedian, 2 lateral midgastric, 2 posterolateral fringes) present | 1 | 12 – Vereshchaka (2009) |
| 197 | No organ of Pesta with 1 distinct posteromedian organ | 0 |  |
|  | The organ of Pesta with 1 distinct posteromedian organ present | 1 | 12 – Vereshchaka (2009) |
| 198 | No organ of Pesta with : 2-3 distinct organs (2 posterolateral and, in some species, 1 posteromedian) | 0 |  |
|  | The organ of Pesta with : 2-3 distinct organs (2 posterolateral and, in some species, 1 posteromedian) present | 1 | 12 – Vereshchaka (2009) |
| 199 | No organ of Pesta with : 3 distinct organs (1 anteromedian, and 2 posterolateral) | 0 |  |
|  | The organ of Pesta with : 3 distinct organs (1 anteromedian, and 2 posterolateral) present | 1 | 12 – Vereshchaka (2009) |
| 200 | No organ of Pesta with 4 distinct organs (1 anteromedian, 2 posterolateral, and 1 posteromedian) | 0 |  |
|  | The organ of Pesta with 4 distinct organs (1 anteromedian, 2 posterolateral, and 1 posteromedian) present | 1 | 12 – Vereshchaka (2009) |
| 201 | No organ of Pesta with 5 distinct organs (1 anteromedian, 2 lateral midgastric, and 2 posterolateral) | 0 |  |
|  | The organ of Pesta with 5 distinct organs (1 anteromedian, 2 lateral midgastric, and 2 posterolateral) present | 1 | 12 – Vereshchaka (2009) |
| 202 | Dermal photophores absent | 0 |  |
|  | Dermal photophores present | 1 |  |
| 203 | Lens-less dermal photophores absent | 0 |  |
|  | Lens-less dermal photophores present | 1 | 7C-F – Vereshchaka *et al*. (2014) |
| 204 | Lens-bearing dermal photophores absent | 0 |  |
|  | Lens- bearing dermal photophores present | 1 | 7A,B – Vereshchaka *et al*. (2014) |
| 205 | A total of 130-170 photophores absent | 0 |  |
|  | A total of 130-170 photophores present | 1 |  |
| 206 | A total of 190-210 photophores absent | 0 |  |
|  | A total of 190-210 photophores present | 1 |  |
| 207 | A total of 225 or more photophores absent | 0 |  |
|  | A total of 225 or more photophores present | 1 |  |
| 208 | Two lateral rows of photophores on carapace absent | 0 |  |
|  | Two lateral rows of photophores on carapace present | 1 | 7B – Vereshchaka *et al*. (2014) |
| 209 | A single lateral row of photophores on carapace absent | 0 |  |
|  | A single lateral row of photophores on carapace present | 1 | 7A – Vereshchaka *et al*. (2014) |
| 210 | Fixed number of photophores in the upper row on carapace absent | 0 |  |
|  | Fixed number of photophores in the upper row on carapace present | 1 |  |
| 211 | Not fixed number of photophores in the upper row on carapace absent | 0 |  |
|  | Not fixed number of photophores in the upper row on carapace present | 1 |  |
| 212 | Four or more photophores in the upper row on carapace absent | 0 |  |
|  | Four or more photophores in the upper row on carapace present | 1 | 7A,B – Vereshchaka *et al*. (2014) |
| 213 | Two-three photophores in the upper row on carapace absent | 0 |  |
|  | Two-three photophores in the upper row on carapace present | 1 |  |
| 214 | Not fixed number of photophores on scaphocerite absent | 0 |  |
|  | Not fixed number of photophores on scaphocerite present | 1 |  |
| 215 | Fixed number of photophores on scaphocerite absent | 0 |  |
|  | Fixed number of on scaphocerite present | 1 |  |
| 216 | Eight or more photophores on scaphocerite absent | 0 |  |
|  | Eight or more photophores on scaphocerite present | 1 | 7C,E – Vereshchaka *et al*. (2014) |
| 217 | Seven photophores on scaphocerite absent | 0 |  |
|  | Seven photophores on scaphocerite present | 1 |  |
| 218 | Four to six photophores on scaphocerite absent | 0 |  |
|  | Four to six photophores on scaphocerite present | 1 | 7G – Vereshchaka *et al*. (2014) |
| 219 | Two or three photophores on scaphocerite absent | 0 |  |
|  | Two or three photophores on scaphocerite present | 1 |  |
| 220 | Small photophores on scaphocerite absent | 0 |  |
|  | Small photophores on scaphocerite present | 1 | 7E – Vereshchaka *et al*. (2014) |
| 221 | Medium-sized photophores on scaphocerite absent | 0 |  |
|  | Medium-sized photophores on scaphocerite present | 1 |  |
| 222 | Large photophores on scaphocerite absent | 0 |  |
|  | Large photophores on scaphocerite present | 1 | 7C – Vereshchaka *et al*. (2014) |
| 223 | Separated from each other photophores on scaphocerite absent | 0 |  |
|  | Separated from each other photophores on scaphocerite present | 1 | 7E – Vereshchaka *et al*. (2014) |
| 224 | Partly fused photophores on scaphocerite absent | 0 |  |
|  | Partly fused photophores on scaphocerite present | 1 | 7C – Vereshchaka *et al*. (2014) |
| 225 | Two rows of photophores on scaphocerite absent | 0 |  |
|  | Two rows of photophores on scaphocerite present | 1 | 7C – Vereshchaka *et al*. (2014) |
| 226 | A single row of photophores on scaphocerite absent | 0 |  |
|  | A single row of photophores on scaphocerite present | 1 | 7G,E – Vereshchaka *et al*. (2014) |
| 227 | A contiguous row of equally spaced from each other photophores on uropodal exopod absent | 0 |  |
|  | A contiguous row of equally spaced from each other photophores on uropodal exopod present | 1 | 7F – Vereshchaka *et al*. (2014) |
| 228 | Two groups of photophores separated by a gap on uropodal exopod absent | 0 |  |
|  | Two groups of photophores separated by a gap on uropodal exopod present | 1 | 7D,H – Vereshchaka *et al*. (2014) |
| 229 | Small photophores on uropodal exopod absent | 0 |  |
|  | Small photophores on uropodal exopod present | 1 | 7F,H – Vereshchaka *et al*. (2014) |
| 230 | Medium-sized photophores on uropodal exopod absent | 0 |  |
|  | Medium-sized photophores on uropodal exopod present | 1 |  |
| 231 | Large photophores on uropodal exopod absent | 0 |  |
|  | Large photophores on uropodal exopod present | 1 | 7D – Vereshchaka *et al*. (2014) |
| 232 | Partly fused photophores on uropodal exopod absent | 0 |  |
|  | Partly fused photophores on uropodal exopod present | 1 | 7D – Vereshchaka *et al*. (2014) |
| 233 | Separated from each other photophores on uropodal exopod absent | 0 |  |
|  | Separated from each other photophores on uropodal exopod present | 1 | 7F – Vereshchaka *et al*. (2014) |
| 234 | Photophores on basal segment of uropodal exopod positioned closer to central axis absent | 0 |  |
|  | Photophores on basal segment of uropodal exopod positioned closer to central axis present | 1 | 7D,H – Vereshchaka *et al*. (2014) |
| 235 | Photophores on basal segment of uropodal exopod positioned closer margin absent | 0 |  |
|  | Photophores on basal segment of uropodal exopod positioned closer to margin present | 1 | 7F – Vereshchaka *et al*. (2014) |
| 236 | Not fixed number of photophores on basal segment of uropodal exopod absent | 0 |  |
|  | Not fixed number of photophores on basal segment of uropodal exopod present | 1 |  |
| 237 | Fixed number of photophores on basal segment of uropodal exopod absent | 0 |  |
|  | Fixed number of on basal segment of uropodal exopod present | 1 |  |
| 238 | Three or more photophores on basal segment of uropodal exopod absent | 0 | 7F – Vereshchaka *et al*. (2014) |
|  | Three or more photophores on basal segment of uropodal exopod present | 1 |  |
| 239 | Two photophores on basal segment of uropodal exopod absent | 0 |  |
|  | Two photophores on basal segment of uropodal exopod present | 1 |  |
| 240 | A single photophore on basal segment of uropodal exopod absent | 0 |  |
|  | A single photophore on basal segment of uropodal exopod present | 1 | 7H – Vereshchaka *et al*. (2014) |
| 241 | Photophores on distal segment of uropodal exopod positioned closer to central axis absent | 0 |  |
|  | Photophores on distal segment of uropodal exopod positioned closer to central axis present | 1 | 7D,H – Vereshchaka *et al*. (2014) |
| 242 | Photophores on distal segment of uropodal exopod positioned closer margin absent | 0 |  |
|  | Photophores on distal segment of uropodal exopod positioned closer to margin present | 1 | 7F – Vereshchaka *et al*. (2014) |
| 243 | Two rows or triangle of photophores on distal segment of uropodal exopod absent | 0 |  |
|  | Two rows or triangle of photophores on distal segment of uropodal exopod present | 1 | 7D – Vereshchaka *et al*. (2014) |
| 244 | A single row of photophores on distal segment of uropodal exopod absent | 0 |  |
|  | A single row of photophores on distal segment of uropodal exopod present | 1 | 7F,H – Vereshchaka *et al*. (2014) |
| 245 | Not fixed number of photophores on distal segment of uropodal exopod absent | 0 |  |
|  | Not fixed number of photophores on distal segment of uropodal exopod present | 1 |  |
| 246 | Fixed number of photophores on distal segment of uropodal exopod absent | 0 |  |
|  | Fixed number of on distal segment of uropodal exopod present | 1 |  |
| 247 | Three or more photophores on distal segment of uropodal exopod absent | 0 | 7F – Vereshchaka *et al*. (2014) |
|  | Three or more photophores on distal segment of uropodal exopod present | 1 | 7F – Vereshchaka *et al*. (2014) |
| 248 | A single photophore on distal segment of uropodal exopod absent | 0 |  |
|  | A single photophore on distal segment of uropodal exopod present | 1 | 7H – Vereshchaka *et al*. (2014) |
| **LARVA** | | | |
| 249 | Metamorphosis of usual dendrbranchiate type | 0 |  |
|  | Metamorphosis elaborated, including elaphocaris and acanthosoma stages | 1 |  |
| 250 | Elaphocaris stage of *dohrni* type absent | 0 |  |
|  | Elaphocaris stage of *dohrni* type present | 1 | 2 – Vereshchaka (2009) |
| 251 | Elaphocaris stage of *hispida* type absent | 0 |  |
|  | Elaphocaris stage of *hispida* type present | 1 | 2 – Vereshchaka (2009) |
| 252 | Elaphocaris stage of *ortmanni* type absent | 0 |  |
|  | Elaphocaris stage of *ortmanni* type present | 1 | 2 – Vereshchaka (2009) |

^1^ - very thin and soft, in intact specimens with regular riffles.

^2^ - variations.
