# Supplementary material for: The shrimp superfamily Sergestoidea: a global phylogeny with definition of new families and an assessment of the pathways into principal biotopes
Source: R Soc Open Sci. 2017 Sep 6;4(9):170221. doi: 10.1098/rsos.170221 (PMC5627073; doi:10.1098/rsos.170221)
Supplement: Appendix 4 [file rsos170221supp4.doc]

Appendix 4. Data matrix. Missing data indicated by question marks (?); inapplicable data by hyphens (-)

Characters 0 - 49

0 5 10 15 20 25 30 35 40 45

| | | | | | | | | |

Gennadas parvus 10100010001000000000000100000000000000000000100000

Penaeus monodon 00000001001000000000000100000010000000100000100100

Lucifer typus 01101101010110011001001001000110010101001111100100

Lucifer orientalis 01101101010110011001001001000110010101001111100100

Belsebub intermedius 01101101010110011001001001000110011010001100100100

Belsebub penicillifer 01101101010110011001001001000110011010001100100100

Belsebub faxoni 01101101010110011001001001000110011010001100100100

Belsebub chacei 01101101010110011001001001000110011010001100100100

Belsebub hanseni 01101101010110011001001001000110011010001100100100

Acetes americanus 00001001010010001001000101000010000000100000010001

Acetes binghami 00101001010010001001000101000010000000100000010001

Acetes chinensis 00001001010010001001000101000010000000100000010001

Acetes erythraeus 00001001010010001001000101000010000000100000010001

Acetes indicus 00001001010010001001000101000010000000100000010001

Acetes intermedius 00001001010010001001000101000010000000100000010001

Acetes japonicus 00001001010010001001000101000010000000100000010001

Acetes johni 00001001010010001001000101000010000000100000010001

Acetes natalensis 00001001010010001001000101000010000000100000010001

Acetes serrulatus 00001001010010001001000101000010000000100000010001

Acetes sibogae 00001001010010001001000101000010000000100000010001

Acetes vulgaris 00001001010010001001000101000010000000100000010001

Acetes marinus 00001001010010001001000101000010000000100000010001

Acetes paraguayensis 00001001010010001001000101000010000000100000010001

Acetes petrunkevitchi 00001001010010001001000101000010000000100000010001

Sicyonella inermis 00001101010001000100100100110001000000010000100001

Sicyonella maldivensis 00001101010001000100100100110001000000010000100001

Sicyonella antennata 00001101010001000100100100110001000000010000100001

Petalidium obesum 10100000110000100010010010101000100000100000000001

Petalidium foliaceum 10100001010000100010010010101010100000100000000001

Petalidium suspiriosum 10100001010000100010010010101001000000100000000001

Sergia tenuiremis 10100010001000100010010100010001000000100000100010

Sergia inoa 10100010001000100010010100010001000000100000100010

Sergia remipes 10100010001000100010010100010001000000100000100010

Sergia laminata 10100010001000100010010100010001000000100000100010

Gardinerosergia bigemmea 00100000101000100010010100010001000000100000100010

Gardinerosergia gardneri 00100000101000100010010100010001000000100000100010

Gardinerosergia inequalis 00100000101000100010010100010001000000100000100010

Gardinerosergia kensleyi 00100000101000100010010100010001000000100000100010

Gardinerosergia splendens 00100000101000100010010100010001000000100000100010

Phorcosergia bisulcata 00100000101000100010010100010001000000100000100010

Phorcosergia burukovskii 00100000101000100010010100010001000000100000100010

Phorcosergia filicta 00100000101000100010010100010001000000100000100010

Phorcosergia grandis 00100000101000100010010100010001000000100000100010

Phorcosergia maxima 00100000101000100010010100010001000000100000100010

Phorcosergia phorca 00100000101000100010010100010001000000100000100010

Phorcosergia plumea 00100000101000100010010100010001000000100000100010

Phorcosergia potens 00100000101000100010010100010001000000100000100010

Phorcosergia wolffi 00100000101000100010010100010001000000100000100010

Robustosergia extenuata 00100000101000100010010100010001000000100000100010

Robustosergia regalis 00100000101000100010010100010001000000100000100010

Robustosergia robusta 00100000101000100010010100010001000000100000100010

Robustosergia vityazi 00100000101000100010010100010001000000100000100010

Prehensilosergia prehensilis 00100000101000100010010100010001000000100000100010

Scintillosergia scintillans 00100000101000100010010100010001000000100000100010

Challengerosergia challengeri 00100001001000100010010100010001000000100000101010

Challengerosergia fulgens 00100001001000100010010100010001000000100000101010

Challengerosergia hansjacobi 00100001001000100010010100010001000000100000100010

Challengerosergia jeppeseni 00100001001000100010010100010001000000100000100010

Challengerosergia oksanae 00100001001000100010010100010001000000100000101010

Challengerosergia stellata 00100001001000100010010100010001000000100000101010

Challengerosergia talismani 00100001001000100010010100010001000000100000100010

Challengerosergia umitakae 00100001001000100010010100010001000000100000100010

Lucensosergia crosnieri 00100001001000100010010100010001000000100000100010

Lucensosergia colosii 00100001001000100010010100010001000000100000100010

Lucensosergia foresti 00100001001000100010010100010001000000100000100010

Lucensosergia lucens 00100001001000100010010100010001000000100000100010

Deosergestes coalitus 00100001001000100010010100010001000000100000000010

Deosergestes corniculum 00100001001000100010010100010001000000100000000010

Deosergestes disjunctus 00101001001000100010010100010001000000100000000010

Deosergestes henseni 00101001001000100010010100010001000000100000000010

Deosergestes paraseminudus 00100001001000100010010100010001000000100000000010

Deosergestes pediforms 00100001001000100010010100010001000000100000000010

Deosergestes rubroguttatus 00100001001000100010010100010001000000100000000010

Deosergestes seminudus 00100001001000100010010100010001000000100000000010

Eusergestes arcticus 00101001001000100010010100010001000000100000100010

Eusergestes similis 00101001001000100010010100010001000000100000100010

Eusergestes antarcticus 00101001001000100010010100010001000000100000100010

Sergestes atlanticus 00101001001000100010010100101001000000100000000001

Cornutosergestes cornutus 00101001001000100010010100101001000000100000000001

Cornutosergestes mepae 00101001001000100010010100101001000000100000000001

Allosergestes index 00110001001000100010010100101001000000100000000000

Allosergestes nudus 00111001001000100010010100101001000000100000000000

Allosergestes oleseni 00110001001000100010010100101001000000100000000000

Allosergestes pectinatus 00111001001000100010010100101001000000100000000000

Allosergestes pestafer 00111001001000100010010100101001000000100000000000

Allosergestes sargassi 00110001001000100010010100101001000000100000000000

Allosergestes verpus 00110001001000100010010100101001000000100000000000

Allosergestes vinogradovi 00110001001000100010010100101001000000100000000000

Parasergestes armatus 00100001001000100010010100101001000000100000000000

Parasergestes cylindricus 00100001001000100010010100101001000000100000000000

Parasergestes diapontius 00100001001000100010010100101001000000100000000000

Parasergestes halia 00100001001000100010010100101001000000100000000000

Parasergestes sirenkoi 00100001001000100010010100101001000000100000000000

Parasergestes stimulator 00100001001000100010010100101001000000100000000000

Parasergestes vigilax 00100001001000100010010100101001000000100000000000

Neosergestes brevispinatus 00100001001000100010010100101001000000100000000010

Neosergestes consobrinus 00100001001000100010010100101001000000100000000010

Neosergestes edwardsi 00100001001000100010010100101001000000100000000010

Neosergestes orientalis 00100001001000100010010100101001000000100000000010

Neosergestes semissis 00100001001000100010010100101001000000100000000010

Neosergestes tantillus 00100001001000100010010100101001000000100000000010

Characters 50 - 99

50 55 60 65 70 75 80 85 90 95

| | | | | | | | | |

Gennadas parvus 10100000000000000000001000000010000000010010001000

Penaeus monodon 00100000000000000000001000000010000000010010001000

Lucifer typus 21001011101000000000010000100000001100101100010000

Lucifer orientalis 21001011101000000000010000100000001100101100010000

Belsebub intermedius 21001011101000000000010000100000001100101100010000

Belsebub penicillifer 21001011101000000000010000100000001100101100010000

Belsebub faxoni 21001011101000000000010000100000001100101100010000

Belsebub chacei 21001011101000000000010000100000001100101100010000

Belsebub hanseni 21001011101000000000010000100000001100101100010000

Acetes americanus 00000100100000100000000100001010100001010100010000

Acetes binghami 00000100100000100000000100001010100001010100010000

Acetes chinensis 00000100100000100000000100001010100001010100010000

Acetes erythraeus 00000100100000100000000100001010100001010100010000

Acetes indicus 00000100100000100000000100001010110001010100010000

Acetes intermedius 00000100100000100000000100001010110001010100010000

Acetes japonicus 00000100100000100000000100001010100001010100010000

Acetes johni 00000100100000100000000100001010100001010100010000

Acetes natalensis 00000100100000100000000100001010000001010100010000

Acetes serrulatus 00000100100000100000000100001010100001010100010000

Acetes sibogae 00000100100000100000000100001010110001010100010000

Acetes vulgaris 00000100100000100000000100001010100001010100010000

Acetes marinus 00000100100000100000000100001010100001010100010000

Acetes paraguayensis 00000100100000100000000100001010100001010100010000

Acetes petrunkevitchi 00000100100000100000000100001010000001010000100010

Sicyonella inermis 00001000000101011000001000010010000010010010001001

Sicyonella maldivensis 00001000000101011000001000010010000010010010001001

Sicyonella antennata 00001000000101011000001000010010000010010010001001

Petalidium obesum 0001000000000?????????????001???00?001???001000101

Petalidium foliaceum 0001000000000?????????????001???00?001???001000101

Petalidium suspiriosum 0001000000000?????????????001???00?001???001000101

Sergia tenuiremis 00100000000001100000010000001010100001010001000100

Sergia inoa 00100000000001100000010000001010100001010001000100

Sergia remipes 00100000000001100000010000001010100001010001000100

Sergia laminata 00100000000001100000010000001010100001010001000100

Gardinerosergia bigemmea 20100000000001100000010000001010100001010001000100

Gardinerosergia gardneri 20100000000001100000010000001010100001010001000100

Gardinerosergia inequalis 20100000000001100000010000001010100001010001000100

Gardinerosergia kensleyi 20100000000001100000010000001010100001010001000100

Gardinerosergia splendens 20100000000001100000010000001010100001010001000100

Phorcosergia bisulcata 00100000000001100000010000001010100001010001000100

Phorcosergia burukovskii 00100000000001100000010000001010100001010001000100

Phorcosergia filicta 00100000000001100000010000001010100001010001000100

Phorcosergia grandis 00100000000001100000010000001010100001010001000100

Phorcosergia maxima 00100000000001100000010000001010100001010001000100

Phorcosergia phorca 00100000000001100000010000001010100001010001000100

Phorcosergia plumea 00100000000001100000010000001010100001010001000100

Phorcosergia potens 00100000000001100000010000001010100001010001000100

Phorcosergia wolffi 00100000000001100000010000001010100001010001000100

Robustosergia extenuata 00100000000001100000010000001010100001010001000100

Robustosergia regalis 00100000000001100000010000001010100001010001000100

Robustosergia robusta 00100000000001100000010000001010100001010001000100

Robustosergia vityazi 00100000000001100000010000001010100001010001000100

Prehensilosergia prehensilis 10100000000001100000010000001010100001010001000100

Scintillosergia scintillans 10100000000001100000010000001010100001010001000100

Challengerosergia challengeri 10100000000001100000010000001010100001010001000100

Challengerosergia fulgens 10100000000001100000010000001010100001010001000100

Challengerosergia hansjacobi 10100000000001100000010000001010100001010001000100

Challengerosergia jeppeseni 10100000000001100000010000001010100001010001000100

Challengerosergia oksanae 10100000000001100000010000001010100001010001000100

Challengerosergia stellata 10100000000001100000010000001010100001010001000100

Challengerosergia talismani 10100000000001100000010000001010100001010001000100

Challengerosergia umitakae 10100000000001100000010000001010100001010001000100

Lucensosergia crosnieri 10100000000001100000010000001010100001010001000100

Lucensosergia colosii 10100000000001100000010000001010100001010001000100

Lucensosergia foresti 10100000000001100000010000001010100001010001000100

Lucensosergia lucens 10100000000001100000010000001010100001010001000100

Deosergestes coalitus 10100000000001010001010000001011101001101001000100

Deosergestes corniculum 10100000000001010010010000001011101001101001000100

Deosergestes disjunctus 10100000000001010011010000001011101001101001000100

Deosergestes henseni 10100000000001010010010000001011101001101001000100

Deosergestes paraseminudus 10100000000001010001010000001011101001101001000100

Deosergestes pediforms 10100000000001010001010000001011101001101001000100

Deosergestes rubroguttatus 10100000000001010001010000001011101001101001000100

Deosergestes seminudus 10100000000001010001010000001011101001101001000100

Eusergestes arcticus 10100000000011010010010000001010100001010001000101

Eusergestes similis 10100000000011010010010000001010100001010001000101

Eusergestes antarcticus 10100000000011010010010000001010100001010001000101

Sergestes atlanticus 20100000000001010001010000001010000001010001000101

Cornutosergestes cornutus 20100000110001010001010000001010000001010001000101

Cornutosergestes mepae 20100000110001010001010000001010000001010001000101

Allosergestes index 10100000000101010100010000001100101001101001000100

Allosergestes nudus 10100000000101010010010000001100101001101001000100

Allosergestes oleseni 10100000000101010100010000001100101001101001000100

Allosergestes pectinatus 10100000000101010100010000001100101001101001000100

Allosergestes pestafer 10100000000101010100010000001100101001101001000100

Allosergestes sargassi 10100000000101010100010000001100101001101001000100

Allosergestes verpus 10100000000101010100010000001100101001101001000100

Allosergestes vinogradovi 10100000000101010100010000001100101001101001000100

Parasergestes armatus 20100000000101011000110010001010100001010001000101

Parasergestes cylindricus 20100000000101011000110010001010100001010001000101

Parasergestes diapontius 20100000000101011000110010001010100001010001000101

Parasergestes halia 20100000000101011000110010001010100001010001000101

Parasergestes sirenkoi 20100000000101011000110010001010100001010001000101

Parasergestes stimulator 20100000000101011000110010001010100001010001000101

Parasergestes vigilax 20100000000101011000110010001010100001010001000101

Neosergestes brevispinatus 20100000000101010010010011001010000001010001000101

Neosergestes consobrinus 20100000000101010010010011001010000001010001000101

Neosergestes edwardsi 20100000000101010010010011001010000001010001000101

Neosergestes orientalis 20100000000101010010010011001010000001010001000101

Neosergestes semissis 20100000000101010010010011001010000001010001000101

Neosergestes tantillus 20100000000101010010010011001010000001010001000101

Characters 100 - 149

100 105 110 115 120 125 130 135 140 145

| | | | | | | | | |

Gennadas parvus 00100010000100000000000000000000000000000000000000

Penaeus monodon 00100010000100000000000000000000000000000000000000

Lucifer typus 01000100000100000000000000001101010010100000000000

Lucifer orientalis 01000100000100000000000000001101000010100000000000

Belsebub intermedius 01000100000100000000000000001100101101100000000000

Belsebub penicillifer 01000100000100000000000000001100101101100000000000

Belsebub faxoni 01000100000100000000000000001100101101100000000000

Belsebub chacei 01000100000100000000000000001100101101100000000000

Belsebub hanseni 01000100000100000000000000001100101101100000000000

Acetes americanus 01000000100100011110001010011100000000111010000000

Acetes binghami 01000000100100011110001010011100000000111010000000

Acetes chinensis 01000000100100011101100000001100000000110100000000

Acetes erythraeus 01000000100100011110100000000000000000110101000000

Acetes indicus 01000000100100011110100000001100000000110100000000

Acetes intermedius 01000000100100011110100000000000000000110101000000

Acetes japonicus 01000000100100011101100000001100000000110100000000

Acetes johni 01000000100100011101100000011100000000111000000000

Acetes natalensis 01000000100100011101100000010010000000110100000000

Acetes serrulatus 01000000100100011101100000011100000000110101000000

Acetes sibogae 01000000100100011110100000000000000000110101000000

Acetes vulgaris 01000000100100011110100000000000000000110101000000

Acetes marinus 01000000100100011110001101010000000000000000000000

Acetes paraguayensis 01000000100100011110001101010000000000000000000000

Acetes petrunkevitchi 00001000100100011110001100010000010000111000000000

Sicyonella inermis 00100010010100010000000001000000000000100000110110

Sicyonella maldivensis 00100010010100010000010000000000000000100000101011

Sicyonella antennata 00100010010100010000010000000000000000100000101011

Petalidium obesum 00010001010100010000001101100000010000100000101011

Petalidium foliaceum 00010001010100010000001101100000010000100000101011

Petalidium suspiriosum 00010001010100010000001101100000010000100000101011

Sergia tenuiremis 10010001001100011000000001000000010000100000101101

Sergia inoa 10010001001100011000000001000000010000100000101101

Sergia remipes 10010001001100011000000001000000010000100000101101

Sergia laminata 10010001001100011000000001000000010000100000101101

Gardinerosergia bigemmea 10010001001100011000001101000000010000100000101011

Gardinerosergia gardneri 10010001001100011000001101000000010000100000101011

Gardinerosergia inequalis 10010001001100011000001101000000010000100000101011

Gardinerosergia kensleyi 10010001001100011000001101000000010000100000101011

Gardinerosergia splendens 10010001001100011000001101000000010000100000101011

Phorcosergia bisulcata 10010001001100011000001101000000010000100000101011

Phorcosergia burukovskii 10010001001100011000001101000000010000100000101011

Phorcosergia filicta 10010001001100011000001101000000010000100000101011

Phorcosergia grandis 10010001001100011000001101000000010000100000101011

Phorcosergia maxima 10010001001100011000001101000000010000100000101011

Phorcosergia phorca 10010001001100011000001101000000010000100000101011

Phorcosergia plumea 10010001001100011000001101000000010000100000101011

Phorcosergia potens 10010001001100011000001101000000010000100000101011

Phorcosergia wolffi 10010001001100011000001101000000010000100000101011

Robustosergia extenuata 10010001001100011000001101000000010000100000101011

Robustosergia regalis 10010001001100011000001101000000010000100000101011

Robustosergia robusta 10010001001100011000001101000000010000100000101011

Robustosergia vityazi 10010001001100011000001101000000010000100000101011

Prehensilosergia prehensilis 10010001001100011000001101000000010000100000101011

Scintillosergia scintillans 10010001001100011000001101000000010000100000101011

Challengerosergia challengeri 10010001001100011000001101000000010000100000101011

Challengerosergia fulgens 10010001001100011000001101000000010000100000101011

Challengerosergia hansjacobi 10010001001100011000001101000000010000100000101011

Challengerosergia jeppeseni 10010001001100011000001101000000010000100000101011

Challengerosergia oksanae 10010001001100010000001101000000010000100000101011

Challengerosergia stellata 10010001001100011000001101000000010000100000101011

Challengerosergia talismani 10010001001100010000001101000000010000100000101011

Challengerosergia umitakae 10010001001100011000001101000000010000100000101011

Lucensosergia crosnieri 10010001001100011000001101000000010000100000101010

Lucensosergia colosii 10010001001100011000001101000000010000100000101011

Lucensosergia foresti 10010001001100011000001101000000010000100000101011

Lucensosergia lucens 10010001001100011000001101000000010000100000101011

Deosergestes coalitus 10010001001011011000000001000000010000100000101011

Deosergestes corniculum 10010001001011011000000001000000010000100000101011

Deosergestes disjunctus 10010001001011011000000001000000010000100000101011

Deosergestes henseni 10010001001011011000000001000000010000100000101011

Deosergestes paraseminudus 10010001001011011000000001000000010000100000101011

Deosergestes pediforms 10010001001011011000000001000000010000100000101011

Deosergestes rubroguttatus 10010001001011011000000001000000010000100000101011

Deosergestes seminudus 10010001001011011000000001000000010000100000101011

Eusergestes arcticus 00010001010100011000000001000000010000100000101011

Eusergestes similis 00010001010100011000000001000000010000100000101011

Eusergestes antarcticus 00010001010100011000000001000000010000100000101011

Sergestes atlanticus 00010001010100011000000001000000000000100000101011

Cornutosergestes cornutus 00010001010100011000000001000000000000100000101011

Cornutosergestes mepae 00010001010100011000000001000000000000100000101011

Allosergestes index 10010001001011011000000001000000010000100000101011

Allosergestes nudus 10010001001011011000000001000000010000100000101011

Allosergestes oleseni 10010001001011010000000001000000010000100000101011

Allosergestes pectinatus 10010001001011011000000001000000010000100000101011

Allosergestes pestafer 10010001001011011000000001000000010000100000101011

Allosergestes sargassi 10010001001011011000000001000000010000100000101011

Allosergestes verpus 10010001001011011000000001000000010000100000101011

Allosergestes vinogradovi 10010001001011011000000001000000010000100000101011

Parasergestes armatus 00010001010011011000000001000000000000100000101011

Parasergestes cylindricus 00010001010011011000000001000000000000100000101011

Parasergestes diapontius 00010001010011011000000001000000000000100000101011

Parasergestes halia 00010001010011011000000001000000000000100000101011

Parasergestes sirenkoi 00010001010011011000000001000000000000100000101011

Parasergestes stimulator 00010001010011011000000001000000000000100000101011

Parasergestes vigilax 00010001010011011000000001000000000000100000101011

Neosergestes brevispinatus 00010001010010111000000001000000000000100000101011

Neosergestes consobrinus 00010001010010111000000001000000000000100000101011

Neosergestes edwardsi 00010001010010111000000001000000000000100000101011

Neosergestes orientalis 00010001010010111000000001000000000000100000101011

Neosergestes semissis 00010001010010111000000001000000000000100000101011

Neosergestes tantillus 00010001010010111000000001000000000000100000101011

Characters 150 - 199

150 155 160 165 170 175 180 185 190 195

| | | | | | | | | |

Gennadas parvus 00000000000000000000000000000000000000000000000000

Penaeus monodon 00000000000000000000000000000000000000000000000000

Lucifer typus 00000000000000000000011000100100000001010000000000

Lucifer orientalis 00000000000000000000011000100100000001010000000000

Belsebub intermedius 00000000000000000000011000100001000000000000000000

Belsebub penicillifer 00000000000000000000011011100001000000000000000000

Belsebub faxoni 00000000000000000000011010100001000000000000000000

Belsebub chacei 00000000000000000000011010100001000000000000000000

Belsebub hanseni 00000000000000000000011010100001000000000000000000

Acetes americanus 00000000000000000000000000000000000000000000000000

Acetes binghami 00000000000000000000000000000000000000000000000000

Acetes chinensis 00000000000000000000011000100001000000000000000000

Acetes erythraeus 00000000000000000000011000100100000000000000000000

Acetes indicus 00000000000000000000011000100001000000000000000000

Acetes intermedius 00000000000000000000011000100100000000000000000000

Acetes japonicus 00000000000000000000011000100001000000000000000000

Acetes johni 00000000000000000000000000000000000000000000000000

Acetes natalensis 00000000000000000000011000100001000000000000000000

Acetes serrulatus 00000000000000000000000000000000000000000000000000

Acetes sibogae 00000000000000000000011000100100000000000000000000

Acetes vulgaris 00000000000000000000011000100100000000000000000000

Acetes marinus 00000000000000000000000000000000000000000000000000

Acetes paraguayensis 00000000000000000000000000000000000000000000000000

Acetes petrunkevitchi 00000000000000000000000000000000000000000000000000

Sicyonella inermis 00000000000000000000000000000000000000000000000000

Sicyonella maldivensis 01010110000000000100111000011000100000000000000000

Sicyonella antennata 01010110000000000100111000011000100000000000000000

Petalidium obesum 01011010000000000100111000010000100000000000000000

Petalidium foliaceum 01011010000000000100111000010000100000000000000000

Petalidium suspiriosum 01011010000000000100111000010000100000000000000000

Sergia tenuiremis 01010110000000101110011000100100000000000000000000

Sergia inoa 01010110000000101110011000100100000000000000000000

Sergia remipes 01010110000000101110011000100100000000000000000000

Sergia laminata 01010110000000101110011000100100000000000000000000

Gardinerosergia bigemmea 01010110000000101110011000100100000000000000000000

Gardinerosergia gardneri 01010100100000000110011000100100000000000000000000

Gardinerosergia inequalis 01010110000000101110011000100100000000000000000000

Gardinerosergia kensleyi 01010110000000101110011000100100000000000000000000

Gardinerosergia splendens 01010110000000000110011000100100000000000000000000

Phorcosergia bisulcata 01010100011000101110011000100100000000000000000000

Phorcosergia burukovskii 01010100011000101110011000100100000000000000000000

Phorcosergia filicta 01010100011000101110011000100100000000000000000000

Phorcosergia grandis 01010100011000101110011000100100000000000000000000

Phorcosergia maxima 01010110001000101110011000100100000000000000000000

Phorcosergia phorca 01010100011000101110011000100100000000000000000000

Phorcosergia plumea 01010100011000101110011000100100000000000000000000

Phorcosergia potens 01010100011000101100111000100100000000000000000000

Phorcosergia wolffi 01010100011000101110011000100100000000000000000000

Robustosergia extenuata 01110110001110101110011000100100000000000000000000

Robustosergia regalis 01110110001110101110011000100100000000000000000000

Robustosergia robusta 01110110001110101110011000100100000000000000000000

Robustosergia vityazi 01110110001110101110011000100100000000000000000000

Prehensilosergia prehensilis 01010101000000101110011000100100000000000000000000

Scintillosergia scintillans 01010110000000110000000000000000000000000000000000

Challengerosergia challengeri 10010110000001010101011000100100000000000000000000

Challengerosergia fulgens 10010110000001010101011000100100000000000000000000

Challengerosergia hansjacobi 10010110000001010101011000100100000000000000000000

Challengerosergia jeppeseni 10010110000001010101011000100100000000000000000000

Challengerosergia oksanae 10010110000001010101011000100100000000000000000000

Challengerosergia stellata 10010110000000000101011000100100000000000000000000

Challengerosergia talismani 10010110000001010101011000100100000000000000000000

Challengerosergia umitakae 10010110000001010110011000100100000000000000000000

Lucensosergia crosnieri 00011010000000000000011000100100100000000000000000

Lucensosergia colosii 10011010000000101110011000100100100000000000000000

Lucensosergia foresti 10011010000000000110011000100100100000000000000000

Lucensosergia lucens 10011010000000000110011000100100100000000000000000

Deosergestes coalitus 01010110000000101110011000100100000001101101001000

Deosergestes corniculum 01010110000000101110011000100100000001101101001000

Deosergestes disjunctus 01010110000000101110011000100100000001101101001000

Deosergestes henseni 01010110000000101110011000100100000001101101001000

Deosergestes paraseminudus 01010110000000101110011000100100000001101101001000

Deosergestes pediforms 01010110000000101110011000100100000001101101001000

Deosergestes rubroguttatus 01010110000000101110011000100100000001101101001000

Deosergestes seminudus 01010110000000101110011000100100000001101101001000

Eusergestes arcticus 01010110000000000110011000100100011100001101010000

Eusergestes similis 01010110000000000110011000100100011100001101010000

Eusergestes antarcticus 01010110000000000110011000100100011100001101010000

Sergestes atlanticus 10010101000000101110011000100010000000001010100000

Cornutosergestes cornutus 01011010000000000110000-00000000000000001010100100

Cornutosergestes mepae 01011010000000001110000-00000000000000001010100100

Allosergestes index 01000000000000101110011000100100000010001010100010

Allosergestes nudus 01000000000000101110011000100100010100001010100010

Allosergestes oleseni 01000000000000101110011000100100011000001010100010

Allosergestes pectinatus 01000000000000000110011000100100011000001010100010

Allosergestes pestafer 01000000000000101110011000100100010110001010100010

Allosergestes sargassi 01000000000000101110011000100100010110001010100010

Allosergestes verpus 01000000000000000110011000100100010110001010100010

Allosergestes vinogradovi 01000000000000000110011000100100010110001010100010

Parasergestes armatus 01011010000000110110010100100100000000001010100001

Parasergestes cylindricus 01011010000000110110010100100100000000001010100001

Parasergestes diapontius 01011010000000110110010100100100000000001010100001

Parasergestes halia 01011010000000110110010100100100000000001010100001

Parasergestes sirenkoi 01011010000000110110010100100100000000001010100001

Parasergestes stimulator 01011010000000110110010100100100000000001010100001

Parasergestes vigilax 01011010000000110110010100100100000000001010100001

Neosergestes brevispinatus 01010101000000110100110100100100000000001010100000

Neosergestes consobrinus 01010101000000110100110100100100000000001010100000

Neosergestes edwardsi 01010101000000110100110100100100000000001010100000

Neosergestes orientalis 01010101000000110100110100100100000000001010100000

Neosergestes semissis 01010110000000110100110100100100000000001010100000

Neosergestes tantillus 01010110000000110100110100100100000000001010100000

Characters 200 - 249

200 205 210 215 220 225 230 235 240 245

| | | | | | | | | |

Gennadas parvus 00000000000000000000000000000000000000000000000000

Penaeus monodon 00000000000000000000000000000000000000000000000000

Lucifer typus 00000000000000000000000000000000000000000000000000

Lucifer orientalis 00000000000000000000000000000000000000000000000000

Belsebub intermedius 00000000000000000000000000000000000000000000000000

Belsebub penicillifer 00000000000000000000000000000000000000000000000000

Belsebub faxoni 00000000000000000000000000000000000000000000000000

Belsebub chacei 00000000000000000000000000000000000000000000000000

Belsebub hanseni 00000000000000000000000000000000000000000000000000

Acetes americanus 00000000000000000000000000000000000000000000000000

Acetes binghami 00000000000000000000000000000000000000000000000000

Acetes chinensis 00000000000000000000000000000000000000000000000000

Acetes erythraeus 00000000000000000000000000000000000000000000000000

Acetes indicus 00000000000000000000000000000000000000000000000000

Acetes intermedius 00000000000000000000000000000000000000000000000000

Acetes japonicus 00000000000000000000000000000000000000000000000000

Acetes johni 00000000000000000000000000000000000000000000000000

Acetes natalensis 00000000000000000000000000000000000000000000000000

Acetes serrulatus 00000000000000000000000000000000000000000000000000

Acetes sibogae 00000000000000000000000000000000000000000000000000

Acetes vulgaris 00000000000000000000000000000000000000000000000000

Acetes marinus 00000000000000000000000000000000000000000000000000

Acetes paraguayensis 00000000000000000000000000000000000000000000000000

Acetes petrunkevitchi 00000000000000000000000000000000000000000000000000

Sicyonella inermis 00000000000000000000000000000000000000000000000000

Sicyonella maldivensis 00000000000000000000000000000000000000000000000000

Sicyonella antennata 00000000000000000000000000000000000000000000000000

Petalidium obesum 00000000000000000000000000000000000000000000000000

Petalidium foliaceum 00000000000000000000000000000000000000000000000000

Petalidium suspiriosum 00000000000000000000000000000000000000000000000000

Sergia tenuiremis 00000000000000000000000000000000000000000000000001

Sergia inoa 00000000000000000000000000000000000000000000000001

Sergia remipes 00000000000000000000000000000000000000000000000001

Sergia laminata 00000000000000000000000000000000000000000000000001

Gardinerosergia bigemmea 00110001101010101000100100110100011010100100101011

Gardinerosergia gardneri 00110001101010101000100100110100011010100100101011

Gardinerosergia inequalis 00110001101010101000100100110100011010100100101011

Gardinerosergia kensleyi 00110001101010101000100100110100011010100100101011

Gardinerosergia splendens 00110001101010101000100100110100011010100100101011

Phorcosergia bisulcata 00110001101010101000001011001001100110100011001011

Phorcosergia burukovskii 00110001101010101000001011001001100110100011001011

Phorcosergia filicta 00110001101010101000001011001001100110100011001011

Phorcosergia grandis 00110001101010101000001011001001100110100011001011

Phorcosergia maxima 00110001101010101000001011001001100110100011001011

Phorcosergia phorca 00110001101010101000001011001001100110100011001011

Phorcosergia plumea 00110001101010101000001011001001100110100011001011

Phorcosergia potens 00110001101010101000001011001001100110100011001011

Phorcosergia wolffi 00110001101010101000001011001001100110100011001011

Robustosergia extenuata 00110001101010101000010100101010010110100010101011

Robustosergia regalis 00110001101010101000010100101010010110100010101011

Robustosergia robusta 00110001101010101000010100101010010110100010101011

Robustosergia vityazi 00110001101010101000010100101010010110100010101011

Prehensilosergia prehensilis 00101001101010101000100100101100010110000010101011

Scintillosergia scintillans 00101001100110010100100100101100010101010010110101

Challengerosergia challengeri 00101010010110010010100100101100010101001010110101

Challengerosergia fulgens 00101010010110010010100100101100010101001010110101

Challengerosergia hansjacobi 00101010010110010010100100101100010101001010110101

Challengerosergia jeppeseni 00101010010110010010100100101100010101001010110101

Challengerosergia oksanae 00101010010110010010100100101100010101001010110101

Challengerosergia stellata 00101010010110010010100100101100010101001010110101

Challengerosergia talismani 00101010010110010010100100101100010101001010110101

Challengerosergia umitakae 00101010010110010010100100101100010101001010110101

Lucensosergia crosnieri 00101100010101010001100100101100010101001010110001

Lucensosergia colosii 00101100010101010001100100101100010101001010110001

Lucensosergia foresti 00101100010101010001100100101100010101001010110001

Lucensosergia lucens 00101100010101010001100100101100010101001010110101

Deosergestes coalitus 00000000000000000000000000000000000000000000000001

Deosergestes corniculum 00000000000000000000000000000000000000000000000001

Deosergestes disjunctus 00000000000000000000000000000000000000000000000001

Deosergestes henseni 00000000000000000000000000000000000000000000000001

Deosergestes paraseminudus 00000000000000000000000000000000000000000000000001

Deosergestes pediforms 00000000000000000000000000000000000000000000000001

Deosergestes rubroguttatus 00000000000000000000000000000000000000000000000001

Deosergestes seminudus 00000000000000000000000000000000000000000000000001

Eusergestes arcticus 00000000000000000000000000000000000000000000000001

Eusergestes similis 00000000000000000000000000000000000000000000000001

Eusergestes antarcticus 00000000000000000000000000000000000000000000000001

Sergestes atlanticus 10000000000000000000000000000000000000000000000001

Cornutosergestes cornutus 00000000000000000000000000000000000000000000000001

Cornutosergestes mepae 00000000000000000000000000000000000000000000000001

Allosergestes index 00000000000000000000000000000000000000000000000001

Allosergestes nudus 00000000000000000000000000000000000000000000000001

Allosergestes oleseni 00000000000000000000000000000000000000000000000001

Allosergestes pectinatus 00000000000000000000000000000000000000000000000001

Allosergestes pestafer 00000000000000000000000000000000000000000000000001

Allosergestes sargassi 00000000000000000000000000000000000000000000000001

Allosergestes verpus 00000000000000000000000000000000000000000000000001

Allosergestes vinogradovi 00000000000000000000000000000000000000000000000001

Parasergestes armatus 00000000000000000000000000000000000000000000000001

Parasergestes cylindricus 00000000000000000000000000000000000000000000000001

Parasergestes diapontius 00000000000000000000000000000000000000000000000001

Parasergestes halia 00000000000000000000000000000000000000000000000001

Parasergestes sirenkoi 00000000000000000000000000000000000000000000000001

Parasergestes stimulator 00000000000000000000000000000000000000000000000001

Parasergestes vigilax 00000000000000000000000000000000000000000000000001

Neosergestes brevispinatus 01000000000000000000000000000000000000000000000001

Neosergestes consobrinus 01000000000000000000000000000000000000000000000001

Neosergestes edwardsi 01000000000000000000000000000000000000000000000001

Neosergestes orientalis 01000000000000000000000000000000000000000000000001

Neosergestes semissis 01000000000000000000000000000000000000000000000001

Neosergestes tantillus 01000000000000000000000000000000000000000000000001
