# Supplementary material for: The shrimp superfamily Sergestoidea: a global phylogeny with definition of new families and an assessment of the pathways into principal biotopes
Source: R Soc Open Sci. 2017 Sep 6;4(9):170221. doi: 10.1098/rsos.170221 (PMC5627073; doi:10.1098/rsos.170221)
Supplement: Appendix 5. [file rsos170221supp5.doc]

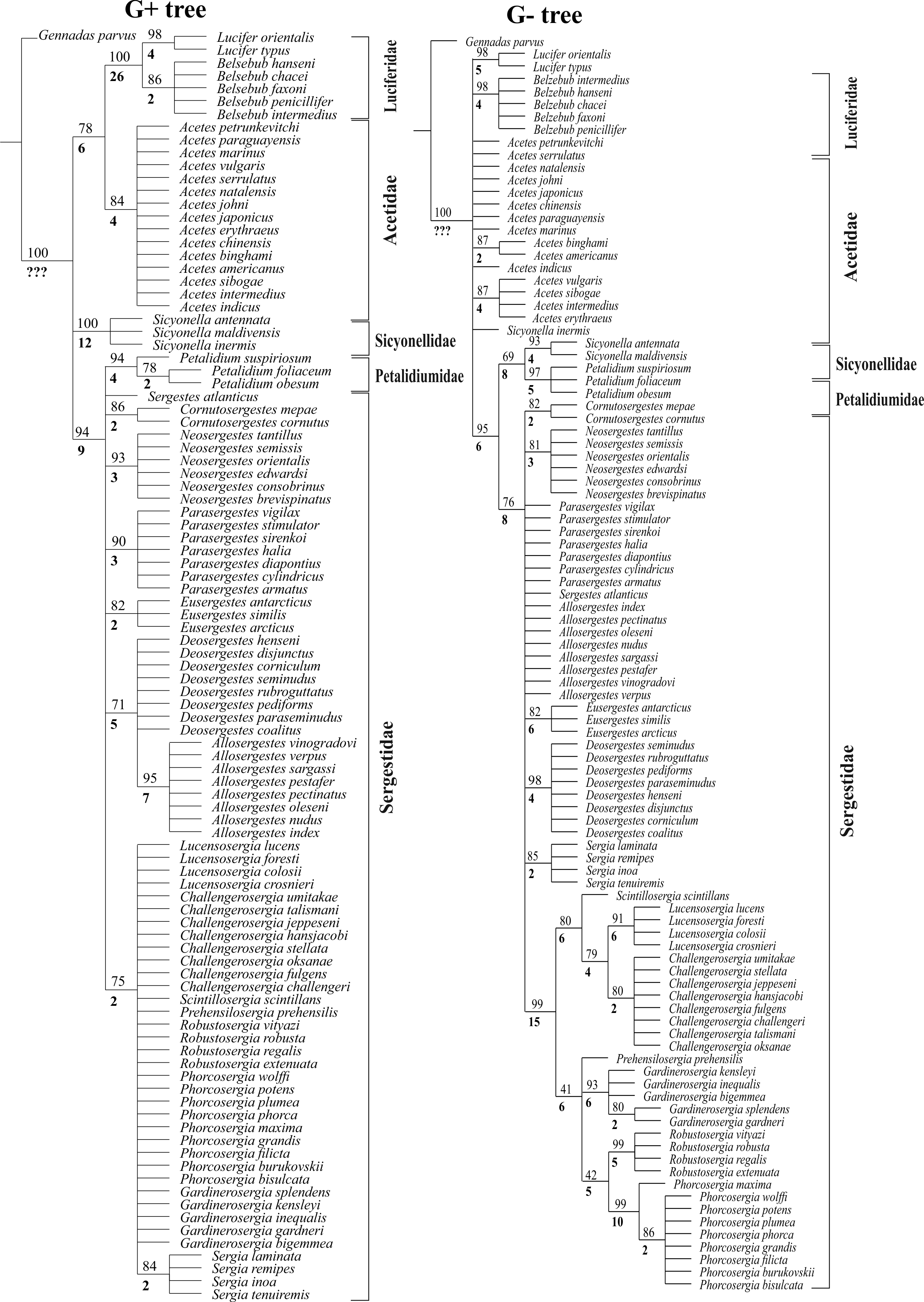


Appendix 5. The strict consensus tree and the clades retrieved after analysis with *Gennadas parvus* as the outgroup, with the bootstrap support (numbers above the clade) and the Bremer support (bold numbers below the clade): the G+ tree with only general external characters included in the matrix (left) and the G- tree with all but general external characters included in the matrix (right).
