# Supplementary material for: The shrimp superfamily Sergestoidea: a global phylogeny with definition of new families and an assessment of the pathways into principal biotopes
Source: R Soc Open Sci. 2017 Sep 6;4(9):170221. doi: 10.1098/rsos.170221 (PMC5627073; doi:10.1098/rsos.170221)
Supplement: Appendix 8 [file rsos170221supp8.docx]

Appendix 8. New and emended diagnoses and keys to major taxa of Sergestoidea Dana, 1852

SUPERFAMILY SERGESTOIDEA DANA, 1852

Diagnosis: Carapace compressed, rostrum shorter than eyestalks. Antennula with ventral flagellum modified to clasping organ in males or absent. Fourth and fifth pereopods either absent or flattened and natatory. Branchiae: pleurobranchs absent, two or less arthrobranchs on each side of somite, eight or less pairs of well-developed dendrite branchs on each side.

Key to families of the superfamily Sergestoidea

1. Labrum widely separated from antennae and eyes, telson with two pairs of lateral spines; second pereopod without chela, third pereopod with subchela. Males with two ventral processes on sixth abdominal somite, with strong ventral protuberance on telson, clasping organ absent . Luciferidae De Haan, 1849

- Labrum not widely separated from antennae and eyes, telson with four pairs of lateral spines or without spines; second and third pereopods with slightly reduced chela (2-3 times as long as fingers) 2

2. Pterygostomial tooth present, telson with four pairs of lateral spines, fourth and fifth pereopods with seven segments Sicyonellidae **fam. nov.**

- Pterygostomial tooth absent, telson without lateral spines, fourth and fifth pereopods with six or less segments 3

3. Third antennular segment elongate (>1.5 as long as first segment), fourth and fifth pereopods, if present, with five or less segments Acetidae **fam. nov.**

- Third antennular segment not elongate (<1.5 as long as first segment), fourth and fifth pereopods with six segments 4

4. Maximum height of rostrum at middle of its length. Male clasping organ bearing two opposite rows of serial bristles on inner side Petalidiumidae **fam. nov.**

- Maximum height of rostrum near tip. Serial bristles, if any, only on one side of male clasping organ Sergestidae Dana, 1852

FAMILY ACETIDAE **FAM. NOV.**

Diagnosis: Rostrum acute, with two or more dorsal teeth behind the orbital margin; carapace with postorbital and hepatic teeth, pterygostomial tooth absent, labrum not greatly separated from antennae and eyes; sixth abdominal somite in male without ventral processes; telson without lateral spines and ventral protuberance. Antennule in male with ventral flagellum and clasping organ bearing two tubercles; mandible and maxillae with palp, maxillula with a single endite; first maxilliped with epipod and exopod; second maxilliped with epipod, third maxilliped not elongated (less than twice as long as first pereopod), with entire dactyl; first to third pereopods with greatly reduced chela (palm >10 times as long as fingers), fourth and fifth pereopods reduced (1-5 segments) or absent. Photophores: absent. Petasma: reduced to some extent. Branchiae: arthrobranchs on somite VIII, developed anterior arthrobranch on somite XII, posterior arthrobranchs on somites IX-XII.

Genus included: *Acetes* H. Milne Edwards, 1830 (15 species).

Diagnosis of the genus and key to species may be found in [10]

FAMILY LUCIFERIDAE DE HAAN, 1849

Diagnosis: Rostrum short, acute, oblique; carapace greatly compressed laterally, with postorbital, pterygostomial, and hepatic teeth, labrum widely separated from antennae and eyes; sixth abdominal somite in male bearing two ventral processes; telson with two pairs of lateral spines, strong ventral protuberance present in male. Antennule without ventral flagellum and clasping organ; mandible and maxillae lacking palp, maxillula with two endites; first maxilliped lacking epipod and exopod, second maxilliped lacking epipod, third maxilliped not elongated (less than twice as long as first pereopod), with entire dactyl; with entire dactyl; first and second pereopods without chelae; third pereopod with subchela, distal end of propodus bearing strong, curved teeth and serrated setae; fourth and fifth pereopods absent in both sexes. Photophores: absent. Petasma: developed, pars astrigens absent, pars externa developed, transformed into a sheath around long, entire processus ventralis. Branchiae: absent.

Genera included: *Lucifer* Thompson, 1829 (2 species) and *Belzebub* Vereshchaka, Olesen and Lunina, 2016 (5 species).

Key to genera of the family Luciferidae

1. Posterior ventral process on sixth male abdominal somite curved, not tapering, apically obtuse. Eyestalks elongated, conical, nearly reaching end of scaphocerite. Petasma: sheath wide, entirely armed with transverse chitinous ribs along inner margin, without additional plate-like structures; processus ventralis lamellar, with apical pincer ……………..….……........ *Lucifer*

– Posterior ventral process on sixth male abdominal somite nearly straight, tapering, apically subacute. Eyestalks of moderate length, subcylindrical, not reaching end of scaphocerite. Petasma: sheath narrow, tapering, supported by strong chitinous ribs, armed with apical scales or of ridges, with additional plate-like structures; processus ventralis spiniform, without apical pincer .......................................................................................................................... *Belzebub*

Diagnoses of the genera and key to species may be found in [11].

FAMILY PETALIDIUMIDAE **FAM. NOV.**

Diagnosis: Rostrum acute, with a single dorsal tooth behind the orbital margin; carapace without postorbital and pterygostomial teeth, hepatic prominence as barb or tooth, labrum not greatly separated from antennae and eyes; sixth abdominal somite in male without ventral processes; telson without lateral spines and ventral protuberance. Antennule in male with ventral flagellum and clasping organ bearing two opposite rows of serial bristles on inner side; mandible and maxillae with palp, maxillula with three endites; first maxilliped with epipod and exopod; second maxilliped with epipod, third maxilliped not elongated (less than twice as long as first pereopod); second and third pereopods with greatly reduced chela (>10 times as long as fingers); fourth and fifth pereopods 6-segmented. Photophores: absent. Petasma: well-developed, capitulum armed with squamous hooks and pincers. Branchiae: arthrobranchs on somite VIII, rudimentary anterior arthrobranch on somite XII, rudimentary lamellar posterior arthrobranchs on somites IX-XI.

Genus included: *Petalidium* Spence Bate, 1881 (3 species).

Diagnosis of the genus and key to species may be found in [9].

FAMILY SERGESTIDAE DANA, 1852

Diagnosis: Rostrum 0-1 dorsal tooth behind the orbital margin; carapace without pterygostomial tooth, hepatic prominence as barb or tooth, labrum not greatly separated from antennae and eyes; sixth abdominal somite in male without ventral processes; telson without lateral spines and ventral protuberance. Antennule in male with ventral flagellum and clasping organ; mandible and maxillae with palp, maxillula with four endites; first maxilliped with epipod and exopod; second maxilliped with epipod, third maxilliped with subdivided dactyl; first pereopod without chela, second and third pereopods with greatly reduced chela (>10 times as long as fingers); fourth and fifth pereopods 6-segmented. Photophores: as dermal organs or organ of Pesta, or absent. Petasma: well-developed, capitulum armed with squamous hooks and pincers. Branchiae: podobranchs on somite VIII, developed anterior arthrobranch on somite XII, rudimentary lamellar posterior arthrobranchs on somites IX-XI.

Genera included: *Allosergestes* Judkins & Kensley, 2008 (8 species), *Challengerosergia* Vereshchaka, Olesen and Lunina, 2016 (8 species), *Cornutosergestes* Vereshchaka, Olesen and Lunina, 2016 (2 species), *Deosergestes* Judkins & Kensley, 2008 (8 species), *Eusergestes* Judkins & Kensley, 2008 (3 species), *Gardinerosergia* Vereshchaka, Olesen and Lunina, 2016 (5 species), *Lucensosergia* Vereshchaka, Olesen and Lunina, 2016 (4 species), *Neosergestes* Judkins & Kensley, 2008 (6 species), *Parasergestes* Judkins & Kensley, 2008 (7 species), *Phorcosergia* Vereshchaka, Olesen and Lunina, 2016 (9 species), *Prehensilosergia* Vereshchaka, Olesen and Lunina, 2016 (1 species), *Robustosergia* Vereshchaka, Olesen and Lunina, 2016 (4 species), *Scintillosergia* Vereshchaka, Olesen and Lunina, 2016 (1 species), *Sergestes* H. Milne-Edwards, 1830 (1 species), *Sergia* Stimpson, 1860 (4 species).

Key to genera of the family Sergestidae

1. Organ of Pesta absent. Body opaque in live specimens, or, if semi-transparent, with dermal photophores 2

- Organ of Pesta present. Body semi-transparent in live specimens, without dermal photophores 9

2. Integument membranous, dermal photophores absent *Sergia*

- Integument firm, dermal photophores present 3
3. Dermal photophores without lens, visible as opaque spots 4

- Dermal photophores with lens 6

4. Photophores as large, partly fused organs, arranged in 2 rows on scaphocerite and a triangular patch on uropodal exopod *Phorcosergia*

- Photophores small, not fused, arranged in 1 row on scaphocerite, and 1 row (randomly reduced to 1 organ) on uropodal exopod 5

5. Ocular papilla developed (>0.3 times as long as wide). LC of petasma without pillow at base, not twisted with LT, LT entire. Photophores on uropodal exopod positioned close to inner margin *Gardinerosergia*

- Ocular papilla rudimentary (<0.3 times as long as wide). LC of petasma with pillow at base, twisted with LT, LT divided. Photophores on uropodal exopod positioned close to median line *Robustosergia*

6. Photophores: in 2 lateral rows on carapace, 7 or more organs on scaphocerite 7

- Photophores: in a single lateral row on carapace, 6 or fewer organs on scaphocerite 8

7. Photophores: 7 organs on scaphocerite, 2 organs on proximal segment and 1 on distal segment of uropodal exopod. Petasma: LC divided, LI inflated, LT rudimentary, PV absent *Scintillosergia*

- Photophores: 10-15 organs on scaphocerite, 4-8 organs on proximal segment and 3-5 on distal segment of uropodal exopod. Petasma: LC entire, LI slender, LT well-developed, PV present *Prehensilosergia*

8. Photophores: 4-6 organs both on lateral carapace row and on scaphocerite. Petasma: PV without hooks and suckers *Challengerosergia*

- Photophores: 2-3 organs both on lateral carapace row and on scaphocerite. Petasma: PV with hooks and suckers *Lucensosergia*

9. Outer margin of uropodal exopod with tooth, not setose along proximal segment (proximal to the tooth) 10

- Outer margin of uropodal exopod without tooth, setose at least along part of proximal segment.. 12

10. First segment of antennule elongate, ≥1.5 times as long as 3rd segment, distal tooth of scaphocerite not overreaching blade, maxilliped III sexually dimorphic. Petasma: PU with hook, PV with simple spines. Arthrobranch: posterior lobe on segment XII (above pereopod III) dendritic *Eusergestes*

- First segment of antennule not elongate, <1.5 times as long as 3rd segment, distal tooth of scaphocerite overreaching blade, maxilliped III sexually not dimorphic. Petasma: PU without hook, PV unarmed. Arthrobranch: posterior lobe on segment XII (above pereopod III) lamellar 11

11. Rostrum triangular, not reaching middle of eyestalk. Endopod of maxilliped I with 3 segments. Petasma: LA rudimentary, LC developed, divided, PV present *Sergestes*

- Rostrum elongate, much overreaching middle of eyestalk. Endopod of maxilliped I with 2 segments. Petasma: LA developed, LC rudimentary, PV absent *Cornutosergestes*

12. Maxilliped III moderately elongated, < 2.0 times as long as carapace; chela of pereopod II with very long setae. Arthrobranch: posterior lobe on segment XII (above pereopod III) dendritic *Deosergestes*

- Maxilliped III much elongated, > 2.0 times as long as carapace; chela of pereopod II without very long setae. Arthrobranch: posterior lobe on segment XII (above pereopod III) lamellar 13

13. Rostrum with vertical frontal margin and beak-like terminal tooth, ocular papilla prominent, distal tooth of scaphocerite not overreaching blade, maxilliped III > 2.8 times as long as carapace, pereopod II without distally curved hooks on ischium, without protrusion on merus; chela with unequal fingers, pereopod III with strong curved spines proximal to tufts of long setae on propodus, pereopod V with distal segment setose along both margins. Petasma: LC absent, LI rudimentary, slender, PU with hook, PV developed *Allosergestes*

- Rostrum with oblique frontal margin, no beak-like terminal tooth, ocular papilla uncertain, distal tooth of scaphocerite much overreaching blade, maxilliped III 2.0-2.8 times as long as carapace, pereopod II with distally curved hooks on ischium and protrusion on merus; chela with subequal fingers, pereopod III without strong curved spines proximal to tufts of long setae on propodus, pereopod V with distal segment setose along one margin. Petasma: LC present, LI developed, inflated, PU without hook, PV rudimentary 14

14. Maxilliped III dactyl subdivided into 4 specialized subsegments, pereopod I with strong movable spines on ischium, outer margin of uropodal exopod setose partly *Parasergestes*

- Maxilliped III dactyl subdivided into 6 specialized subsegments, pereopod I without strong movable spines on ischium, outer margin of uropodal exopod setose entirely *Neosergestes*

Diagnoses of the genera and key to species may be found in [4].

FAMILY SICYONELLIDAE **FAM. NOV.**

Diagnosis: Rostrum acute, with two or more dorsal teeth behind the orbital margin; carapace with postorbital, pterygostomial, and hepatic teeth, labrum not greatly separated from antennae and eyes; sixth abdominal somite in male without ventral processes; telson with four lateral spines, ventral protuberance absent. Antennule in male with ventral flagellum and clasping organ; mandible and maxillae with palp, maxillula with two endites; first maxilliped with epipod and exopod; second maxilliped with epipod, third maxilliped elongated (at least twice as long as first pereopod) with dactyl subdivided into four specialized subsegments; first pereopod with developed chela (palm as long as fingers), second and third pereopods with slightly reduced chela (2-3 times as long as fingers); fourth and fifth pereopods 7-segmented. Photophores: dermal organs and organ of Pesta absent. Petasma: well-developed, capitulum armed with squamous hooks and pincers. Branchiae: arthrobranchs on somite VIII, developed anterior arthrobranch on somite XII, reduced dendritic posterior arthrobranchs on somites IX-XI.

Genus included: *Sicyonella* Borradaile, 1910 (3 species).

Diagnosis of the genus and key to species may be found in [10].
